# The BRCT domain enhances DNA binding and catalytic efficiency of fungal PARPs

**DOI:** 10.1038/s41467-026-75844-6

**Published:** 2026-07-24

**Authors:** Adam G. Bainbridge, Tiberiu-Marius Gianga, Rohanah Hussain, Callum Parkin, Adam D. Longhurst, Nicolas Helmstetter, Rhys A. Farrer, Sandra Catania, Giuliano Siligardi, Johannes Gregor Matthias Rack

**Affiliations:** 1 Medical Research Council Centre for Medical Mycology at the University of Exeter, Department of Biosciences, Faculty of Health and Life Sciences, Geoffrey Pope Building, Stocker Road, Exeter, EX4 4QD, UK; 2 Beamline 23, Diamond Light Source Ltd, Harwell Science and Innovation Campus, Didcot, OX11 0DE, UK

**Keywords:** DNA, DNA damage response, Enzyme mechanisms

## Abstract

*Aspergillus fumigatus* infections are a major yet often neglected global health challenge magnified by a growing at-risk population, limited treatment options, and the emergence of drug-resistant strains. Regulation of the DNA damage response (DDR) by ADP-ribosylation signalling has recently emerged as an important feature of fungal pathogenesis, but the underlying mechanisms remain largely elusive. Here we present a comprehensive phylogenetic and functional characterisation of *Af*-PARP1, the *A. fumigatus* PARP homologue. Our data reveal *Af*-PARP1 as a DNA-dependent poly(ADP-ribosyl)transferase with unique domain architecture, DNA damage selectivity, and activation dynamics distinct from its mammalian and plant homologues. We show that the fungal specific BRCT domain plays a crucial role in both damage recognition and ADP-ribosylation signal establishment. Collectively, our findings reveal a divergence in DDR-associated ADP-ribosylation specific to fungi, highlighting the potential of this signalling pathway as target for antifungal therapy.

## Abstract (web summary)

*Aspergillus fumigatus* is a growing global health threat urgently requiring novel interventions. Here the authors present a comprehensive phylogenetic and functional analysis of *Af*-PARP1, a key DNA damage response regulator, and highlight exploitable differences with mammalian and plant homologues.

## Introduction

Fungal diseases, though historically neglected, are increasingly recognised as a growing global human health concern with current estimates of 3.8 million deaths annually from 6.5 million invasive fungal infections^1^. In response to this rising threat, the World Health Organization recently published a fungal priority pathogens list to guide research and public health intervention^2^. *Aspergillus fumigatus* (*Af*), the causative agent of invasive Aspergillosis (IA), is ranked in the highest priority group due to its prevalence, high mortality rate, limited treatment options and the rapid emergence of antifungal resistance^2–4^. *Af* is a ubiquitous saprotrophic fungus with vegetative mycelia life that is found in the environment, primarily associated with decaying organic matter in soils. *Af* spores are routinely inhaled, but disease manifestation depends largely on the immune status of the host. While healthy individuals efficiently clear the infection, immunocompromised patients, such as those with neoplastic disease, transplant recipients and superinfections associated with pulmonary tuberculosis or severe viral pneumonia (e.g. caused by influenza or SARS-CoV-2) are at high risk of developing IA^5–7^. These *Aspergillus* infections are associated with poor patient outcome and remain a leading cause of mortality in immunocompromised patients^1,6,8^.

The maintenance of genome stability is essential for cellular viability and function and is preserved across all kingdoms of life through highly conserved mechanisms, including DNA repair. In microbial pathogens, including human pathogenic fungi, accumulation of genetic mutations can, however, also drive in-host microevolution and the emergence of antifungal drug resistance. Studies in *Af* have shown that mutation of *msh6*, which is part of the DNA mismatch repair (MMR) pathway, leads to an increased basal mutation rate and can promote the emergence of drug-resistance^9,10^. Importantly, unlike classical hypermutator strains, the *msh6* mutant does not exhibit detectable fitness defects, suggesting a selective advantage for persistence and evolution under antifungal selection pressure. A similar effect has also been described in the human pathogenic yeast *Cryptococcus deuterogattii*, where nonsense mutations in the MMR gene *msh2* result in increased mutation rates and facilitate the rapid inactivation of antifungal drug target genes^11^.

To maintain the balance between adaptive benefits and cell viability, fungal pathogens need to mount a tightly controlled DNA damage response (DDR). Targeting these pathways may reveal novel vulnerabilities that offer promising avenues for developing next-generation antifungal therapies to improve human health.

In higher eukaryotes, a critical regulator of the DDR is ADP-ribosylation (ADPr), a versatile post-translational modification also associated with the regulation of other crucial cellular processes, such as transcriptional regulation, immunity and microbial metabolism, amongst others^12–17^. The modification is carried out by (ADP-ribosyl)transferases (ARTs) termed PARPs that transfer the ADP-ribosyl moiety from the metabolic cofactor NAD^+^ onto various targets, including proteins and nucleic acids^14,17,18^. In proteins, ADP-ribose can be attached to a variety of functional groups within amino acid side chains, including acidic (Glu/Asp), basic (Arg/Lys), hydroxyl (Ser/Tyr) and thiol (Cys) functionalities^14,19^. Some PARPs, termed polyARTs, can extend the initial mono-ADP-ribosyl modification (MARylation) to form ADP-ribose polymers (PARylation). Three human PARPs (*h*PARP1, 2 and 3) are involved in DDR signalling with *h*PARP1 and 2, both PARylating enzymes, making up the bulk of DNA damage-dependent PARylation in cells (depending on model ~90% by *h*PARP1 and 10-15% by *h*PARP2)^20,21^. The modification establishment requires an intricate inter- and intramolecular interplay where *h*PARP1/2 detects and becomes activated by direct recognition of DNA damage sites and catalyses the formation of a localised PARylation signal, which triggers the recruitment of downstream repair factors. Specifically, recognition of damaged DNA by designated DNA-binding domains (ZnF1/ZnF2 and the WGR in *h*PARP1 and *h*PARP2, respectively) induces self-assembly of the enzyme onto the site of DNA damage^22^. This leads to local destabilisation of the auto-inhibitory domain (HD), which results in the enzyme adopting a catalytically active conformation where NAD^+^ can access the active site within the ART domain^23^. In addition, this conformational change allows for the formation of a composite active site, achieved through binding of histone PARylation factor 1 (HPF1), which shifts the enzyme’s modification specificity from glutamate/aspartate to serine residues^24–26^. Importantly, serine-linked ADP-ribosylation has been shown to make up the major fraction of ADP-ribosylation post DNA damage, highlighting its crucial physiological role in responding to DNA damage-inducing stresses^16,27^.

**Fig. 1:**
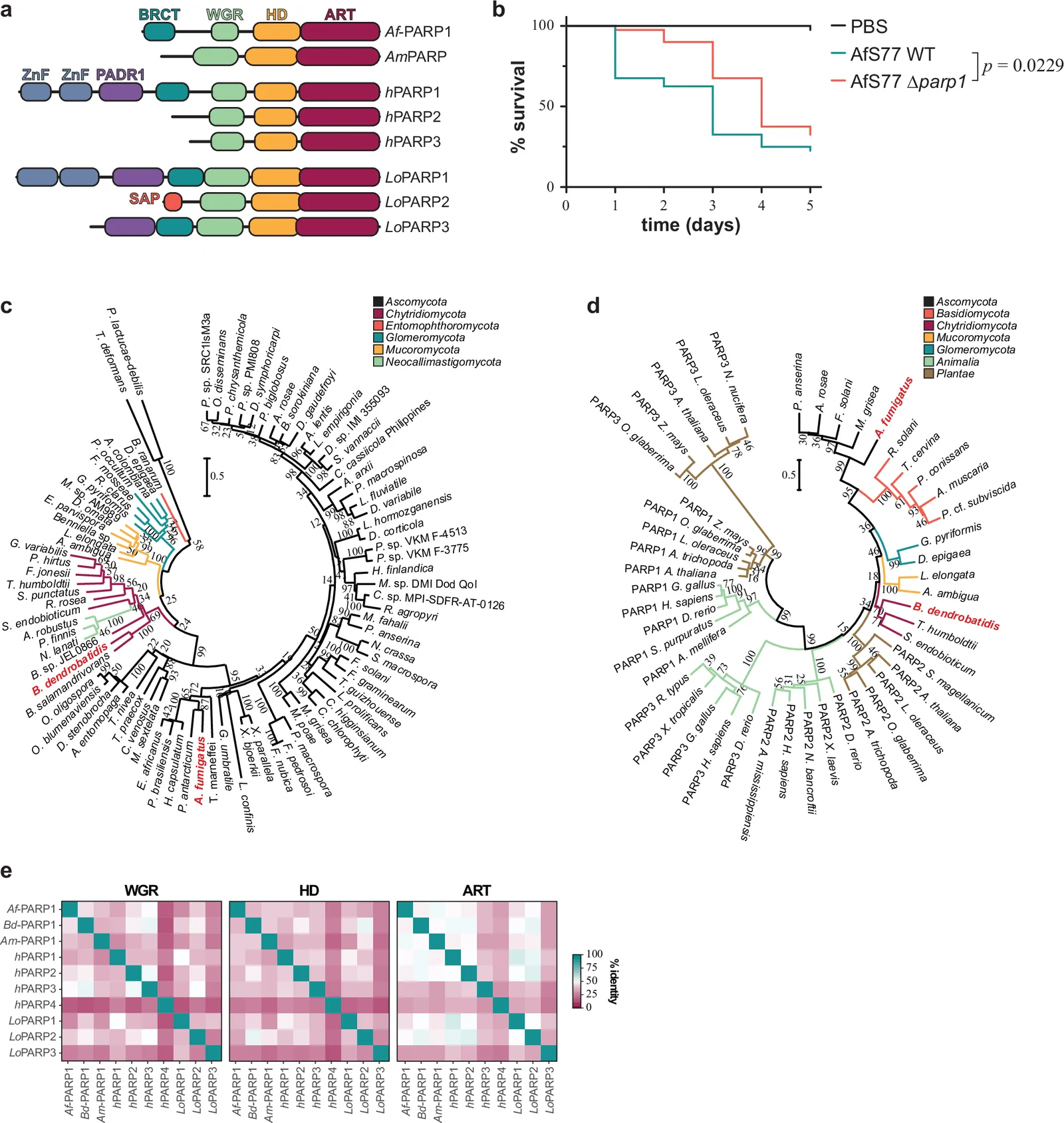
Fungal PARPs are phylogenetically distinct from their homologues in *Animalia* and *Plantae.* **a** Schematic respresentation of DDR PARPs from *Fungi*, *Animalia,* and *Plantae* as represented by *A. fumigatus*, *Amanita muscaria* (*Am*), *H. sapiens,* and *Lathyrus oleraceus* (*Lo*), respectively. BRCT, breast cancer type 1 susceptibility protein (BRCA1) C-terminal domain; HD, alpha-helical regulatory domain; PADR1, zinc-binding domain found in PARP1 and its homologues (also known as the third zinc-binding domain [also termed ZnF3]^106,107^); SAP, after SAF-A/B, Acinus and PIAS (Note, the number of SAP domains within plant PARP2s is variable^108^); WGR, after conserved Trp-Gly-Arg motif; ZnF, PARP-type zinc fingers. **b** Kaplan–Meier survival analysis and log-rank test of *Galleria mellonella* larvae infected with 10^5^ conidia of the indicated strains in PBS or PBS alone. Data represent four biological replicas with *n* = 10 larvae per group per replica. **c**, **d** Evolutionary phylogenetic tree analysis of *Af*-PARP1-like proteins in the fungal kingdom (Supplementary Table 1b) and DDR PARPs across the *Animalia*, *Fungi,* and *Plantae* kingdoms (Supplementary Table 2c). For the latter, the tree was constructed taking only the WGR-HD-ART region into account, with amino acid sequences isolated from their whole protein context by multiple sequence alignment. The evolutionary histories were inferred by using the maximum likelihood method and the LG model of amino acid substitution as implemented in MEGA 11. The trees with the highest log likelihoods (-61369.22 [b] and -27410.93 [c], respectively) are shown. The trees are drawn to scale, with branch lengths measured in the number of substitutions per site. Phylogenetic divisions and kingdoms are coloured as indicated. **e** Pairwise identity matrices were calculated from multiple sequence alignments of the individual domains using Biostrings^103^ and visualised using heatmapper^104^.

Recent studies demonstrated a role of fungal PARPs and associated protein PARylation in the regulation of fungal pathogenicity^28–31^. Moreover, a PARP homologue of *Aspergillus nidulans* was shown to be associated with the early DDR as well as sensitivity to various DNA-damaging agents upon reduced PARP expression^32^. However, the underlying physiological function leading to reduced virulence of PARP knockouts, as well as the molecular details regarding fungal PARP activation and regulation, remain elusive. Noteworthy, fungal PARPs have a distinct domain architecture compared to their animal and plant homologues (Fig. 1a). Therefore, determining similarities and differences between these and plant/animal PARPs is crucial to assess the therapeutic potential of ADP-ribosylation signalling as a target to treat fungal diseases.

Here we report that fungal PARPs share a conserved allosteric activation mechanism with human PARP1/2/3, but preferentially respond to single-strand DNA breaks carrying a 5′ phosphate group. Unlike *h*PARP2, *Af*-PARP1 is not activated by blunt-end or double-strand breaks. Their unique BRCT domain contributes directly to DNA binding and chromatin association through lysine-mediated interactions and enhances catalytic efficiency on specific DNA damage structures, likely by stabilising the association of *Af*-PARP1 with the DNA lesion.

## Results

### DNA damage-responsive PARPs within the *Fungi* kingdom

To date, ADP-ribosylation signalling in the DDR has primarily been studied in mammals as well as some limited work in plants, while its importance for fungal (patho)physiology remained largely overlooked^28^. However, recent results indicate that fungal PARP1/2-like proteins, e.g. from *Magnaporthe oryzae* and *Fusarium oxysporum* f. sp. *niveum*, contribute to their phytopathology^28–31^. To assess whether *Af*-PARP1 has a similar role in infections of animals, we constructed an *Af*-*parp1* knock-out strain (AfS77 Δ*parp1*) and conducted survival analyses using a *Galleria mellonella* infection model (Fig. 1b). Indeed, AfS77 Δ*parp1* shows significantly attenuated virulence compared to the parental strain, most notably at early infection timepoints, thus supporting a role of *Af*-PARP1 in the establishment of fungal pathogenicity.

Since the reported physiological roles for fungal PARPs differ widely and so far did not involve a direct link to the DNA damage response^28–31^, we set out to investigate the molecular mechanism of *Af*-PARP1 activation and regulation to determine similarities and differences to the homologues in animals and plants.

BLASTp searches^33^ using known human and fungal PARP family proteins^17,34,35^ as queries and the NR database on NCBI revealed three PARP-like proteins with distinct domain architectures in *A. fumigatus*: *Af*-PARP1 (AFUB_054870), a homologue of human PARP1/2, *Af*-PARP2 (AFUB_070850), a homologue of human PARP6/8, and *Af*-TPT1 (AFUB_019970) a homologue of tRNA 2’-phosphotransferase TRPT1 (Fig. 1a and Supplementary Fig. 1). Based on domain prediction and earlier work in *A. nidulans*^32,34^, only *Af*-PARP1 (also termed prpA in *A. nidulans* and NPO in *Neurospora crassa*) is predicted to be involved in the DDR. In addition, mammalian DDR-associated ADP-ribosylation is dependent on the auxiliary factor HPF1, which alters the *h*PARP1/2 catalytic site and shifts target specificity from glutamate/aspartate residues to primarily serine residues^24,25,36,37^. Sequence (BLAST) and structural (FoldSeeker^38^) searches did not reveal any HPF1 homologues within the *Fungi* kingdom, indicating DDR ADP-ribosylation signalling relies on *Af*-PARP1 alone. We further analysed the distribution of *Af*-PARP1-like proteins within the fungal kingdom and found that sequence diversity followed species phylogeny (Fig. 1c and Supplementary Tab. 1). Amongst the *Ascomycota*, PARPs are exclusively identified in the *Pezizomycotina* subdivision and in two species within the *Taphrinales*: *Protomyces lactucae-debilis* and *Taphrina deformans* (Fig. 1c). However, the PARPs of the latter species cluster more closely with sequences derived from the *Entomophthoromycota* and *Glomeromycota* rather than other *Ascomycota*, suggesting that they may have been re-acquired through horizontal gene transfer rather than maintained throughout evolution of this lineage. While common within the *Basidiomycetes*, PARP1/2-like proteins are also absent in some dimorphic species, including the human pathogen *Cryptococcus neoformans* and the plant pathogens *Melampsora lini* and *Mycosarcoma maydis*.

The domain architecture of the fungal PARP1/2 homologues revealed the absence of the zinc finger domains found in *h*PARP1, while the BRCT domain, absent in *h*PARP2, is retained (Fig. 1a). Noteworthy, the BRCT domain is absent within the *Basidiomycota* division, but *Basidiomycota* PARPs still form a sister group to the *Ascomycota* PARPs, thus indicating a loss of the BRCT domain in the early *Basidiomycota* evolution (Fig. 1b, d and Supplementary Table 2). Comparison of fungal PARPs with *Animalia* and *Plantae* DDR PARPs revealed that fungal PARPs cluster more closely with zinc finger-independent PARPs. As such, PARP2 from animals and plants and PARP3 from animals are the closest related homologues, while animal and plant PARP1-like proteins form sister groups and plant PARP3s are only distantly related (Fig. 1d). The divergence of plant PARP3s may be due to the apparent loss of catalytic activity and thus adherence to different evolutionary pressures^39,40^. Conservation analysis of the single domains revealed that the WGR domain largely follows the overall phylogenetic analysis, but that ART domains of fungal PARPs show closer homology to *h*PARP1/2 and garden pea PARP2 (*Lathyrus oleraceus* [formerly *Pisum sativum*]; *Lo*PARP2) rather than *h*PARP3 (Fig. 1e). Since earlier observations showed that the WGR domains of *h*PARP2/3 are key in determining which DNA damage structures activate the respective enzymes^41,42^, this might be indicative for a more *h*PARP3-like activation pattern combined with the ability to form ADP-ribose polymers.

### *Af*-PARP1 is a DNA damage-activated poly(ADP-ribosyl)transferase with a preference for ssDNA breaks

**Fig. 2:**
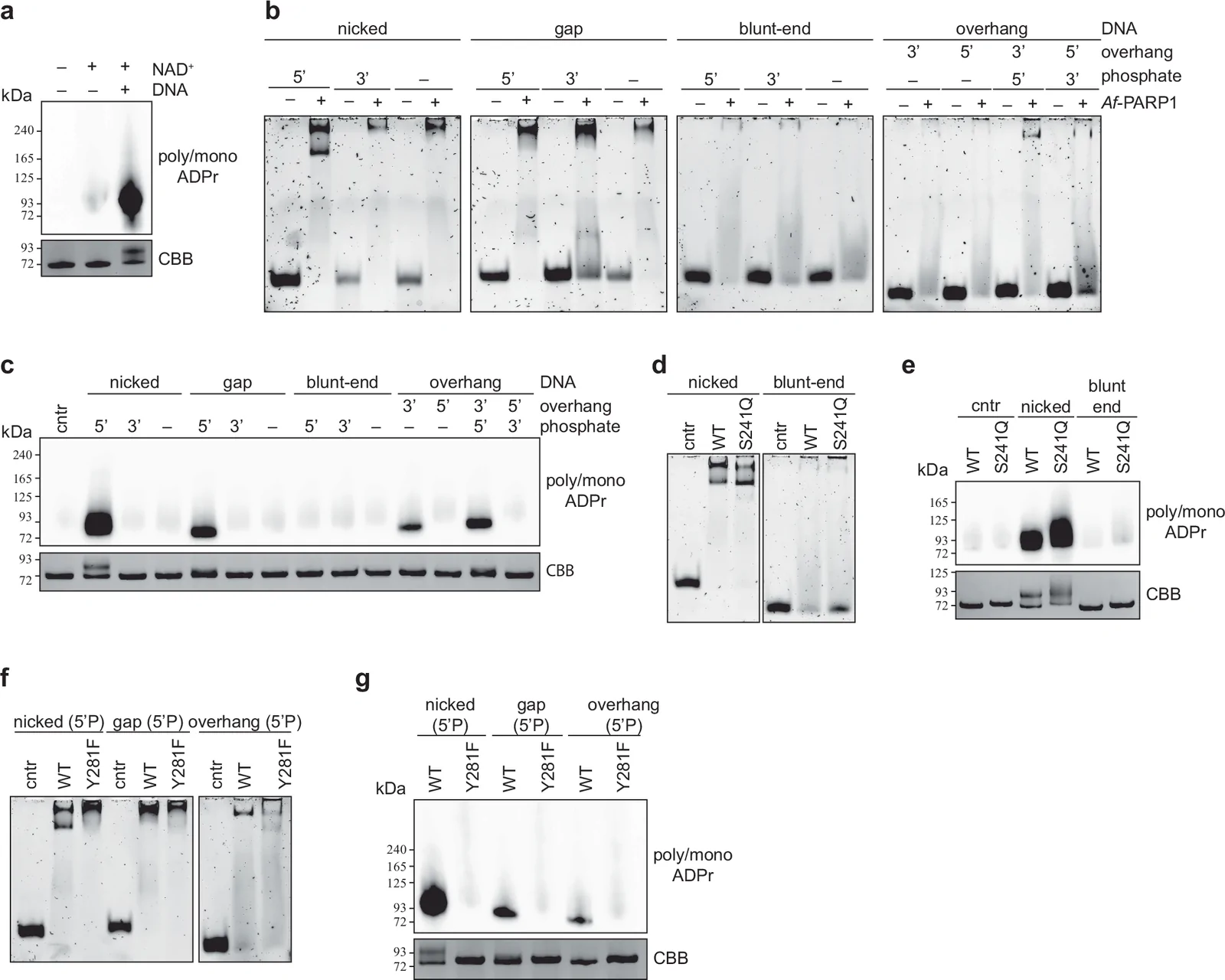
*Af*-PARP1 is a DNA-activated poly(ADP-ribosyl)transferase. **a** Catalytic activity of *Af*-PARP1 was assessed in response to damaged DNA. *Af*-PARP1 (0.5 μM) was incubated with NAD^+^ (500 μM) in the presence of activated DNA (BPS Bioscience), and the formation of PAR was detected using immunoblot. Loading was visualised by Coomassie Brilliant Blue (CBB) staining. Shown is a representative result of three independent experiments. Uncropped images are available in the Source Data file. **b** EMSA assay assessing the DNA damage binding specificity of *Af*-PARP1. *Af*-PARP1 (5 μM) was complexed with DNA mimetics (1 μM), separated via native PAGE and visualised with SYBR safe staining. Shown is a representative result of three independent experiments. Uncropped images are available in the Source Data file. **c** Assessment of *Af*-PARP1 catalytic activity exposed to different DNA damage mimetics. Shown is a representative result of three independent experiments. Uncropped images are available in the Source Data file. **d** EMSA assay assessing the impact of Ser241 on the ability of *Af*-PARP1 to interact with 5′-phosphorylated nicked (DNA-1) and blunt-end (DNA-5) DNAs. Assay conditions were as described in (**b**). Shown is a representative result of two independent experiments. Uncropped images are available in the Source Data file. **e** Auto(ADP-ribosylation) assay assessing the activity of *Af*-PARP1 S241Q in the presence of 5′-phosphorylated nicked or blunt-end DNAs. Assay conditions are as described in (**c**). Shown is a representative result of two independent experiments. Uncropped images are available in the Source Data file. **f** EMSA assay assessing the impact of Tyr281 on the ability of *Af*-PARP1 to interact with 5′-phosphorylated nicked (DNA-1), gap (DNA-2) and 3′ overhang (DNA-9) DNAs. Assay conditions were as described in (**b**). Shown is a representative result of two independent experiments. Uncropped images are available in the Source Data file. **g** Auto(ADP-ribosylation) assay assessing activity of *Af*-PARP1 S241Q in the presence of 5′-phosphorylated nicked, gap or 3′ overhang DNAs. Assay conditions are as described in (**c**). Shown is a representative result of two independent experiments. Uncropped images are available in the Source Data file.

Despite the link between *Af*-PARP1 and fungal virulence, the underlying mechanistic connection to the DDR, if any, remained elusive. Therefore, we set out to characterise the mode of *Af*-PARP1 activation and intramolecular regulation to determine similarities and differences to mammalian and plant PARPs. First, we characterised the ability of *Af*-PARP1 to bind DNA and assessed the influence on ART domain activation and compared these with closely related PARPs; *h*PARP2 and 3 and *Lo*PARP2. *Af*-PARP1 has neglectable activity in the absence of DNA but exhibits strong auto(ADP-ribosyl)ation in the presence of damaged genomic DNA (Fig. 2a). As *Af*-PARP1 smearing was less pronounced in the immunoblot than expected for a *h*PARP1/2-like polyART and the Coomassie Brilliant Blue (CBB) stained SDS-PAGE showed a distinct upwards shifted band, we aimed to investigate whether *Af*-PARP1 produced primarily a MAR or PAR modification. Sequence alignment of the HD-ART domain of fungal, animal and plant PARPs showed that the donor loop (D-loop) of the ART domain, which is critical for chain elongation in polyARTs^19,43^, closely resembles animal and plant PARP1/2, whereas it is shortened in animal PARP3s (Supplementary Fig. 2 and Supplementary Table 3). Moreover, *Af*-PARP1 contains the proline triple (Pro542, Pro543, and Pro546), Tyr550, and Met551, which are hallmarks of the ability to form polymers^43–45^, hence suggesting that the signal generated by *Af*-PARP1 are short polymers. To confirm this hypothesis, we utilised a panel of well-characterised anti-ADP-ribosylation antibodies and detection reagents^46–48^ as well as generated an E657Q mutant, analogous to the elongation-deficient, but MARylation-capable, *h*PARP1 E988Q (Supplementary Fig. 3a). Together, these experiments indicate that *Af*-PARP1 WT produces primarily a PAR signal, with some potential contribution of MARylation. As predicted, the E657Q mutant was limited to auto-MARylation. To determine the chemical nature of the formed protein-ADP-ribose bond, we utilised a set of (ADP-ribosyl)hydrolases with known linkage-specificities^49–51^. Since most ARH- or macrodomain-type hydrolases can only cleave the terminal linkage in the absence of an ADP-ribose polymer^46,49,52^, reactions were performed on the automodified E657Q mutant as well as WT co-treated with PARG to convert the WT-derived PARylation into MARylation (Supplementary Fig. 3b, c). While PARG could efficiently degrade the ADP-ribose polymer, only the glutamate/aspartate-specific enzymes human MacroD1, TARG1 and *Staphylococcus aureus* Zn-dependent macrodomain (*Sau*Macro) showed activity against the MAR modification. In addition, *Af*-PARP1 automodification is sensitive to both alkaline pH and neutral hydroxylamine (Supplementary Fig. 3d). Together, these observations strongly support linkage via an acetal ester, suggesting that the PAR chains are attached to glutamate and/or aspartate residue(s)^53,54^.

To dissect the DNA binding and activation profile of *Af*-PARP1 in detail, we constructed a synthetic DNA damage mimetics library (Supplementary Tab. 4) comprised of oligonucleotides representing important cellular DNA damage structures, with and without 5′- or 3′-phosphorylation. *Af*-PARP1 preferentially binds to single-stranded nicked (DNA-1, -12, and -11) and gap (DNA-2, -3, and -13) structures, while binding to blunt-end (DNA-5, -6, and -4) and overhang structures was generally weaker (Fig. 2b). Interestingly, *Af*-PARP1 showed modest binding to 5′-phosphoylated 3′-overhang DNA (DNA-9) compared to its non-phosphorylated counterpart (DNA-7) suggesting that a 5′-terminal phosphate may contribute to stabilising interactions between *Af*-PARP1 and damaged DNA. While the *Af*-PARP1:DNA interaction with 5′-phosphoylated 3′-overhangs appears to be independent of overhang length (Supplementary Fig. 4a), *Af*-PARP1 displayed only marginal binding to ssDNA (Supplementary Fig 4b), thus suggesting that the dsDNA part the overhang structure, rather than the overhang itself, is the primary determinant of binding. As suggested by our sequence analysis (Fig. 1d), this binding profile is similar to *h*PARP3 and stands in contrast to *h*PARP2 and *Lo*PARP2, which bind robustly to 5′-phosphorylated blunt-end structures (Supplementary Fig. 4c).

We next assessed whether DNA binding translated into catalytic activation of *Af*-PARP1. The (ADP-ribosyl)transferase activity of *Af*-PARP1 was selectively induced by DNA substrates containing a 5′-phosphate group but only when coupled with permissive structural contexts, such as nicked (DNA-1), gap (DNA-2), or 3′-overhang DNA (DNA-9; Fig. 2c). In the case of overhang substrates, activation required a minimum of four nucleotides in the 3′-overhang, as shorter extensions (1- or 3-nt) or ssDNA failed to elicit activity, whereas substrates with ≥4-nt extensions (DNA-9, -16/17.5, and -16/17.11) supported auto-PARylation (Fig. 2c and Supplementary Fig. 4d, e).

No activity, with the exception of unphosphorylated 3′-overhang DNA (DNA-7), was observed with unphosphorylated or 3′-phosphorylated variants of these substrates, mirroring the 5′-phosphate-dependent activation pattern observed in other zinc finger independent PARPs such as *h*PARP2^55^, *h*PARP3^41^, and *Lo*PARP2 (Supplementary Fig. 4f). Together these results establish that *Af*-PARP1 is catalytically responsive to DNA damage but despite its closer phylogenetic and ART domain relation with *Animalia* and *Plantae* PARP2s, displays a DNA damage binding and activation profile more closely resembling animal PARP3 (Fig. 1c, d). This divergence in binding and activation preferences reflect unique aspects of fungal PARP regulation in DNA damage sensing.

### The WGR domain of *Af*-PARP1 shows divergent DNA-binding regulation

DNA damage recognition by *h*PARP2 has been well characterised both biochemically and structurally, and crystal structures capturing the protein DNA interaction have identified key residues within its WGR domain^42^. Despite strong sequence and structural similarity, *Af*-PARP1 exhibits a distinct DNA substrate preference, notably lacking the ability to bind and be activated by blunt-end DNA structures (Fig. 2b, c). Sequence alignment of the WGR domains from *Af*-PARP1 and *h*PARP2 (Supplementary Fig. 5a) reveals a high degree of conservation, with eight out of the nine key DNA-binding residues preserved in *Af*-PARP1. Structural alignment of the *Af*-PARP1 WGR AlphaFold 3 model with the WGR domains of *h*PARP2 and 3 showed a high degree of similarity with an r.m.s.d. of 0.569 Å over 80 C^α^ and 1.248 Å of 83 C^α^, respectively (Supplementary Fig. 5b, c). The single non-conserved residue (Ser241 in *Af*-PARP1 and Gln159 in *h*PARP2) has been implicated in *h*PARP2 activation by DSB structures, where its mutation (Q159A) abrogates catalytic activation by blunt-end but not nicked DNA^55^. To assess whether this difference accounts for *Af*-PARP1’s inability to be activated by blunt-end DSBs, we generated the *Af*-PARP1 S241Q mutant. This mutation neither restored binding to the blunt-end DSB mimetic nor exhibited increased auto-PARylation activity in its presence (Fig. 2d, e). To confirm that the lack of activity from DSB structures was not a result of the hairpin loop or short DNA length of the blunt-end DSB mimetic (DNA-5; Supplementary Table 4), we assessed binding and activity in response to a longer 5′-phosphorylated duplex DNA (DNA-16/17; Supplementary Table 4). Although the DNA duplex bound to both *Af*-PARP1 wild-type (WT) and *Af*-PARP1 S241Q, it did not support significant auto-PARylation of either enzyme (Supplementary Fig. 5d, e). These results indicate that although *Af*-PARP1 shares overall structural conservation with *h*PARP2, it could employ a regulatory mechanism for DNA binding that differs from that described for its human counterpart.

In the zinc finger-independent PARPs, including *h*PARP2 and *h*PARP3, catalytic activation in response to 5′-phosphorylated DNA breaks is mediated by a conserved tyrosine residue (Tyr201 in *h*PARP2) forming a hydrogen bond with the 5′-phosphate moiety at the break site (Supplementary Fig. 5f)^42^. This residue is conserved across eukaryotic PARPs lacking zinc finger domains, including *h*PARP3, plant PARP2s, and *Af*-PARP1 (Tyr281 in *Af*-PARP1; Supplementary Fig. 5a, g). In contrast, *h*PARP1 contains a phenylalanine at the corresponding position, reflecting the different DNA binding mechanisms used by *h*PARP1. Consistent with this model, mutation of Tyr281 in *Af*-PARP1 (Y281F) abolished catalytic activation in response to 5′-phosphorylated DNA while preserving DNA binding capacity of the mutant (Fig. 2f, g). These findings reinforce the functional conservation of the 5′-phosphate-sensing mechanism across zinc finger-independent PARPs.

### *Af*-PARP1 shares an allosteric activation mechanism with human PARPs

**Fig. 3:**
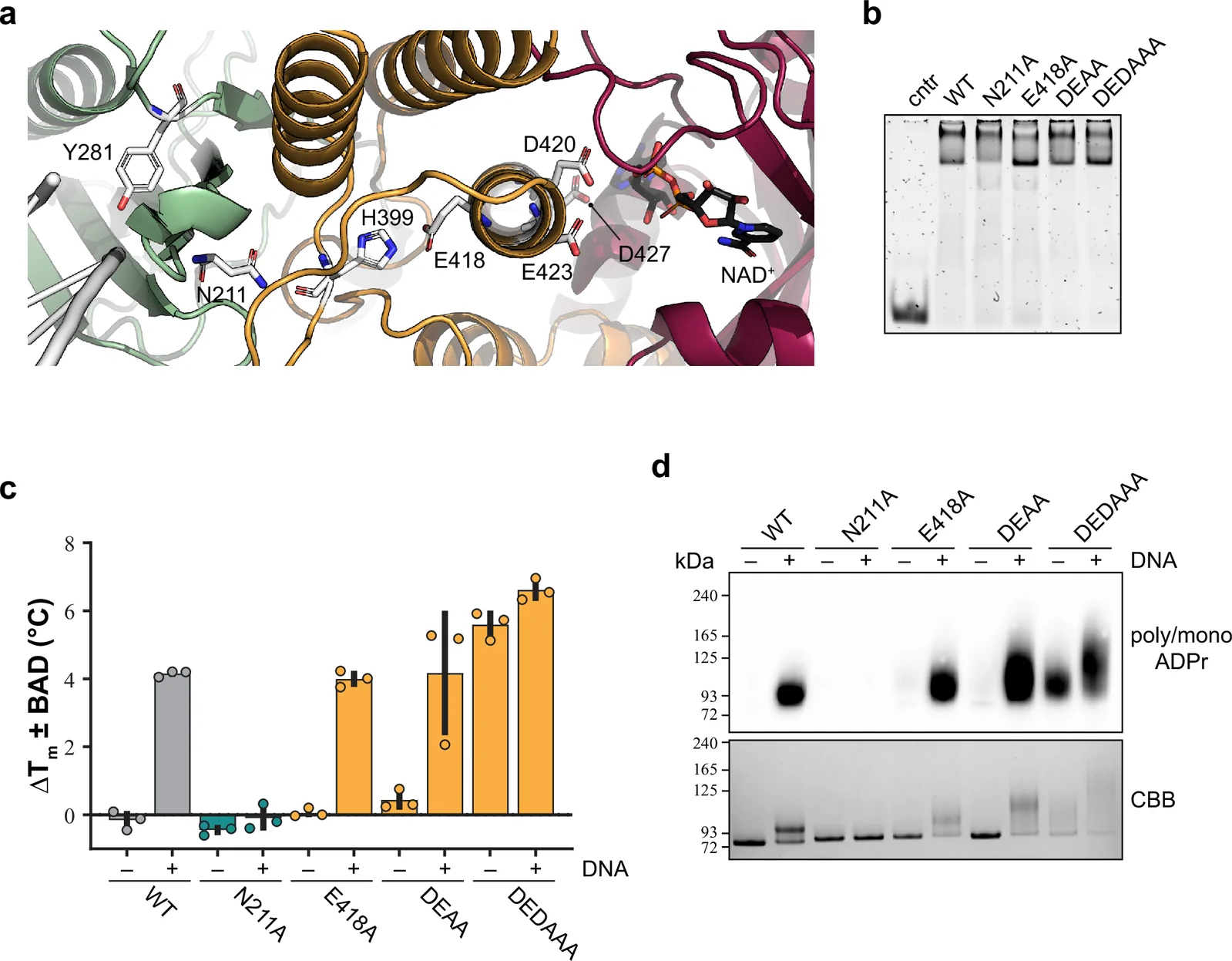
Allosteric regulation of *Af*-PARP1 activity. **a** Ribbon-liquorice representation of the WGR-HD-ART interaction surfaces of *Af*-PARP1. The WGR domain is given in green, the HD domain in yellow and the ART domain in red. The AF3 modeled NAD^+^ (black) and DNA derived from a previously solved *h*PARP2 structure (PDB 6X0L^25^) are given for orientation. **b** EMSA assay assessing the impact of WGR and HD domain mutations on the ability of *Af*-PARP1 to interact with 5′-phosphorylated nicked DNA (DNA-1). Shown is a representative result of two independent experiments. Uncropped images are available in the Source Data file. **c** DSF experiment using full-length *Af*-PARP1 WT or mutants (4 μM) in the presence or absence of DNA-1 (4 μM) and BAD (1 mM). The Δ*T*_m_ is calculated by subtracting the *T*_m_ obtained in the absence of BAD from the *T*_m_ obtained in the presence of BAD. Experiments were conducted in biological triplicates, and the underlying *T*_m_ values are given in Supplementary Fig. 6b, c. Data are presented as mean values ± SD. **d** Auto(ADP-ribosylation) assay determining the impact of WGR and HD domain mutants on *Af*-PARP1 activity in the presence or absence of 5′-phosphorylated nicked DNA (DNA-1). Shown is a representative result of two independent experiments. Uncropped images are available in the Source Data file.

In *h*PARP1/2, DNA-dependent activation is regulated via allosteric autoinhibition exerted through insertion of residues from the αF helix of the HD domain into the active site^41,56,57^. The autoinhibition is relieved through DNA binding to the WGR domain, which triggers conformational changes in the HD domain, moving the inhibitory residues out of the active site and allowing NAD^+^ binding. To elucidate the molecular details of this process for *Af*-PARP1, we performed sequential and structural analysis of the WGR:HD and HD:ART interaction surfaces (Supplementary Figs. 2, 6a, and Supplementary Table 3). We found that Asn211, which forms a key contact between the WGR and HD domains and acts as an initial relay point for signal propagation, is absolutely conserved across all DNA damage response PARPs (Fig. 3a and Supplementary Fig. 5a). Similarly, residues involved in steric occlusion of the active site are highly conserved with Asp423 and Glu427 corresponding to Asp322 and Glu326 identified in *h*PARP2 (Supplementary Figs. 2 and 6a)^56,57^. The negative charge at the position isostructural to Glu427 is absolutely conserved, while it is limited to fungal, animal and plant PARP1/2 at position Asp423. In addition, we identified Glu420, which is more commonly found in animal PARP3 and 4, as well as the fungal-specific Asp418, which replaces a highly conserved lysine residue (Lys317 in *h*PARP2) found across animal and plant PARPs.

To access the impact of these residues on *Af*-PARP1 activation, we performed differential scanning fluorimetry (DSF), DNA binding and PARylation assays. In the DSF assays, the non-hydrolysable NAD^+^ analogue benzamide adenine dinucleotide (BAD) was used to monitor engagement with the active site. Consistent with observations from *h*PARP1, DNA binding to *Af*-PARP1 was not impaired by any of the mutants, but resulted in a *T*_m_ decrease, reflecting the destabilisation of the HD domain upon DNA engagement, while BAD failed to bind in the absence of DNA (Fig. 3b, c and Supplementary Fig. 6b, c). However, BAD bound strongly to *Af*-PARP1 in the presence of DNA (Fig. 3c). Alanine substitution of Asn211 abolished BAD binding (Fig. 3c) as well as the ability to automodify (Fig. 3d) even in the presence of DNA, while DNA binding remained unaffected (Fig. 3b). Next, we probed the conserved residues Asp420, Glu423, and Asp427 predicted to exert steric blocking. While a double mutation D420A/E423A (DEAA) led only to a negligible increase in PAR generation in the absence of DNA (Fig. 3c, d), we observed an apparent increase in chain length upon DNA-mediated activation (Fig. 3c). In contrast, additional mutation of Asp427, forming the D420A/E423A/D427A (DEDAAA) triple mutant, reduced autoinhibition, with BAD binding and PARylation activity comparable to WT in the absence of DNA (Fig. 3c, d). Addition of DNA lead to a mild increase in BAD binding and stimulated formation of longer PAR chains, as indicated by increased smearing in PAR immunoblots (Fig. 3c, d). These observations suggest that the length of the formed PAR chain in *Af*-PARP1 is not due to an intrinsic limitation of its PARylation activity, but rather limited via the allosteric autoinhibition mechanism, most likely triggered by dissociation from the DNA. Lastly, E418A showed BAD binding and PARylation activity comparable to WT (Fig. 3c, d), indicating that it plays no role in the DNA-dependent autoinhibition relief. Interestingly, E418A showed reduced thermal stability, with a *T*_m_ of 37.5 ± 0.15 °C compared to 43.5 ± 0.51 °C for WT (Supplementary Fig. 6b, c), indicating a structural role in stabilisation of the HD domain.

### The BRCT domain of *Af*-PARP1 supports DNA binding

**Fig. 4:**
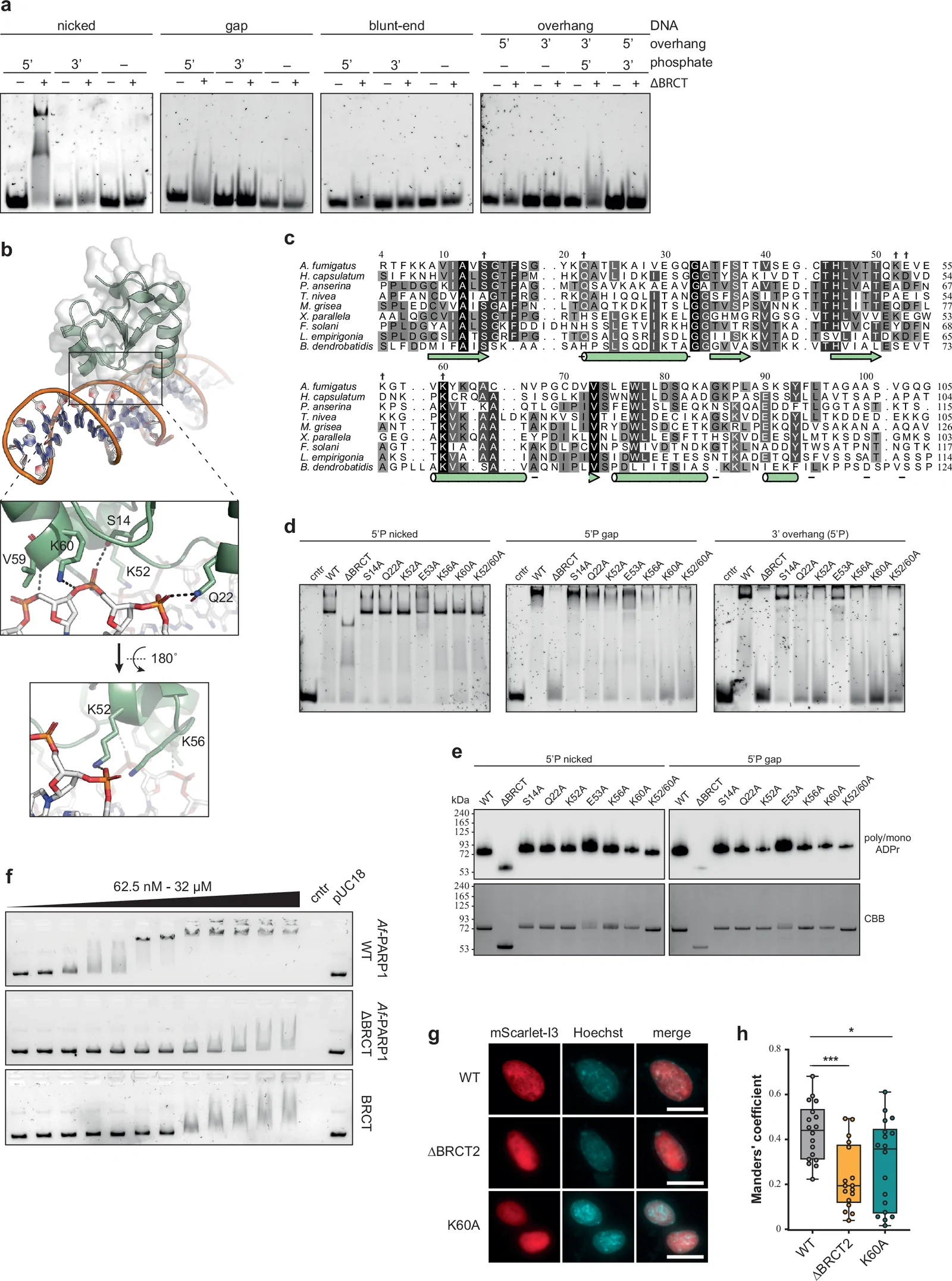
The BRCT domain of *Af*-PARP1 participates in DNA damage recognition. **a** EMSA assay assessing DNA damage binding specificity of *Af*-PARP1 ΔBRCT. Shown is a representative result of two independent experiments. Uncropped images are available in the Source Data file. **b** Binding interface of the BRCT domain (green) in complex with nicked DNA (white) within the AF3 model (Supplementary Fig. 7a). Key residues identified in participating in the interaction are highlighted in the lower panels. **c** Multiple sequence alignment of fungal BRCT domains. Labels above the alignment: residues contributing to the predicted DNA interaction surface (dei [ϯ]) and indexes indicating *Af*-PARP1 residue position; below the alignment: AF3-derived secondary structure. **d** EMSA assay assessing the ability of *Af*-PARP1 WT and mutants to bind 5′-phosphorylated nicked (DNA-1), gap (DNA-2), or 3′ overhang (DNA-9) DNA. Shown is a representative result of two independent experiments. The uncropped images are available in the Source Data file. **e** Auto(ADP-ribosylation) assay assessing the ability of BRCT mutants to stimulate *Af*-PARP1 catalytic activity in the presence of different DNA damage mimetics (see (**d**)). Shown is a representative result of two independent experiments. Uncropped images are available in the Source Data file. **f** EMSA assay assessing the ability of *Af*-PARP1 WT, ΔBRCT or BRCT domain to interact with supercoiled DNA. Proteins were titrated (62.5 nM–32 μM) against pUC18 (8 nM). Shown is a representative result of three independent experiments. Uncropped images are available in the Source Data file. **g** Localisation of N-terminally mScarlet-I3-tagged *Af*-PARP1 WT, ΔBRCT2, and K60A in murine 2H11 cells. Nuclei were visualised by Hoechst 33342 staining, and the scale bar represents 20 μM. **h** Shifts of Manders’ colocalisation coefficient (MCC) due to a change in the *Af*-PARP1 BRCT domain. Values represent the fraction of *Af*-PARP1 (red) overlapping with Hoechst 33342 (blue) fluorescence intensity (MCC-M_2_). Box plot represents the interquartile range showing a median line, and whiskers denote minima and maxima of the data range for *n* = 18 cells (WT and K60A) and *n* = 17 cells (ΔBRCT2). Statistical differences were calculated using an unpaired, two-sided Welch’s t-test with *p* = 0.0155 (*) and *p* = 0.0001 (***).

BRCT domains are widespread amongst proteins involved in DNA replication, repair and cell cycle regulation. They mediate phospho-peptide and phosphorylation-independent protein interactions as well as nucleic acid and ADP-ribosylation recognition^58,59^. Recent studies have implicated the BRCT domain of *h*PARP1 in facilitating interactions with intact DNA, thereby assisting in the diffusion-limited ‘monkey-bar’ mechanism, which allows efficient scanning of the chromatin for DNA damage sites^60,61^. However, the *h*PARP1 BRCT remains mobile even upon formation of the multi-domain DNA damage recognition and activation assembly^34,62^, but the extent of this functional specialisation needs further exploration. Given that the BRCT domain is a distinguishing feature of *Ascomycota* and basal-lineage PARPs relative to other 5′ phosphate-dependent DDR PARPs, we sought to investigate its potential contribution to *Af*-PARP1 function. EMSA assays using a BRCT-truncated *Af*-PARP1 (*Af*-PARP1 ΔBRCT) and our DNA damage mimetic library revealed a markedly restricted binding profile relative to WT (Figs. 2b and 4a). *Af*-PARP1 ΔBRCT displayed clear gel shifts only in response to 5′-phosphorylated nicked DNA (DNA-1), with no discernible binding to gap, overhang or blunt-end DNA substrates. To assess whether the BRCT domain co-recognises the DNA damage or facilitates DNA binding via recognition of intact DNA close to the damage site, we modelled the *Af*-PARP1:DNA-1 complex using AlphaFold 3. The model showed an interaction of the WGR domain with the DNA damage site that closely mimics interactions observed in the *h*PARP2:DNA structure (r.m.s.d. 0.603 Å over 81 C^α^; Supplementary Figs. 3f and 7a)^42^. Notably, the BRCT domain was also predicted to interact with the DNA but is placed on the opposite side of the nick interacting with undamaged DNA and forming no contacts with the WGR domain (Supplementary Fig. 7a). Placement of the BRCT showed model-to-model variation in line with a base-independent backbone interaction (Supplementary Fig. 7b). The predicted BRCT:DNA interaction surface is consistent between models with Ser14, Gln22, Lys52, Glu53, Lys56, and Lys60 identified as primary interaction residues (Fig. 4b). Sequential comparison showed low conservation between different PARP-associated BRCT domains, but a partial conservation of the predicted interaction surface amongst fungi (Fig. 4c, Supplementary Fig. 7c, d, Supplementary Tables 3 and 5). While the overall fold is conserved, structural alignment of the modelled *Af*-PARP1 BRCT domain with its mammalian homologues *h*PARP1 (r.m.s.d. 1.09 Å over 53 C^α^) and *h*PARP4 (r.m.s.d. 2.469 Å over 29 C^α^) revealed some alteration in secondary structure usage (Supplementary Fig. 7c, e). Together, this suggests that the mode of DNA interaction has diverged between fungal and mammalian PARP BRCT homologues. Mutational analysis of the functional relevance of the key residues identified by alanine substitutions (Fig. 4b, c and Supplementary Fig. 7c) showed that all the mutants retained binding to 5′-phosphorylated nicked DNA (DNA-1; Fig. 4d). By contrast, *Af*-PARP1 Q22A, K52A, K56A, K60A, and K52/60A showed reduced affinity for 5′-phosphorylated gap and 3′-overhang DNA (DNA-2 and -9), while the ability of S14A and E53A to bind these structures was only mildly impaired (Fig. 4d). The reduction in binding of the lysine mutations was accompanied by reduced *Af*-PARP1 activation, while E53A increased the activation (Fig. 4e). Together these observations suggest that the BRCT domain of fungal PARPs plays a crucial role in the recognition of gap and overhang DNA damage structures. Moreover, the interaction is primarily mediated by lysine residues, which contribute to an overall positive surface potential at the DNA binding interface (Supplementary Fig. 7f).

Titration of the *Af*-PARP1 WT against a fixed concentration (8 nM) of intact plasmid DNA resulted in progressive upward shifts of DNA in EMSA assays, with saturation achieved at protein concentration of 2 μM, indicative of efficient plasmid coating by *Af*-PARP1 (Fig. 4f). Importantly, these shifts were not limited to the minor fraction of nicked plasmid present but reflected binding across the intact plasmid. In contrast, these shifts were severely diminished with the BRCT truncated variant, and no saturation was achieved across the protein concentration tested (Fig. 4f). The BRCT domain alone induced a shift at lower concentrations and was more pronounced than *Af*-PARP1 ΔBRCT indicative of a stronger interaction, however, direct comparison is limited due to the size differences of both proteins (Fig. 4f). In contrast, no binding of *Af*-PARP1 or its BRCT domain to PAR was detected (Supplementary Fig. 7g). To determine whether the BRCT domain of *Af*-PARP1 contributes to chromatin association within the cellular context, we utilised murine 2H11 cells as a model. While WT *Af*-PARP1 showed clear nuclear localisation, ΔBRCT was primarily localised to the cytoplasm (Fig. 4g and Supplementary Fig. 7h, i). Analysis of the protein sequence identified a potential nuclear localisation signal with a KRKL minimal consensus sequence within the linker region between the BRCT and WGR domains (Supplementary Fig. 7h and Supplementary Table 6). Mutation of this motif (K133N/R134Q/K135N; χNLS) in the WT context led to a similar cytoplasmic localisation (Supplementary Fig. 7i). Therefore, we constructed ΔBRCT2, which removes only the BRCT domain, but leaves the linker unchanged. *Af*-PARP1 ΔBRCT2 showed a WT-like subcellular localisation pattern (Fig. 4g and Supplementary Fig. 7i). Close examination of the sub-nuclear localisation showed that *Af*-PARP1 WT, but not ΔBRCT2 or K60A, appeared to co-localise with Hoechst-stained chromatin structures (Fig. 4g). Co-localisation analysis of high-resolution nuclear volumes showed that both ΔBRCT2 and K60A has a significantly reduced chromatin association, albeit the latter to a lesser extent (Fig. 4h). This supports the idea that the BRCT domain plays a role in maintaining *Af*-PARP1 in proximity to chromatin.

### *Af*-PARP1 forms a multidomain assembly upon DNA damage recognition

**Fig. 5:**
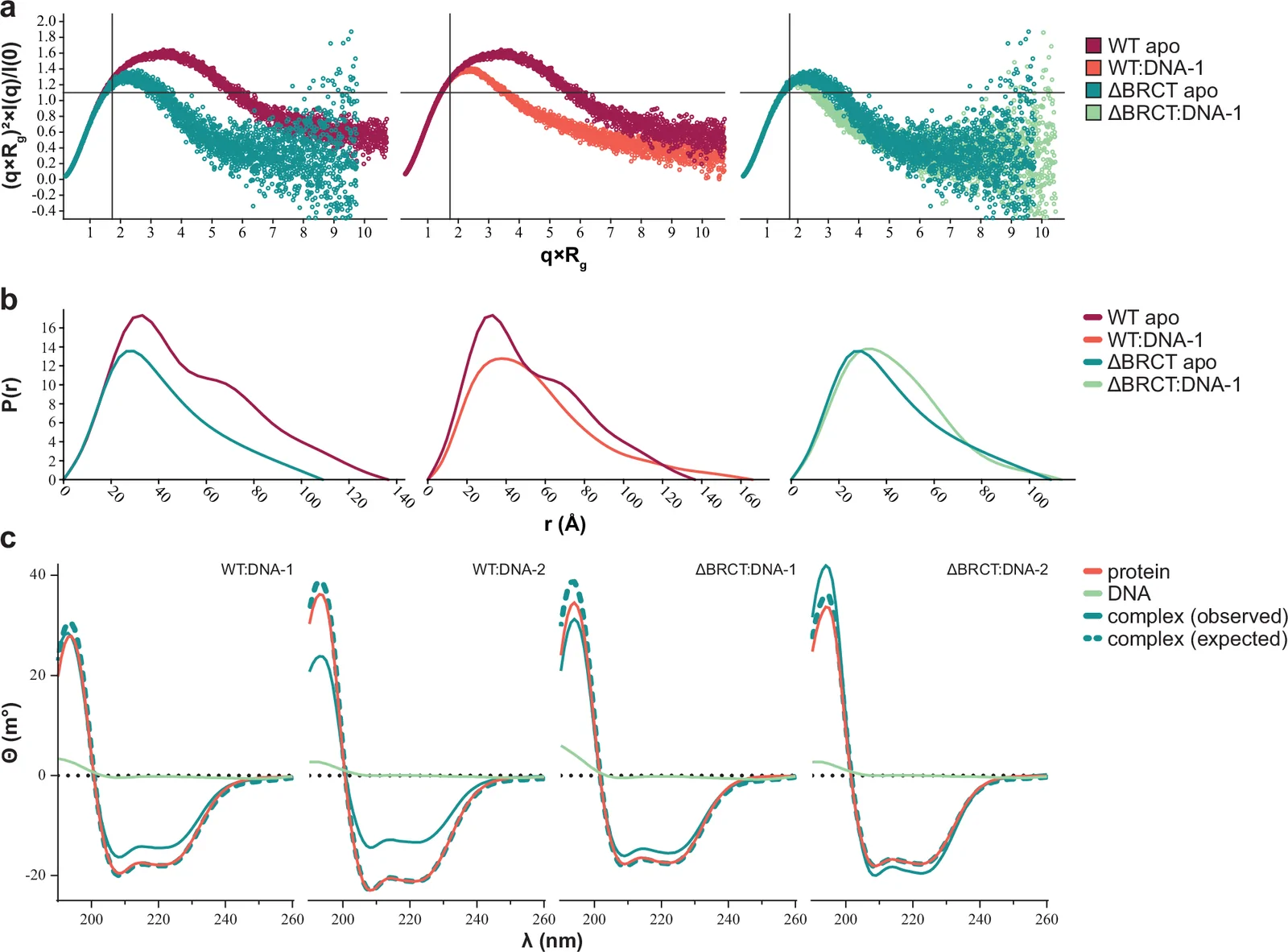
SAXS and CD analysis suggest *Af*-PARP1 compaction upon DNA binding. **a** Normalised Kratky plots of *Af*-PARP1 WT and ΔBRCT in their apo and DNA-1 bound forms. **b** *P*(*r*) distribution plots of *Af*-PARP1 WT and ΔBRCT in their apo and DNA-1 bound forms. All SAXS parameters and analysis outcomes are summarised in Table 1. **c** CD far-UV spectra of *Af*-PARP1 WT and ΔBRCT in the presence and absence of DNA-1 or -2. Expected complex spectra (summation of protein and DNA apo spectra) are shown as a dotted line. Indicative α-helical features are the double minimum at 208 nm (*π* → π*) and 222 nm (*n* → π*), as well as a stronger maximum at 191–193 nm (*π* → π*)^109^.

To better understand the role of the BRCT domain in facilitating DNA damage binding, we performed SAXS experiments utilising *Af*-PARP1 WT and ΔBRCT as well as their complexes with the 5′-phosphorylated nicked DNA damage mimetic (DNA-1; Fig. 5a, b, Table 1 and Supplementary Fig. 8a, b). Together, the SAXS-derived parameters (*R*_g_, *D*_max_, *M*_w_, and porod volume) suggest that both proteins and their complexes are monomeric in solution, while the Kratky analysis of the apo proteins is consistent with overall structured proteins. We observed higher flexibility of *Af*-PARP1 WT, relative to ΔBRCT, due to the BRCT domain and linker region present in the WT construct (Fig. 5a). This is further supported by the bimodal *P*(*r*) function profile of *Af*-PARP1 WT, which is absent in *Af*-PARP1 ΔBRCT (Fig. 5b). In contrast, the *Af*-PARP1 WT:DNA-1 complex revealed a more rigid compact conformation in the Kratky plots as well as single peak *P*(*r*) distributions (Fig. 5a, b). These observations suggest that, upon DNA binding, the BRCT domain transitions from behaving as an independent domain to forming an integral part of the multidomain WGR-HD-ART assembly. This is further supported by the smaller decrease in flexibility of *Af*-PARP ΔBRCT upon binding to DNA-1 (Fig. 5a), which suggests only mild compaction of the WGR-HD-ART domains upon DNA binding. This behaviour of the BRCT domain contrasts with that of *h*PARP1, in which the BRCT domain does not appear to undergo a similar degree of structural compaction upon DNA association^56,63^. In addition to the domain rearrangements observed in our SAXS data, the interaction of *Af*-PARP1 with DNA also has more subtle effects on the proteins secondary structure as assessed by CD measurements (Fig. 5c and Supplementary Fig. 8c). The observed CD spectra of the mixtures of *Af*-PARP1 WT with either DNA-1 or DNA-2 being different than the calculated spectra of the sum of the individual components were qualitatively indicative of the formation of protein:DNA complexes. These complexes display a reduction in helical content relative to the apo form, with a comparable but less pronounced effect observed for the ΔBRCT:DNA-1 complex (Fig. 5c). In contrast, measurement of the ΔBRCT:DNA-2 mixture showed a slightly increased α-helicity (Fig. 5c). Qualitatively, the formation of the protein:DNA complexes was also identified in the near-UV region dominated by the coupling of the DNA bases. In this region, the observed positive CD bands at about 275 nm of the protein-DNA mixtures were larger than what was calculated from the sum of the individual proteins and DNA.

**Table 1:** SAXS sample, data collection and analysis details

(a) Sample details																
Organism	*A. fumigatus*															
Source	*E. coli* (Rosetta (DE3) recombinant expression)															
Description of complex	*Af*-PARP1:DNA															
Scattering particle composition																
Sample	Component 1				Component 2				Complex 1				Complex 2			
Protein	*Af*-PARP1 WT				*Af*-PARP1 ΔBRCT				*Af*-PARP1 WT				*Af*-PARP1 ΔBRCT			
DNA									DNA-1				DNA-1			
Sample environment/configuration																
Solvent composition	20 mM HEPES (pH 7.5), 150 mM NaCl, 2 mM TCEP, 2.5% (v/v) glycerol															
Sample temperature (K)	288															
In-beam sample cell	1.5 mm quartz capillary, sample flow at 1 μL/s															
Size exclusion chromatography (SEC-SAXS)																
	Component 1				Component 2				Complex 1				Complex 2			
Sample injection [μM]	100				50				50/60^a^				50/60^a^			
Sample injection (V)	45 μL				45 μL				45 μL				45 μL			
SEC column type	Superdex 200 Increase 3.2/300 (Cytiva)															
SEC flow rate (mL/min)	0.075				0.075				0.075				0.075			
**(b) SAS data collection**																
				SAXS												
Data acquisition/reduction software				General data acquisition (GDA), DAWN science (Diamond LightSource, UK)												
SAXS beamline				B21 high-throughput beamline (Diamond Light Source, UK)												
Measured *q*-range (*q*_min_–*q*_max_; Å^−1^)				0.0045–0.34												
Method for scaling intensities				Scaled to absolute units (cm^−1^) relative to water												
Exposure time (s)/No. of exposures				3/600				3/630				3/600				3/600
SEC-SAS frames used for averaging				9				16				26				13
**(c) SAS-derived structural parameters**																
Methods/software	Scatter (biosis)															
Guinier analysis																
	Component 1				Component 2				Complex 1				Complex 2			
* I*(0) ± *σ* (cm^−1^)	0.0901 ± 1.3 × 10^−4^				0.0217 ± 4.3 × 10^−5^				0.0672 ± 8.3 × 10^−5^				0.0267 ± 4.7 × 10^−5^			
* R*_g_ ± *σ* (Å)	44.32 ± 0.57				32.19 ± 0.39				43.52 ± 0.40				33.82 ± 0.36			
min <*qR*_g_ < max limit	0.263–1.1128				0.2083–1.2787				0.3661–1.1949				0.2316– 1.2859			
*P*(*r*) analysis																
			Component 1				Component 2				Complex 1				Complex 2	
*I*(0) ± *σ* (cm^−1^)			7.19 × 10^−2^				2.15 × 10^−2^				6.34 × 10^−2^				2.69 × 10^−2^	
*R*_g_ (Å)			41.64				33.04				44				33.43	
*D*_max_ (Å)			136.5				109				166				113.5	
*q*-range (Å^−1^)			0.006–0.161				0.0065–0.2015				0.0084– 0.204				0.0068– 0.1935	
*P*(*r*) fit assessment (score)			1.257				1.32				2.93				1.96	
**(d) Scattering particle size**																
Methods/software	ProtParam (Expasy)^105^, Scatter (Bioisis)															
Molecular weight (*M*_w_) estimates																
		Component 1				Component 2				Complex 1				Complex 2		
* M*_w_ from sequence (kDa)		74.3				57.8				87.9				72		
Porod volume (Å^3^)		135,117				110,600				189,625				150,800		
*M*_w_ porod (kDa)		79.5				65.06				111.5				88.71		
**(e) Data and model deposition**																
	Component 1				Component 2				Complex 1				Complex 2			
SASBDB IDs	SASDXE9				SASDXF9				SASDXG9				SASDXH9			

^a^DNA-1 was used in 1.2-fold molar excess.

Binding interactions determined by EMSA titration of the protein into the DNA revealed that WT and ΔBRCT form complexes with DNA-1 of apparent similar strength, while the interaction of ΔBRCT with DNA-2 is impaired (Supplementary Fig. 8d). Given the observed changes in α-helicity, binding appears to induce conformational changes, and the inability of DNA-2 to induce these changes in the ΔBRCT mutant might correlate with lower activation or reduced catalytic activity.

### The ability of the BRCT domain to bind DNA is evolutionary conserved

**Fig. 6:**
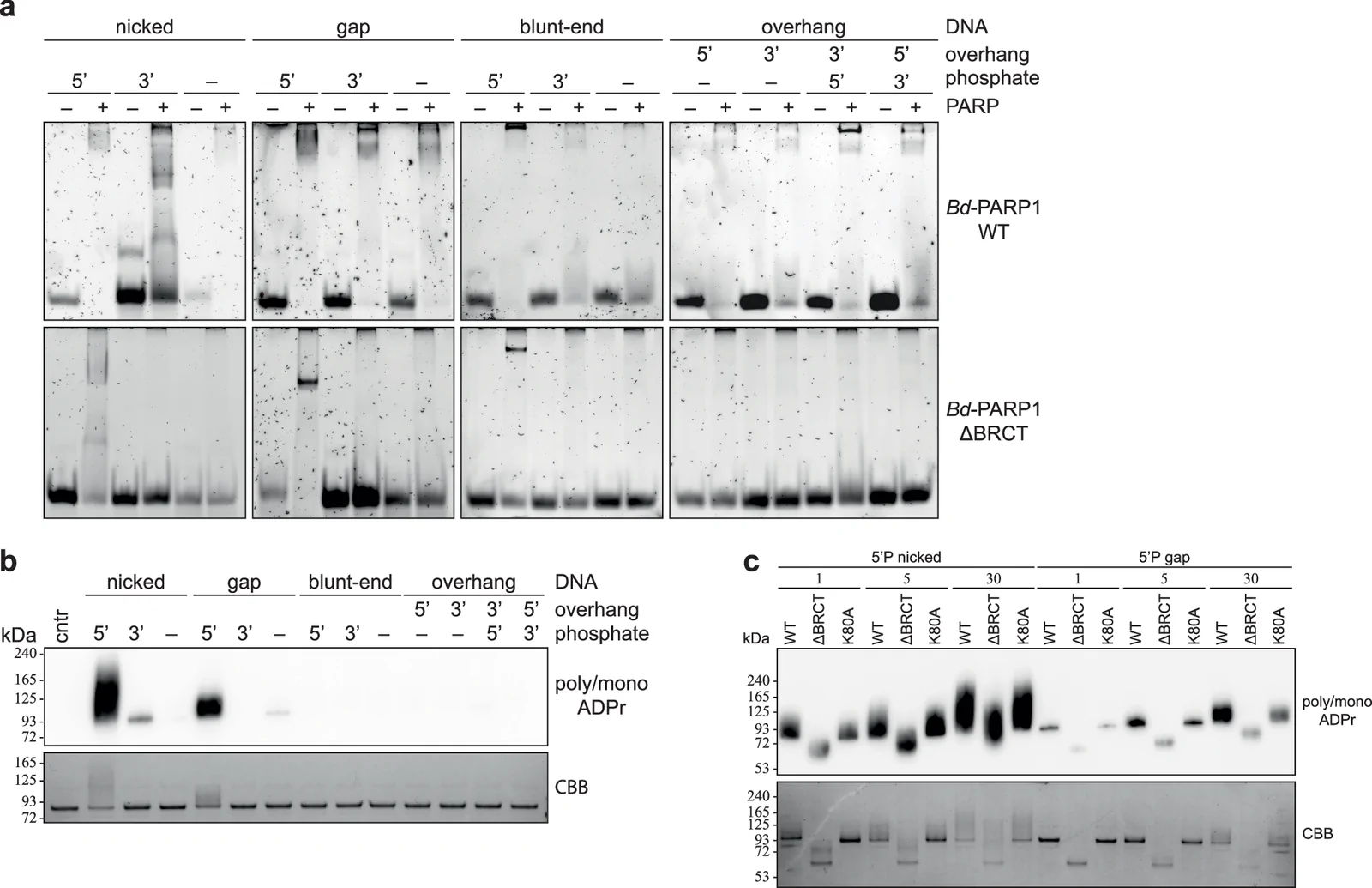
The ability of the BRCT domain to bind DNA is conserved amongst *Fungi.* **a** EMSA assay assessing the DNA damage binding specificity of *Bd*-PARP1 WT and ΔBRCT. *Bd* PARPs (5 μM) were incubated with DNA mimetic (1 μM) for 1 h, separated via native PAGE and DNA visualised with SYBR safe staining. Shown is a representative result of two independent experiments. Uncropped images are available in the Source Data file. **b** Assessment of *Bd*-PARP1 WT catalytic activity exposed to different DNA damage mimetics. *Bd* PARPs (0.5 μM) were incubated with DNA (0.6 μM) and NAD^+^ (500 μM), and the formation of PAR was detected using immunoblot. Shown is a representative result of two independent experiments. Uncropped images are available in the Source Data file. **c** Assessment of the ability of BRCT mutants to stimulate *Bd*-PARP1 catalytic activity in the presence of 5′-phosphorylated nicked (DNA-1) or gap (DNA-2) DNA. *Bd* PARPs (0.5 μM) were incubated with DNA (0.6 μM) and NAD^+^ (500 μM) for the indicated timepoints, and formation of PAR was detected using immunoblot. Shown is a representative result of two independent experiments. Uncropped images are available in the Source Data file.

To evaluate whether the functional role of the BRCT domain is conserved across fungal PARP proteins, we examined the basal-lineage orthologous protein from the amphibian chytrid fungus *Batrachochytrium dendrobatidis* (*Bd*-PARP1). The DNA damage mimetic binding profile of *Bd*-PARP1 WT closely resembles that of *Af*-PARP1 WT (Figs. 2c and 6a), while *Bd*-PARP1 ΔBRCT exhibits markedly reduced binding to gap-, blunt-end-, and overhang-type DNA damage structures (Fig. 6a). The catalytic activity of *Bd*-PARP1 was primarily stimulated by 5′-phosphorylated nicked and gap structures (DNA-1 and -2; Fig. 6b), which is similar to our observation for *Af*-PARP1 (Fig. 6b). However, in contrast to *Af*-PARP1, we observed no activation in the presence of overhang structures. Deletion of the BRCT domain or disruption of the DNA interaction surface via the K80A (equivalent to K60A in *Af*-PARP1; Fig. 4c, d) substantially reduced enzyme activation in the presence of 5′P gap DNA (DNA-2) compared to *Bd*-PARP1 WT, while stimulation by 5′P nicked DNA (DNA-1) remained comparable between the two proteins (Fig. 6c). These results indicate that BRCT domain in fungal PARPs selectively promotes recognition and catalytic activation on gap DNA substrates.

### The BRCT domain influences protein activity

**Fig. 7:**
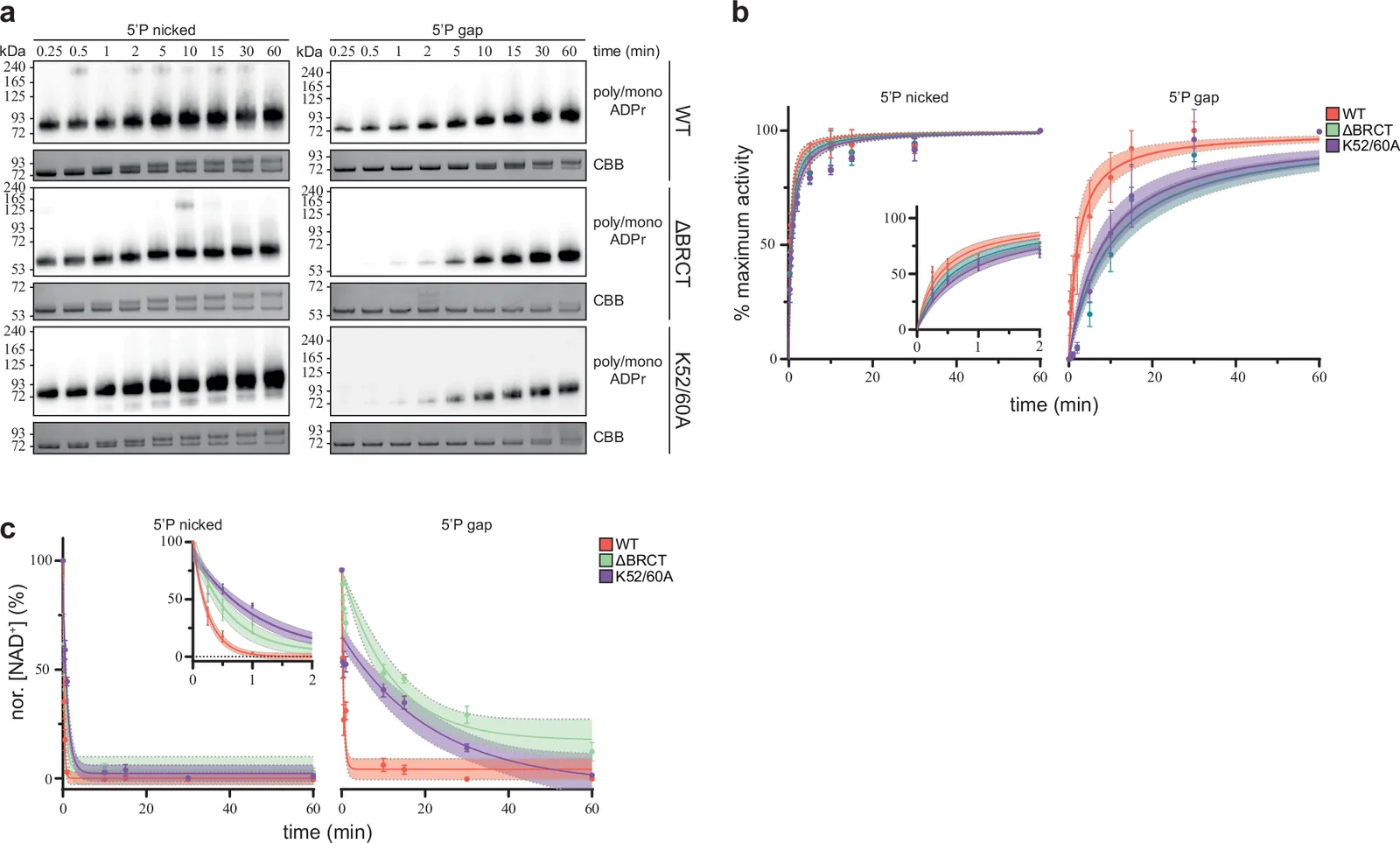
The BRCT domain influences the catalytic activity of fungal PARPs. **a** Kinetics of *Af*-PARP1 WT, ΔBRCT and K52/60A were assessed by time-dependent PAR formation. PARP protein (0.5 μM) was incubated with DNA (0.6 μM) and NAD^+^ (500 μM). Reactions were stopped at defined timepoints by the addition of LDS sample buffer and analysed by immunoblot. Shown is a representative result of three independent experiments. Uncropped images are available in the Source Data file. **b** Quantification of kinetic immunoblot data (representative images shown in (**a**)). Data were normalised to the final timepoint (60 min) and fitted using the Michaelis–Menten model in Prism (GraphPad). Data represent mean ± SD of three biological replicates with best fits as solid lines and 95% confidence intervals (shaded regions). **c** NAD^+^-depletion due to *Af*-PARP1 WT, ΔBRCT, or K52/60A activity was measured in the presence of DNA-1 or -2. Reactions were allowed to proceed for indicated timepoints, stopped using PARPi (Supplementary Fig. 3), and analysed using the NAD/NADH-Glo™ assay (Promega). Data were normalised to control reaction in the absence of PARP and fitted using a one-phase decay model in Prism (GraphPad). Data represent mean ± SD (*n* = 3 biological replicates measured in technical triplicates) with best fits as solid lines and 95% confidence intervals (shaded regions).

Next, we set out to determine whether the DNA-binding ability of the BRCT domain modulates the catalytic activity of *Af*-PARP1. Therefore, we performed time-course assays to quantify the activity of *Af*-PARP1 WT and *Af*-PARP1 ΔBRCT in response to 5′-phosphorylated nicked and gap DNA structures (DNA-1 and DNA-2, respectively) and assessed both product generation (extent of auto-PARylation; carried out in the presence of 500 μM NAD^+^) and substrate consumption (NAD^+^ depletion; carried out using 10 μM NAD^+^). PAR production was quantified by immunoblot (Fig. 7a, b), and signals were normalised to the final time point within each dataset to compensate for potential differences in modification site availability between the protein variants. The presence of 5′-phosphorylated nicked DNA led to strong activation of both *Af*-PARP1 WT and ΔBRCT, reaching half maximal modification at 0.37 min (95% CI: 0.283–0.479) and 0.57 min (95% CI: 0.44–0.746), respectively (Fig. 7a, b). In contrast, *Af*-PARP1 ΔBRCT showed markedly reduced activation compared to WT in the presence of 5′-phosphorylated gap DNA, with half maximal modification reached at 9.87 min (95% CI: 7.78–12.49) compared to 2.13 min (95% CI: 1.38–3.24). For substrate consumption assays, ADP-ribosylation reactions were allowed to proceed for indicated times, stopped by addition of PARPi (Supplementary Fig. 9), and the remaining NAD^+^ quantified (Fig. 7c). The data followed a similar trend with 50% NAD^+^ consumption reached in 0.179 min (95% CI: 0.151–0.212; *Af*-PARP1 WT) and 0.41 min (95% CI: 0.289–0.593; *Af*-PARP1 ΔBRCT), respectively, in the presence of 5′-phosphorylated nicked DNA. Whereas in response to 5′-phosphorylated gap DNA, *Af*-PARP1 ΔBRCT had slowed pointedly, with 50% NAD^+^ conversion achieved after 13.87 min (95% CI: 9.43–21.48) compared to 0.339 min (95% CI: 0.249–0.467) with *Af*-PARP1 WT.

We next examined the catalytic response of *Af*-PARP1 K52/60A to 5′-phosphorylated nicked and gap DNA (Fig. 7a, b). Consistent with the binding data, *Af*-PARP1 K52/60A displayed kinetics comparable to WT and ΔBRCT in response to 5′-phosphorylated nicked DNA, with half-maximal modification achieved after 0.76 min (95% CI: 0.615–0.938; versus 0.37 min for WT and 0.57 min for ΔBRCT). In contrast, when stimulated with 5′-phosphorylated gap DNA, the half-time to maximal modification for K52/60A increased to 8.06 min (95% CI: 5.96–10.87), an intermediate value between WT (2.13 min) and ΔBRCT (9.87 min) (Fig. 7a, b). Analysis of NAD^+^ consumption (Fig. 7c) further reinforced this trend: K52/60A mirrored WT and ΔBRCT kinetics on 5′-phosphorylated nicked DNA but displayed severely impaired consumption on 5′-phosphorylated gap DNA, requiring 15.34 min (95% CI: 8.8–31.38) to reach 50% depletion, compared to 0.339 min and 11.62 min for WT and ΔBRCT, respectively. Taken together, these results indicate that the BRCT domain can broaden the range of DNA damage structures recognised by *Af*-PARP1 and promote efficient ADP-ribosylation signalling by stabilising the *Af*-PARP1:DNA complex required for efficient catalytic activation.

## Discussion

ADP-ribosylation is a crucial signalling pathway in the early DDR of animals and plants. In the present study, we provide evidence that DNA damage-dependent ADP-ribosylation signalling is conserved amongst filamentous fungi but possesses distinct molecular features in damage detection and PARP activation.

### Activation of fungal PARPs is influenced by the BRCT domain

It has been shown that in the *Animalia* ADP-ribosylation system, the modification is predominantly established on serine residues in an HPF-dependent manner^16,27,37,64^. Our analysis failed to identify a sequential or structural homologue to HPF1 within the *Fungi* kingdom, thus suggesting that the modification is primarily established on glutamate and aspartate residues. Surprisingly, we found that the BRCT domain of fungal PARPs plays a pivotal role in DNA damage recognition and stimulation of catalytic activity. Despite considerable efforts to elucidate its role in *h*PARP1, the function of the BRCT domain within DNA-dependent PARPs remains poorly understood.

The BRCT domain of *h*PARP1 has been implicated in protein:protein interactions, PAR recognition, as well as binding to intact DNA^58,59^. Proposed functions include facilitating the assembly of the DNA repair machinery, for example, through interaction with XRCC1^65,66^, or promoting DNA scanning via the ‘monkey bar’ mechanism^60,61^. These functions beyond damage recognition and signal initiation are supported by several lines of evidence, including HDX-MS, SAXS, and computational modelling studies, which suggest that the BRCT domain remains relatively isolated in the presence of damaged DNA and is therefore unlikely to participate directly in DNA damage recognition or in the formation of the catalytic multidomain assembly^30,34,56,63,67,68^. Consistent with this model, deletion of the BRCT domain in *h*PARP1 has little effect on its catalytic activity^69^. In contrast, our data indicate that the *Af*-PARP1 BRCT domain promotes interaction with undamaged DNA (Fig. 4f) and enhances binding to various DNA damage structures (Figs. 2b and 4d). Our combined EMSA and CD analyses provide important insights into the catalytic role of the BRCT domain and its influence on DNA binding and structural rearrangements in *Af*-PARP1. EMSA demonstrated that the BRCT domain is critical for efficient catalytic activity, particularly when interacting with gap-containing DNA (DNA-2). While WT *Af*-PARP1 readily forms stable complexes with both DNA-1 and DNA-2 and exhibits robust catalytic activity, the ΔBRCT variant shows markedly reduced activity on DNA-2 despite retaining detectable DNA binding. These findings suggest that the BRCT domain contributes to efficient substrate engagement and/or structural rearrangements required for full catalytic activation (Fig. 4d).

**Fig. 8:**
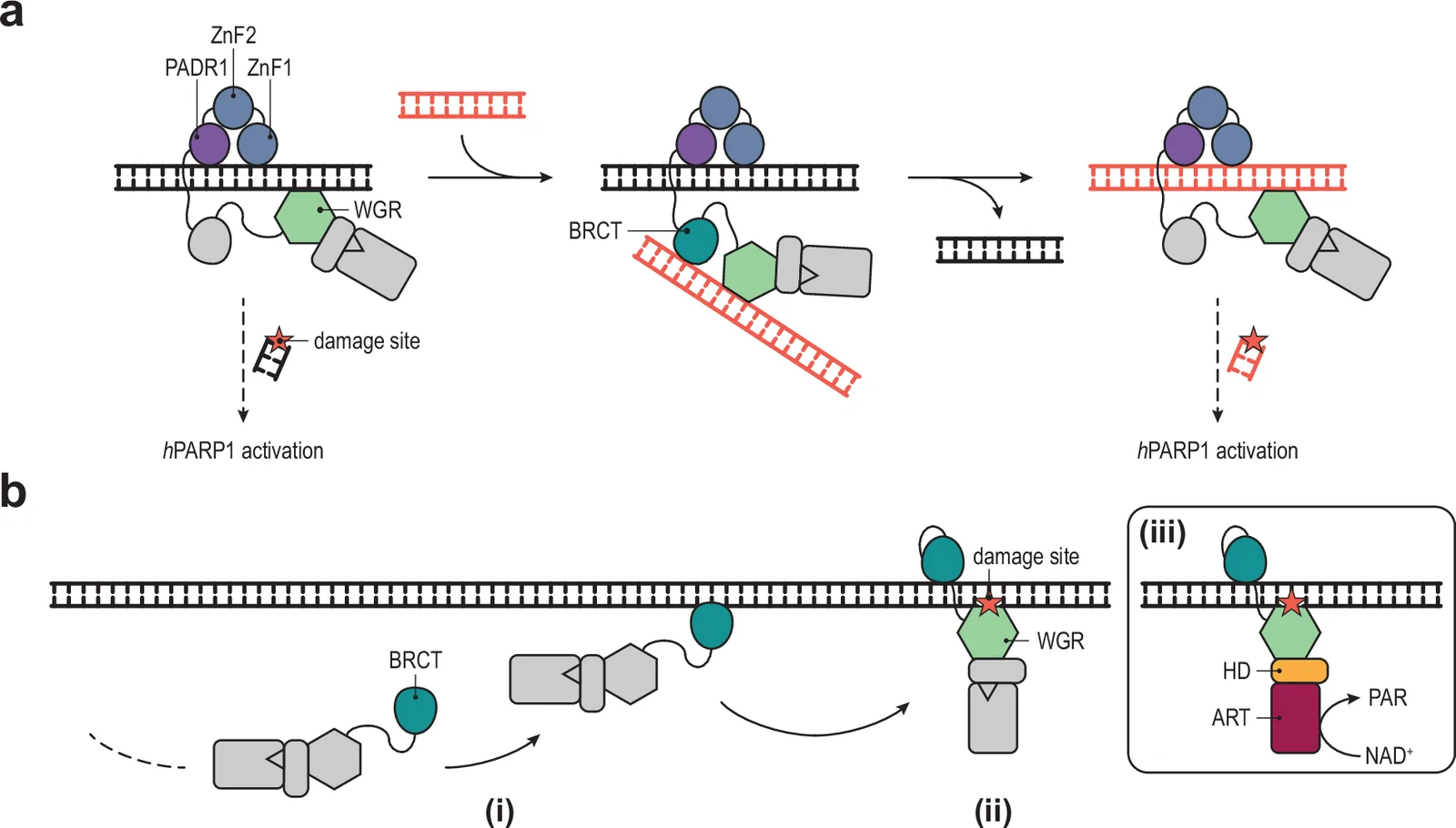
Proposed physiological role of the BRCT domain for *h*PARP1 and fungal PARP activation. **a** The interaction of *h*PARP1 with DNA occurs rapidly and tightly through the WGR, ZnF1, and PADR1 domains so that in the absence of an intrastrand transfer mechanism, re-binding to the same DNA site would be favoured. In the absence of a DNA damage site, intrastrand transfer is initiated by binding of a second strand via the BRCT and WGR domains, followed release of the ‘old’ strand and migration onto the new. The encounter of a DNA damage site promotes conformational changes and leads to *h*PARP1 activation. **b** In the absence of DNA damage, the BRCT domain supports close chromatin association (i) via repeat binding-release events, thus allowing for more efficient chromatin scanning. When binding occurs close to a DNA damage site (ii), the lesion is bound by the WGR domain while the BRCT domain supports the interaction (ii). In turn, formation of this complex leads to activation of the ART domain and generation of a PARylation signal (iii).

Complementary CD measurements revealed that complex formation induces significant conformational changes in *Af*-PARP1. Complexes of WT *Af*-PARP1 with either DNA-1 or DNA-2 display a reduction in α-helical content relative to the apo form, consistent with structural rearrangements necessary for activation (Fig. 5c). This is further supported by the T_m_ decrease observed upon DNA-1 binding in the DSF experiments (Supplementary Fig. 6b, c). In contrast, the ΔBRCT:DNA-1 complex showed a less pronounced loss of α-helicity, suggesting partial engagement but reduced catalytic competence. Strikingly, the ΔBRCT:DNA-2 complex exhibited a slight increase in α-helicity (Fig. 5c), implying that although ΔBRCT can form a low-affinity complex with DNA-2 in solution, it fails to adopt the conformational state required for catalysis. This observation is consistent with EMSA and activity data, which indicate impaired catalytic activity despite detectable DNA binding (Fig. 4a, d and Supplementary Fig. 8d) and highlights the functional importance of the BRCT domain in orchestrating DNA-induced conformational changes required for *Af*-PARP1 activation. The inability of ΔBRCT to transition into a catalytically active state on gap DNA suggests that BRCT acts as a structural facilitator, stabilising the active conformation upon binding to DNA damage sites. This mechanism might enhance the efficiency of DNA repair in *Af*, contributing to genome integrity under stress conditions. Loss of BRCT-mediated regulation could compromise repair fidelity, highlighting its potential as a target for antifungal strategies. Interestingly, in addition to *h*PARP1 the BRCT domain of *h*PARP4 (a vault-associated mono(ADP-ribosyl)transferase^70,71^) was identified as engaging with vault RNA^72,73^. However, our analysis showed little sequence conservation and small changes in secondary structure content between the PARP-associated BRCT domains (Supplementary Fig. 7c–e). These changes alter the regions isostructural to the fungal BRCT DNA interaction surface, hence suggesting a divergence in the mode of interaction with nucleic acids between fungal and mammalian PARPs. While the physiological role of RNA binding by the BRCT domain of *h*PARP4 remains elusive, the interaction between DNA and the *h*PARP1 BRCT, in cooperation with the WGR domain, has been proposed to promote intersegment transfer through the so-called ‘monkey-bar’ mechanism, and enhancing the efficiency of genomic DNA scanning for damage sites (Fig. 8a)^60,61^. In this model, the initial binding occurs via the zinc finger domains of *h*PARP1, while the transfer to a second DNA strand is mediated by the WGR and BRCT domain^60^. Since *Af*-PARP1 lacks the zinc fingers, a monkey bar-type DNA search mechanism appears unlikely for fungal PARPs. Instead, our data demonstrated that the *Af*-PARP1 BRCT domain promotes the interaction of *Af*-PARP1 with both intact DNA and different DNA damage structures in vitro (Figs. 2b and 4a, f) as well as chromatin-association in the murine cell model (Fig. 4g, h and Supplementary Fig. 7i). This suggests that the *Af*-PARP1 BRCT domain may contribute to genome scanning for DNA lesions, a process that occurs on timescales difficult to reconcile with diffusion alone^74^. In addition, given the relative low affinity of the WGR domain for intact DNA (Fig. 4f), it appears unlikely that it is a major contributor to DNA sampling. We therefore propose a model in which the BRCT and WGR domains of fungal PARPs act cooperatively. In this scenario, the BRCT domain would undergo repeated binding/release events, thereby maintaining *Af*-PARP1 in proximity to chromatin. The WGR domain would then sample the DNA for lesions and, upon damage recognition, anchor *Af*-PARP1, facilitate conformational changes required for activation, and promote PARylation signal establishment (Fig. 8b).

### Importance of ADP-ribosyl signal dynamics in the DDR

In the mammalian system, ADP-ribosylation signalling is a highly dynamic process, with signal onset occurring within the first second of genomic injury and turnover occurring in the seconds to minutes range^75,76^. This highly transient nature of the signal is crucial for effective coordination of the early DDR stages. Although the BRCT domain is not strictly required for *Af*-PARP1’s PARylation activity, our data indicates that its ability to interact with DNA greatly influences the dynamic of signal establishment (Fig. 7). This effect is particularly evident on 5′-phosphorylated gap structures, for which both the ΔBRCT and lysine point mutations have a diminished binding affinity (Fig. 4d). While our kinetic data cannot resolve shorter reaction times due to technical limitations, we consistently observed slower reaction kinetics for ΔBRCT and K52/60A at the earliest measurable time points (Fig. 7b, c). These data suggest that the fungal BRCT domain has an integral role not only in damage site recognition but also in signal establishment.

Since *Af*-PARP1 preferentially binds to and is activated by single strand nicks, gaps, and overhang DNA damage structures (Fig. 2b, c), its physiological role may lie in repair pathways similar to those involving *h*PARP1/2, including single strand break (SSB) or base excision repair (BER)^12,77^. In addition, activation by 3′-overhang DNA structures raises the possibility that *Af*-PARP1 may also participate in repair pathways involving DNA-end resection, such as non-homologous end joining (NHEJ) and microhomology-mediated end joining/alternative non-homologous end joining (MMEJ/aNHEJ). Our phylogenetic analyses show no redundancy for DDR-associated PARP in filamentous fungi, providing an opportunity to study fundamental concepts of DNA damage related to ADP-ribosylation signalling in a comparatively simplified system while retaining relevance to the more complex pathways found in animals and plants. Moreover, our *Af*-Δ*parp1* strain (Fig. 1b), as well as previously reported PARP knockouts in several fungal plant pathogens, have shown a substantial reduction in virulence. However, the underlying mechanistic connection to the DDR, if any, remains elusive^29–31^. Future work will need to clarify whether fungal PARPs are genuine virulence factors, play an active role in adaptation to the host environment, or protect the fungus from stresses within the infection niche. The molecular features identified here highlight substantial divergence between fungal and mammalian pathways, supporting the idea that fungal PARPs may represent attractive targets for antifungal therapy.

## Methods

### *B. dendrobatidis* culture, RNA extraction, and cDNA synthesis

*Batrachochytrium dendrobatidis* strain JEL423 zoosporangia and zoospores were cultured in half-strength TGhL broth (0.8% (w/v) tryptone, 0.1% (w/v) gelatine hydrolysate, 0.2% (w/v) lactose) for four days in tissue culture-treated flasks at 18 °C under static conditions.

Total RNA was extracted using the Monarch® Total RNA Miniprep Kit (NEB). Briefly, cells were scraped from flasks, collected by centrifugation (2,500 × *g*, 5 min, 4 °C), washed twice with PBS, and flash-frozen in liquid nitrogen. Pellets were resuspended in DNA/RNA Protection Reagent and disrupted by bead beating in a FastPrep-24 homogenizer (MP Biomedicals) with 0.5 mm zirconia/silica beads (4 cycles of 30 s with 1 min on ice between cycles). Subsequent steps were performed according to the manufacturer’s instructions. RNA concentration was determined using a Qubit 4.0 Fluorometer (Invitrogen, USA), and RNA integrity was assessed with a TapeStation 4200 (Agilent, USA). Total RNA was reverse transcribed using the UltraScript® cDNA Synthesis Kit (PCR Biosystems Ltd.) according to the manufacturer’s instructions and stored at −80 °C until use.

### *A. fumigatus* strain, culture, and transformation

*A. fumigatus* strain AfS77 (akuA::loxP [ATCC46645 background]) was a kind gift from Prof Sven Krappmann (University Hospital Erlangen). *Af* was cultured on malt extract agar (MEA) for 3 days at 37 °C unless stated otherwise. Conidia were harvested in dH_2_O and filter through Miracloth (Millipore).

Construction of the gene replacement cassette and protoplast-mediated transformation was based on earlier described methods^78,79^. Briefly, to generate the *Af*-*parp1* knockout cassette three PCR fragments were amplified: 1000 nt genomic regions up and downstream of the *Af*-*parp1* gene (positions −1001 to −1 and 2311 to 3311 relative to the star codon adenosine, respectively) were amplified from genomic DNA and the Hygromycin resistance gene from the pUC19-Hph plasmid (a gift kind from Michael Bromley [Addgene plasmid # 188741; http://n2t.net/addgene:188741; RRID:Addgene_188741]^80^). Fusion of purified fragments was carried out as described by Zhao and collegues^78^. The resulting KO cassette was gel-purified and stored as aqueous solution at −20 °C until transformation.

For protoplast generation 40 mL malt extract broth (MEB; 17 g/L malt extract, 3 g/L mycological peptone) was inoculated with 2 × 10^8^ conidia and incubated for 16 h at 125 rpm and 30 °C. Hyphae were harvested by filtration, washed twice with MEB and resuspended in 16 mL MEB. 16 mL 2× protoplasting solution (sterile filtered 1.1 M KCl, 0.1 M citric acid adjusted to pH 5.8 with 1.1 M KOH and containing 128 g/L Vinoflow FCE [Novo Nordisk]) was added, and the digestion reaction was carried out at 100 rpm and 30 °C for 2 h. Undigested hyphae were removed by filtration, and the protoplast-containing solution mix with equal volume 0.6 M KCl. Protoplasts were harvested by centrifugation (1800 rpm) and washed thrice with ice-cold 0.6 M KCl (2400 rpm centrifugation). Protoplasts were washed once with ice-cold 0.6 M KCl, 50 mM CaCl_2_, resuspended in ice-cold 0.6 M KCl, 50 mM CaCl_2_ and enumerated using a hemocytometer. One hundred microliters protoplast suspension (1 × 10^7^ protoplasts/mL) were mixed with 10 μL *parp1* KO cassette construct (approx. 1 μg DNA) by vortexing. 50 μL freshly filtered PEG solution (0.6 M KCl, 50 mM CaCl_2_, 10 mM Tris-HCl (pH 7.5), 25% (w/v) PEG 3350) was added and incubated for 25 min in an ice-water bath. One millilitre PEG solution was added, mixed by gentle pipetting, and incubated for 25 min at RT. Mixture was mixed with 50 mL molten and cooled (approx. 40 °C) Edhen’s Minimal Medium for *Aspergillus* (EMMA; Supplementary Note 1) containing 0.6% (w/v) purified agar, 1 M sucrose, and 200 μg/mL hygromycin B (Sigma-Aldrich) and overlayed on petri dishes containing EMMA containing 1.8% (w/v) purified agar, 1 M sucrose, and 200 μg/mL hygromycin B. Plates were incubated overnight right-site up at RT and then inverted and transferred to 37 °C. Hygromycin-sensitive transformants were purified by streaking twice for single colonies on MEA supplemented with 200 μg/mL hygromycin B, and successful deletion of *Af*-*parp1* was verified by spore PCR^78^.

### *L. oleraceus* plant material, RNA extraction, and cDNA synthesis

Seeds of *Lathyrus oleraceus* (formerly *Pisum sativum*) ‘Blauwschokker’ (purchased from Premier Seeds Direct Ltd, Wilton, UK; batch PL530/12/210/L68/A) were germinated, wrapped in a moistened paper towel and sealed in a plastic bag for seven days at RT. Plant tissue excluding remaining seed tissue was collected and snap frozen in liquid nitrogen and stored at −80 °C until RNA extraction.

Total RNA was extracted according to the modified SDS-LiCl protocol by Vennapusa and colleagues^81^. Briefly, frozen tissue was pulverised in a liquid nitrogen-cooled mortar, transferred into a microcentrifuge tube, and resuspended in RNA extraction buffer (100 mM TrisHCl [pH 8], 2.5 M NaCl, 25 mM EDTA, 2.5% (w/v) PVP, 2.5% (v/v) β-mercaptoethanol). Suspension was incubated 5 min at RT before addition of SDS to a final concentration of 2% (w/v) and further incubation for 2 min at RT. Insoluble material was removed by centrifugation (16,000 × *g*, 5 min, 4 °C). Aqueous phase was mixed by vortexing with equal volume of phenol (saturated with 0.1 M citrate buffer [pH 4.2]):chloroform:isoamyl alcohol (25:24:1). Aqueous phase was recovered by centrifugation and washed once with chloroform before addition of LiCl and sodium acetate (pH 4.8) to final concentrations of 2 M and 0.3 M, respectively. RNA was precipitated by incubation at −20 °C for 1 h and collected by centrifugation (16,000 × *g*, 20 min, 4 °C). Pellet was washed once with 2 M LiCl and once with 80% (v/v) ethanol and dried at 45 °C. RNA was dissolved in DCEP-treated dH_2_O, concentration determined by NanoDrop (Thermo Scientific) measurement, and stored at −80 °C until use. Total RNA was reverse transcribed using the UltraScript® cDNA Synthesis Kit (PCR Biosystems Ltd.) according to the manufacturer’s instructions and stored at −80 °C until use.

### Cell culture

Mouse endothelia 2H11 (ATCC CRL-2163) cells were maintained in RPMI 1640+GlutaMax (Gibco) supplemented with 10% foetal bovine serum (FBS) and penicillin-streptomycin (100 U/mL, Gibco) and were cultured in a humidified atmosphere at 37 °C with 5% CO_2_. For imaging, 1 × 10^4^ viable 2H11 cells were seeded into 8-well tissue culture-treated chamber slides (iBidi) 24 hrs before transfection of plasmids using Metafectine (Biontex) according to the manufacturer’s instructions. After 16 hrs, the transfection-containing media was removed, and the cells were washed three times with pre-warmed PBS. Nuclei were stained with 1 μg/mL Hoechst 33342 diluted in Leibovitz’s L-15 media (Gibco) for 30 min at 37 °C, 5% CO_2_. After staining was complete, cells were washed three times with pre-warmed PBS before replacing cells into Leibovitz’s L-15 media. Cells were allowed to recover for 15 min at 37 °C, 5% CO_2_ prior to imaging.

### Virulence assessment of *A. fumigatus* in *Galleria mellonella*

Sixth instar larvae of *G. mellonella* (Livefood UK Ltd, Somerset, UK) were stored at 16 °C before to use. Immediately prior to experimentation, waxworms were assessed for health based on movement, colouration and absence of melanisation, and individuals of similar size were selected.

For survival studies, spores were diluted to 1 × 10^6^ spores/ml in sterile PBS containing 100 µg/ml ampicillin and 50 µg/ml kanamycin. Ten waxworms were used per treatment group, and PBS containing antibiotics alone was used as a no-treatment control. The last left proleg of each waxworm was sterilised with 70% ethanol prior to injection of 10 µl spore suspension (1 × 10^4^ spores). Waxworms were incubated at 37 °C and survival was assessed daily, with death defined by melanisation and lack of movement.

### Plasmid construction

Expression vectors for *h*PARP2 and *h*PARP3 (in pET28b and pDEST17, respectively) were previously described^41^. Protein sequence of *Af*-PARP1 (AFUB_054870) was transformed into coding sequence, and codon optimised for expression in *Escherichia coli* (K12 strain) using the JCat web tool (Supplementary Note 2)^82^. The resulting coding sequence, including HRV3C cleavage site and attB1/2 cloning sites, was gene-synthesised (GeneArt™; Thermo Fisher Scientific) and transferred into pDEST17 via gateway cloning (Life Technology) using pDONR221 as donor vector. The protein coding sequence of *Bd*-PARP1 (BDV3_003859), *Lo*PARP2 (PX314872), and *Lo*PARP3 (PX437880) were amplified from cDNA isolated from strain JAL423 and cultivar ‘Blauwschokker’, respectively, (see above) and transferred into pET9H_3_^83^ via HiFi DNA assembly (New England Biolabs). For mammalian expression, the coding sequence of *Af*-PARP1 was fused N-terminally with JCat-codon optimised V5-mScarlet-I3 (Supplementary Note 3) and introduced into pSecTag 2c (Thermo Fisher Scientific) via HiFi DNA assembly removing the vector-based Igκ secretion signal. RNF146 WWE domain was cloned C-terminally fused to mScarlet-I3 into pSXG^84^, replacing the N-terminal GST-tag and introducing a C-terminal His-tag. All indicated mutations were introduced via PCR-based site-directed mutagenesis using QuickChange Lightning™ (Agilent).

### Protein expression and purification

Human (ADP-ribosyl)hydrolases (PARG, ARH1, ARH3, MacroD1, and TARG1) were a kind gift from Ivan Ahel, and their recombinant production was described before^52,85,86^. Recombinant *Sau*Macro was purified previously in our laboratory^87^.

Recombinant proteins were expressed in Rosetta (DE3) pLysS cells grown in autoinduction Terrific Broth with trace metals (Formedium) supplemented with appropriate antibiotics as well as 10 mM benzamide in the case of *h*PARP2, *h*PARP3, and *Lo*PARP2. Cultures were grown at 37 °C for 7 h before the temperature was lowered to 20 °C for 16 h. Cells were harvested by centrifugation, and pellets were resuspended in lysis buffer (50 mM Tris-HCl [pH 8], 500 mM NaCl, 20 mM imidazole) and stored at −20 °C until use.

#### Batch purification for biochemistry

Proteins were batch purified using bench-top gravity flow columns (BioRad). Clarified cell lysates were incubated for 1 h at 4 °C on a rotating wheel with 400 μL pre-equilibrated (equilibration buffer: 50 mM TrisHCl (pH 8), 500 mM NaCl, 20 mM imidazole) 50% Ni-NTA slurry per 1 L of expression culture before application to a gravity column. Samples were washed twice with equilibration buffer, twice with wash buffer (50 mM TrisHCl [pH 8], 500 mM NaCl, 40 mM imidazole), and proteins were eluted with 50 mM TrisHCl (pH 8), 250 mM NaCl, 500 mM imidazole. Eluted proteins were dialysed for 16 h at 4 °C against 50 mM TrisHCl (pH 8), 200 mM NaCl, 5% (v/v) glycerol, 1 mM DTT. Proteins were aliquoted and stored at −80 °C until use, and their purity was assessed by SDS-PAGE and Coomassie brilliant blue staining.

#### Large-scale purification for purity and CD/SEC-SAXS

Cells were lysed by sonication and lysates clarified by centrifugation (35,000 × *g*, 50 min, 4 °C). *Af*-PARP1 WT and *Af*-PARP1 ΔBRCT were purified using an AKTA Pure FPLC system (Cytiva). Clarified lysates were first loaded onto a HisTrap HP column (Cytiva) and bound protein washed with high-salt buffer wash (25 mM HEPES [pH 8], 1.5 M NaCl, 20 mM imidazole) to remove bound nucleic acids, before elution in 500 mM NaCl, 25 mM HEPES [pH 8] with a stepped gradient of imidazole (80, 150 and 250 mM). His-affinity tags were subsequently removed by incubation HRV3C during dialysis against 25 mM HEPES (pH 8), 500 mM NaCl, 20 mM imidazole for 16 h at 4 °C. Uncleaved protein and protease was removed using a HisTrap HP column (Cytiva) and a GSTrap HP column (Cytiva). Proteins were further purified using isocratic elution size-exclusion chromatography on a HiLoad 16/600 Superdex S200 pg (Cytiva) with 20 mM HEPES (pH 8), 200 mM NaCl, 0.2 mM TCEP (*Af*-PARP1 ΔBRCT) or 10 mM HEPES (pH 7.5), 100 mM NaCl, 1 mM DTT (*Af*-PARP1 WT). Fractions containing target proteins were concentrated and stored at −80 °C until use. *Lo*PARP2 was purified as above, but omitting the His-affinity tag cleavage step and *h*PARP2 and *h*PARP3 were purified as described above with the addition of a Heparin affinity purification step as follows: proteins eluted from the His-affinity column were diluted five-fold with 25 mM TrisHCl (pH 7.5), 100 mM NaCl, 1 mM DTT, and loaded onto HiTrap Heparin (Cytiva) column. Proteins were eluted via a linear salt gradient from 100 to 1000 mM NaCl in 25 mM TrisHCl (pH 7.5) and 1 mM DTT. PARP-containing fractions were dialysed overnight at 4 °C against 25 mM HEPES (pH 8), 100 mM NaCl, 0.4 mM DTT, and further purified using isocratic elution size-exclusion chromatography on a HiLoad 16/600 Superdex 200 pg (Cytiva) with 25 mM HEPES (pH 8), 100 mM NaCl, 0.4 mM DTT. Fractions containing target proteins were concentrated and stored at −80 °C until use.

### Electrophoretic mobility shift assays

Before analysis, DNAs were annealed by heating dissolved DNA to 95 °C for 1 min and then allowed to cool slowly to RT in the annealing machine (while this was switched off). For DNA damage library screening, proteins and DNAs at variable concentrations (given in text) were incubated in 50 mM HEPES (pH 8), 100 mM NaCl, 10% (v/v) glycerol, 10 mM DTT for 45 min at 4 °C. 6x loading dye (30% [w/v] sucrose, 0.015% [w/v] bromophenol blue) was added to a 1× final concentration. 7% (w/v) acrylamide gel was pre-ran in 0.5x TBE for 1 h at 150 V and 4 °C. Subsequently, samples were run in 0.5× TBE at 150 V and 4 °C. For intact DNA EMSA assays, a constant concentration of pUC18^88^ (8 nM; Agilent) was used, and protein was titrated in from 62.5 nM to 32 μM in 20 mM Tris HCl [pH 8], 30 mM NaCl, 1 mM EDTA, 1 mM DTT. TriTrack loading dye (Thermo Scientific) was added to the samples before being ran on 1% (w/v) agarose gel at 80 V for 1 h. Gels were stained in 1× SYBR Safe (Invitrogen) in dH_2_O for 30 min at RT with agitation and documented with ChemiDoc imager (BioRad).

### PARP automodification assays

PARP proteins (0.5 μM) were incubated with DNAs (0.6 μM), annealed as described previously, and NAD^+^ (500 μM) in automodification buffer (50 mM Tris HCl [pH 8], 200 mM NaCl, 1 mM MgCl_2_, 1 mM DTT) for 30 min at 30 °C, unless otherwise stated. Reactions were stopped with LDS sample buffer (Invitrogen) supplemented with 1 mM DTT. Modified proteins were then separated using electrophoresis on NuPAGE 4–12% Bis-Tris Gels (Invitrogen) and transferred to nitrocellulose membranes (BioRad) using a Trans-Blot Turbo (BioRad). Blotted membranes were blocked in PBS buffer supplemented with 0.1% (v/v) Tween 20 and 2.5% (w/v) milk powder (Marvel, Premier Foods plc) for 1 h at room temperature with agitation. Membranes were then incubated with poly/mono-ADP-ribose (clone E6F6A [RRID:AB_2749858] or D9P7Z [RRID:AB_3716623], Cell Signalling Technology), PAR/pADPr (R and D Systems Cat# 4335-MC-100, RRID:AB_3644339), or mono-ADP-Ribose antibody (Bio-Rad Cat# HCA354, RRID:AB_3720089) or with one of the ADP-ribose binding reagents: anti-pan-ADP-ribose (Millipore Cat# MABE1016, RRID:AB_2665466), anti-poly-ADP-ribose (Millipore Cat# MABE1031, RRID:AB_2665467), or anti-mono-ADP-ribose (Millipore Cat# MABE1076, RRID:AB_2665469) overnight at 4 °C. After washing with PBS buffer containing 0.1% (v/v) Tween 20, the membranes were incubated with a horseradish peroxidase-labelled anti-rabbit IgG (Agilent Cat# P0399, RRID:AB_2617141) or anti-mouse IgG (Agilent Cat# P0447, RRID:AB_2617137) for 1 h at RT. Detection was performed with SuperSignal™ West Pico PLUS chemiluminescent substrate (Thermo Scientific), and chemiluminescence was recorded using ChemiDoc imager (BioRad). Quantification of chemiluminescent signal was carried out in Fiji^89^ and analysis in Prism v. 10.6.1 (GraphPad) using the Michaelis–Menten model. *K*_m_ values are interpreted as the time needed to reach half-maximal modification.

### Chemical characterisation of *Af*-PARP1-ADPr conjugates

After ADP-ribosylation as described above, the nature of the *Af*-PARP1-ADP-ribose linkage was determined based on previously described methodology^54,90^. Briefly, an initial *Af*-PARP1 automodification reaction was split and diluted 1:1 with (i) 200 mM MOPS·NH_4_OH (pH 7), (ii) 200 mM HCl, (iii) 200 mM NaOH, (iv) 2 M NH_4_Cl, 200 mM MOPS·NH_4_OH (pH 7), or (v) 2 M NH_2_OH (neutralised to pH 7 with NH_4_OH), 200 mM MOPS·NH_4_OH (pH 7). Reactions (i–iii) were incubated for 1 h, and reactions (iv) and (v) for 4 h at 37 °C. Samples were mixed with NuPAGE LDS sample buffer (Invitrogen) and immediately analysed by immunoblot and CBB staining.

### (ADP-ribosyl)hydrolase characterisation of *Af*-PARP1-ADPr conjugates

Auto-modified *Af*-PARP1 substrates were generated by performing PARP automodification reactions as described above in the presence of 100 μM NAD^+^. Reactions were stopped by the addition of 10 μM talazoparib. Reactions were divided and further incubated in the presence or absence of 0.5 μM hydrolases for 60 min at 30 °C as indicated. Reactions were stopped by the addition of LDS sample buffer (Life Technologies) and incubation at 95 °C for 3 min. Samples were analysed by CBB staining and immunoblot.

### NAD^+^ quantification assays

Quantification of NAD^+^ was performed using NAD^+^/NADH Glo™ assay (Promega) according to the manufacturer’s instructions for in vitro ADP-ribosylation assays. Briefly, proteins (250 nM) were incubated with DNA damage mimetic (300 nM) and NAD^+^ (10 μM) at RT and reactions stopped at indicated timepoints by addition of talazoparib (500 nM; Supplementary Fig. 9). Samples were mixed 1:1 with NAD^+^/NADH-Glo™ detection reagent, incubated for 20 min at RT and luminescence read using a Spark® microplate reader (Tecan) in 384-white, shallow well white ProxiPlates (Revvity). Data were background corrected (no NAD^+^ addition) and normalised (no PARP reaction) before analysis in Prism v. 10.6.1 (GraphPad).

### PAR binding assays

Synthesis of free PAR and determination of its noncovalent interactions with different *Af*-PARP1 constructs was based on a previously described method by Malanga and Althaus^91^. Briefly, auto-PARylation of 5 μM of *Af*-PARP1 DEDAAA was achieved in presence of activated DNA (BPS Bioscience) as described above with the following modification: incubate was stepwise first with 100 μM 6-biotin-17-NAD^+^ (Tocris Bioscience) for 30 min at 30 °C followed by addition of 4.9 mM NAD^+^ and further incubation for 1 hr. Subsequently, 40 U of benzonase were added, and the reaction was incubated for 30 min at 37 °C. Reaction was stopped by the addition of an equal volume of ice-cold 40% (w/v) TCA and incubation for 15 min on ice. Precipitant was sedimented by centrifugation and washed twice with 20% (w/v) TCA, thrice with 96% ethanol, and once with acetone. The pellet was air dried and resuspended in 10 mM Tris-NaOH (pH 12) and 1 mM EDTA and incubated for 2 h at 37 °C. Reaction was diluted 1:1 with 50 mM TrisHCl (pH 7.4), 1 mM EDTA and free PAR chains purified by phenol/chloroform/isoamyl alcohol (25:24:1) extraction with chloroform wash.

For determination of PAR binding, 20 pmole of protein were immobilised by slot-blot using a Hybri-Slot™ manifold (Bethesda Research Laboratories) on a nitrocellulose membrane. The membrane was washed thrice with TBS (10 mM Tris-HCl, pH 7.4, 150 mM NaCl) for 5 min and incubated overnight with diluted PAR in TBS-T (TBS containing 0.05% (v/v) Tween-20). Membrane was washed twice with TBS-T and blocked with 4% (w/v) milk powder in TBS-T. Bound biotin-PAR was detected using HRP-conjugated streptavidin (Millipore Cat. # 18-152) and visualised with SuperSignal™ West Pico PLUS chemiluminescent substrate.

### Circular dichroism (CD) spectroscopy

All measurements were conducted with freshly annealed DNAs (see above), and all measurement components were diluted to final concentrations with 20 mM HEPES, pH 8, 100 mM NaCl, 0.5 mM TCEP, and 2 % (v/v) glycerol. Qualitative CD spectra for the DNA-ligand binding interactions were performed at a DNA concentration of 29 μM in a 0.0058 cm pathlength CaF_2_ demountable cell. The DNA:ligand ratio used in these experiments was 1:2 at 29 μM DNA concentration and 58 μM ligand (WT or ΔBRCT).

CD experiments were performed using Module A and a nitrogen-flushed Chirascan-Plus spectrophotometer (Applied Photophysics Ltd, Leatherhead, UK) at the B23 Synchrotron Radiation CD (SRCD) beamline at Diamond Light Source Ltd. Data transfer and results obtained were processed using CDApps^92^ and analysed in Prism v. 10.6.1 (GraphPad).

### Size exclusion chromatography-coupled small-angle X-ray scattering (SEC-SAXS)

Experimental and analysis parameters are summarised in Tab. 1. Briefly, PARP proteins were purified as described above and analysed either alone or as a 1:1.2 molar complex with the indicated DNA. SAXS measurements were performed using the SEC configuration: capture of the elution peak in a 1.5 mm quartz capillary flow cell (1.6 mm path length) and data collected on an Eiger 4 M detector (Dectris). The X-ray wavelength and sample-to-detector distance were 0.9464 A and 3.67 m, respectively, corresponding to an accessible q-range of 0.0045 to 0.34 Å^−1^. SEC was achieved with an Agilent 1200 series HPLC and a Superdex S200 Increase 3.2/300 column (Cytiva) equilibrated with three column volumes of SAXS buffer (20 mM HEPES pH 7.5, 150 mM NaCl, 2 mM TCEP, 2.5 % (v/v) glycerol). During the elution, SAXS measurements were made using 3-s exposure frames. Data were acquired and reduced using the general data acquisition software (GDA; DLS) and DAWN Science (DLS)^93^, respectively. Data were analysed using ScÅtter IV (Bioisis) and deposited in the Small Angle Scattering Biological Data Bank^94^.

### Differential scanning fluorimetry (DSF)

Assays were performed essentially as described earlier^95^. Briefly, 4 μM of *Af*-PARP1 WT or mutants in presence or absence of 4 μM DNA-1 and/or 1 mM BAD (Sigma-Aldrich, cat no. SML3494) as indicated and 5× SYPRO® Orange (ThermoFisher Scientific) were incubated in assay buffer (50 mM Tris HCl [pH 8], 200 mM NaCl, 1 mM MgCl_2_, 1 mM DTT) for 10 min at RT followed by thermal denaturation in MicroAmp® Fast plates (Life Technologies) in a QuantStudio 7 Pro (ThermoFisher Scientific). The initial temperature of 24 °C was held for 2.5 min, followed by a ramp up to 97.5 °C using a step of 0.5 °C/45 s and a final 120 s pause at 98 °C. Curves were fit using Prism v. 10.6.1 (GraphPad). For analysis recorded data were cropped two data points after the maximum fluorescence signal was reached and the resulting curves were fitted using the Boltzmann equation

1 $$F={BOTTOM}+\frac{({TOP}-{BOTTOM})}{1+{e}^{\left(\frac{({T}_{m}-T)}{{SLOPE}}\right)}}$$

with *T*_m_, melting temperature; *T*, temperature; and SLOPE, steepness of curve.

### Live-cell microscopy

#### Widefield fluorescence microscopy

Representative images of transfected 2H11 cells were captured on a widefield Olympus APX100 microscope equipped with an Olympus 40×/0.95 UPlanXAPO objective lens, a U-LGPS LED light source set to 6% intensity and a Hamamatsu Orca Fusion C14440 sCMOS camera operating in 16-bit mode. The microscope was controlled with CellSense APX software (v.4.4).

Hoechst fluorescent nuclei were captured using a DAPI filter set (*λ*_ex_ 350/50 nm; *λ*_em_ 460/50 nm), and mScarlet-I3 was captured using an mCherry filter set (*λ*_ex_ 560/40 nm; *λ*_em_ 630/75 nm). Gradient contrast images were also captured. Images were captured using the autoexposure function of the camera, which did not surpass 100 ms exposure time.

#### Confocal fluorescence microscopy

Images were captured on a Nikon Ti2 Andor Dragonfly505 laser scanning confocal microscope, equipped with 405 nm (500 mW) and 561 nm (500 mW) lasers matched with 445/46 nm and 594/43 nm bandpass emission filters, respectively, a Nikon 100×/1.4 PlanApo oil immersion objective lens, 40 μm pinhole spinning disk, and Andor Sona sCMOS camera operating at 12-bit mode. Samples were illuminated at 38% (405 nm) and 20% (561 nm) intensity for 60 and 175 ms exposure, respectively. For Z-stack imaging, slices were acquired from above to below the cell plane with a 150 nm Z-step size. Image acquisition was controlled by Andor Fusion software (v.2.6).

For co-localisation analysis, out-of-focus z-slices were removed by identifying slices with null Manders’ coefficient values, as described by Aditya and colleagues^96^. Nuclei in the resulting Z-stacks were segmented on a slice-by-slice basis using Cellpose (v. 4.0.8) in a custom Python (v. 3.9.23) script (Source Data), and the computational environment used for analysis is provided as a conda environment file (Source Data).

Co-localisation analysis was performed in Fiji (ImageJ v. 1.54p) using the BIOP JACoP plugin (accessed February 2026)^97^. Manders’ overlap coefficients were calculated for each segmented nucleus using the Hoechst and mScarlet-I3 fluorescence channels, with analysis restricted to nuclear regions. Automatic thresholding was applied using the Costes method to minimise background bias. The Manders’ M_2_ coefficients, which indicate how much and how well the fluorescent signals of mScarlet-I3 overlapped with the Hoechst signals, were analysis in Prism v. 10.6.1 (GraphPad).

### Inference of phylogenetic relationships and sequence similarities

Alignments of full-length fungal BRCT PARPs (Supplementary Table 1), PARP proteins from *Animalia*, *Plantae,* and *Fungi* species (Supplementary Tables 2 and 3), and BRCT domain alignment across *Animalia*, *Plantae,* and *Fungi* species (Supplementary Table 5) were generated using JalView v. 2.11^98^ using the Mafft L-INS-i algorithm^99^. For interkingdom comparison, the PARP WGR-CAT regions were extracted from their sequential context based on crystallographic data and the resulting truncated sequences were re-aligned with Mafft L-INS-i. The evolutionary histories of the PARP WGR-CAT region and fungal PARPs were inferred using the maximum-likelihood method and Le_Gascuel_2008^100^ model with an automatically obtained initial tree for the heuristic search by applying the maximum-parsimony method. The analysis was carried out using a site coverage of 95% with the partial-deletion option. Confidence levels were estimated using 500 cycles of the bootstrap method. Evolutionary analyses were conducted in MEGA11^101^. All alignments shown were created using JalView v2.11 and the Mafft L-INS-i algorithm implemented therein, and representations were created with ALINE^102^.

### Domain identity analysis

Indicated protein sequences were aligned in JalView v. 2.11^98^ using the Mafft L-INS-i algorithm^99^ implemented therein. Alignments were trimmed to domain boundaries using structural and sequential information. Pairwise percentage identities were calculated in R using the Biostrings package^103^. For each sequence pair, identity was defined as the number of identical amino acids divided by the total number of overlapping ungapped positions, expressed as a percentage. The results were visualised using heatmapper^104^.

### Reporting summary

Further information on research design is available in the Nature Portfolio Reporting Summary linked to this article.

## Supplementary information

Supplementary Information

Reporting Summary

Transparent Peer Review File

## Source data

Source Data

## Data Availability

The partial mRNA sequence of *Lo*PARP2 and the coding DNA sequence of *Lo*PARP3 were deposited in GenBank under the accession numbers PX314872 and PX437880, respectively. SAXS profiles and pair distribution functions were uploaded to SASBDB and are available under the accession codes SASDXE9 (*Af*-PARP1 apo), SASDXF9 (*Af*-PARP1 ΔBRCT apo), SASDXG9 (*Af*-PARP1:DNA-1), and SASDXH9 (*Af*-PARP1 ΔBRCT:DNA-1). AlphaFold 3 models were deposited to ModelArchive and are available under the model IDs ma-wejsd (*Af*-PARP1:DNA-1:NAD^+^) and ma-8ypox (*Af*-PARP1:NAD^+^). All other data generated in this study are provided in the main manuscript and its Supplementary Information files. Source data are provided with this paper.
