# Supplementary material for: The BRCT domain enhances DNA binding and catalytic efficiency of fungal PARPs
Source: Nat Commun. 2026 Jul 24;17:9024. doi: 10.1038/s41467-026-75844-6 (PMC13507078; doi:10.1038/s41467-026-75844-6)
Supplement: Supplementary file 1 [file 41467_2026_75844_MOESM1_ESM.pdf]

Supplementary Information

The BRCT domain enhances DNA binding and catalytic efficiency of fungal PARPs

Adam G. Bainbridge[1], Tiberiu-Marius Gianga[2], Rohanah Hussain[2], Callum Parkin[1], Adam D. Longhurst[1], Nicolas Helmstetter[1], Rhys A. Farrer[1], Sandra Catania[1], Giuliano Siligardi[2], Johannes Gregor Matthias Rack[1,*]

1 Medical Research Council Centre for Medical Mycology at the University of Exeter, Department of Biosciences, Faculty of Health and Life Sciences, Geoffrey Pope Building, Stocker Road, Exeter, Devon, EX4 4QD, UK

2 Beamline 23, Diamond Light Source Ltd, Harwell Science and Innovation Campus, Oxfordshire, OX11 0DE, UK

***Correspondence:** j.rack@exeter.ac.uk

Content

- Supplementary Figures
- Supplementary Tables
- Supplementary Notes
- Supplementary References

Supplemental Figures

Supplementary Figure 1. Schematic representation of *A. fumigatus* PARPs.

Abbreviations: 4C*, PARP6/8-type C4 zinc finger; βB, beta-barrel; HE, helical extension; RWD, after the three major RWD-containing proteins: RING finger-containing proteins, WD-repeat-containing proteins, and yeast DEAD (DEXD)-like helicases; UBCc, ubiquitin-conjugating enzyme E2 catalytic domain

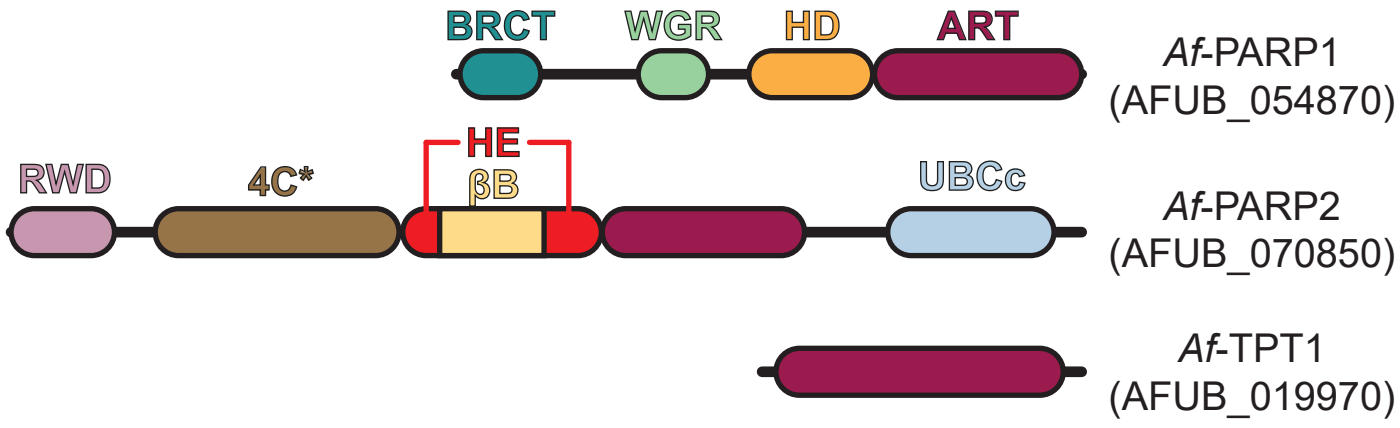

Supplementary Figure 1.

Supplementary Figure 2. Multiple sequence alignment of PARP-derived HD-ART domains from fungi, plants, and animals.

Multiple sequence alignment of selected PARP-derived HD-ART domains from *Fungi*, *Animalia*, and *Plantae*. Residue numbering corresponds to *Af*-PARP1 and key residues for allosteric activation in the HD (gangia, Ϫ) as well as MAR/PAR-selectivity in the ART domain (ksi, Ⲝ) are indicated above the alignment. Domain boundaries are indicated as coloured lines above the alignment yellow (HD) and red (ART) with golden segment indicating the αF helix in the HD and lightred the D-loop in the ART domain, respectively. Secondary structures for *Af*-PARP1 (AlphaFold 3 model; green), *h*PARP2 (PDB 6F5B[1]; red), and *h*PARP4 (PDB 8SX1[2], purple) are given below the alignment.

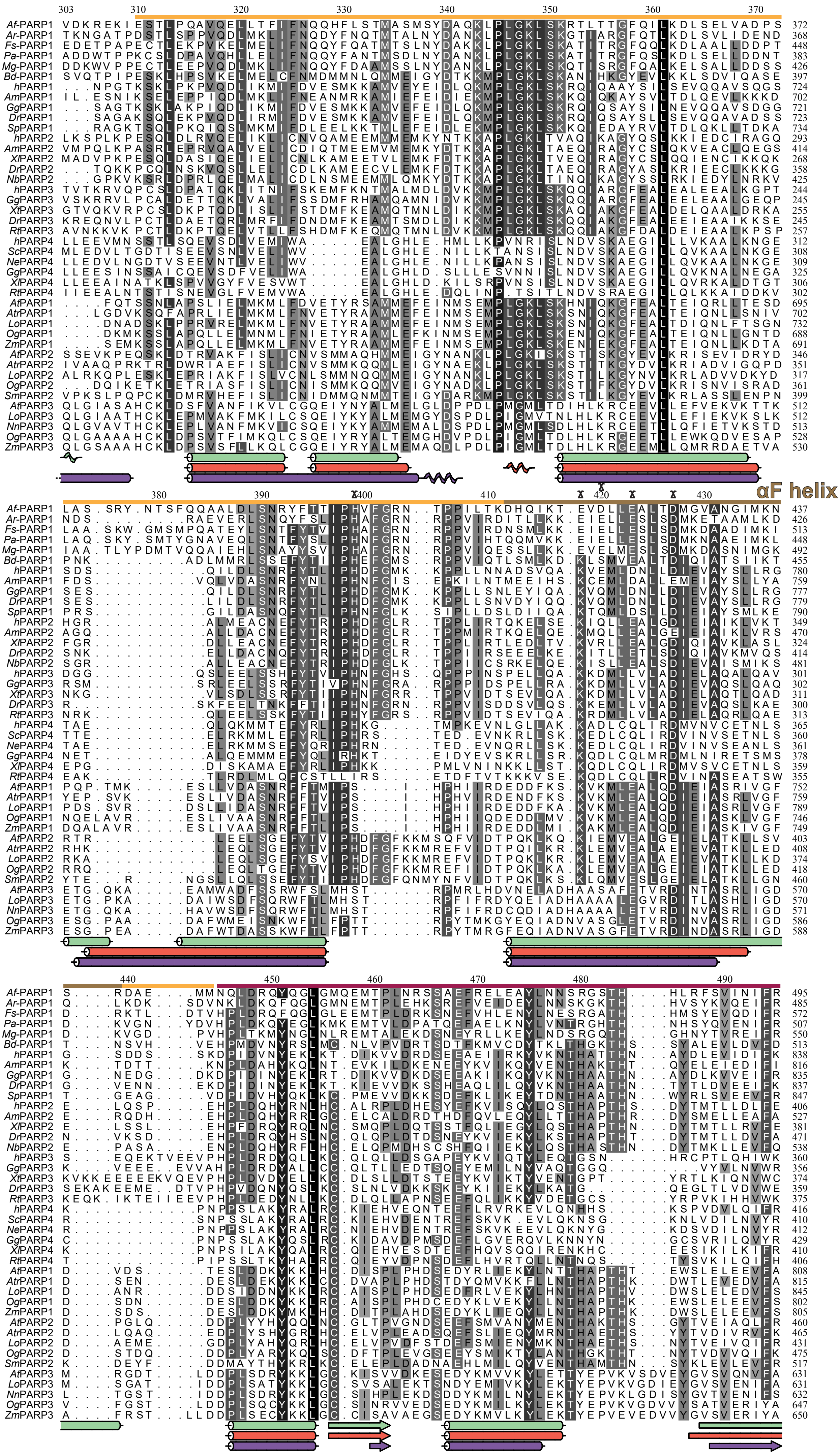

Supplementary Figure 2. (1/2)

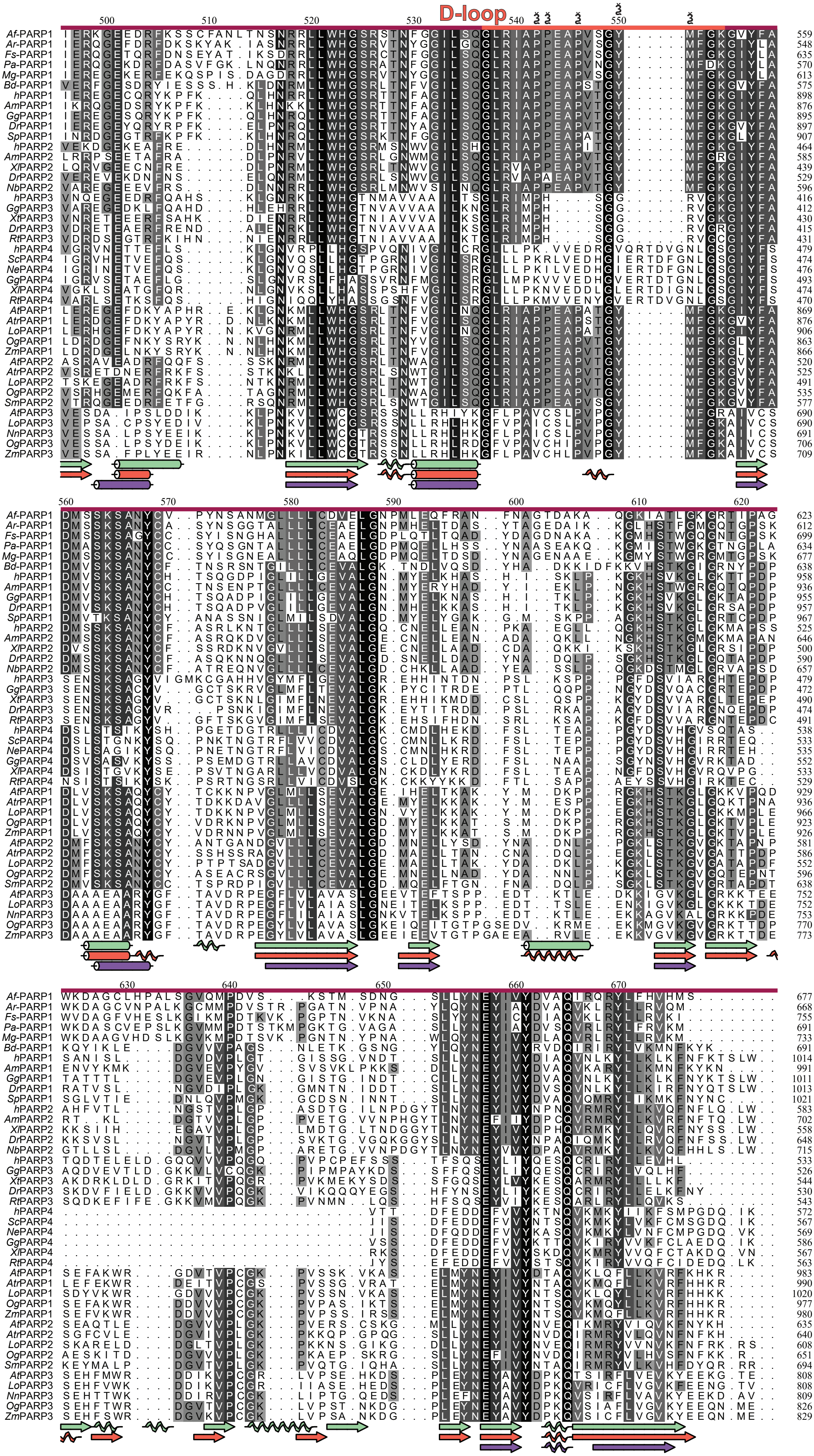

Supplementary Figure 2. (2/2)

Supplementary Figure 3. Characterisation of the *Af*-PARP1-derived ADP-ribosylation signal.

(a) Assessment of the *Af*-PARP1-derived ADP-ribosylation signal by immunoblot using an antibody and bind reagent panel with specificity against MARylation, PARylation or both (pan). 0.5 µM of *Af*-PARP1 WT, ΔBRCT, and E657Q as well as *h*PARP2 (PAR cntr) and *h*PARP3 (MAR cntr) were automodified in presence of activated DNA (BPS Bioscience). Shown is a representative result of three independent experiments. Uncropped images are available in the Source Data file.

(b) Assessment of the ability of various (ADP-ribosyl)hydrolases with know linkage specificities to reverse *Af*-PARP1 E657Q auto-MARylation. Shown is a representative result of two independent experiments. Uncropped images are available in the Source Data file.

(c) Assessment of the ability of various (ADP-ribosyl)hydrolases with know linkage specificities to reverse *Af*-PARP1 WT auto-PARylation. As ADP-ribose polymerse can interfere with cleave of the terminal linkage, de-ADP-ribosylation assays were carried out in presence of PARG to degrade polymers. Shown is a representative result of two independent experiments. Uncropped images are available in the Source Data file.

(d) Chemical stability of *Af*-PARP1-derived ADP-ribosylation was assessed post-auto(ADP-ribosylation). Stability under acidic and alkaline condition was assessed with HCl and NaOH, respectively. Stability under high ionic strength conditions and in presences of hydroxylamine was assessed at pH 7 stabilised with MOPS•NH_4OH. Shown is a representative result of three independent experiments. Uncropped images are available in the Source Data file.

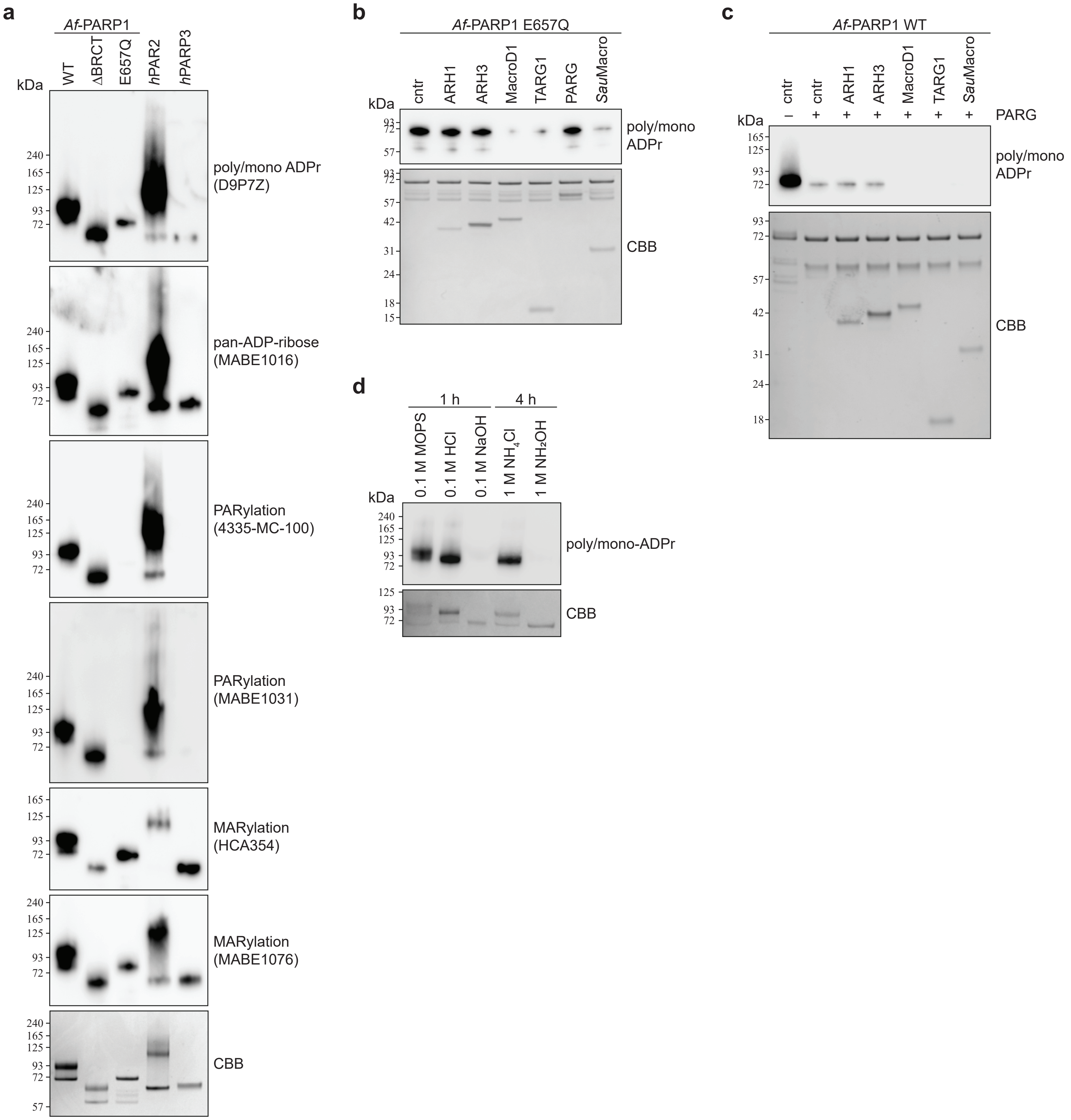

Supplementary Figure 3.

Supplementary Figure 4. Activation requirements of *Af*-PARP1 in comparison to animal and plant PARPs.

(a) EMSA assay assessing the overhang length requirement of interaction with *Af*-PARP1. *Af*-PARP1 (5 µM) were incubated with DNA duplexes with indicated 3' overhang length (1 µM) for 1 h, separated via native PAGE and the DNA visualised with SYBR safe staining. DNA-16/17 duplex was used as blunt-end control. Shown is a representative result of three independent experiments. Uncropped images are available in the Source Data file.
(b) EMSA assay assessing the ability of *Af*-PARP1 to interact with ssDNA either 5'-phosphoylated (DNA-16) or unmodified (DNA-17.5). Assay was conducted as in (a) and ssDNA visualised using Diamond™ nucleic acid dye (Promega). Shown is a representative result of two independent experiments. Uncropped images are available in the Source Data file.
(c) EMSA assay assessing the DNA damage binding specificity of *h*PARP2, 3, and *Lo*PARP2. PARPs (5 µM) were incubated with DNA mimetics (1 µM) for 1 h, separated via native PAGE and the DNA visualised with SYBR safe staining. Shown is a representative result of three (*Lo*PARP2) or two (*h*PARP2 and *h*PARP3) independent experiments. Uncropped images are available in the Source Data file.
(d) Assessment of *Af*-PARP1 activation by different 3' overhang length. *Af*-PARP1 (0.5 µM) was incubated with DNA (0.6 µM) and NAD^+ (500 µM) and formation of PAR detected using immunoblot. Shown is a representative result of two independent experiments. Uncropped images are available in the Source Data file.
(e) Assessment of *Af*-PARP1 activation by ssDNA. *Af*-PARP1 (0.5 µM) was incubated with DNA (0.6 µM) and NAD^+ (500 µM) and formation of PAR detected using immunoblot. Shown is a representative result of two independent experiments. Uncropped images are available in the Source Data file.
(f) Assessment of *h*PARP2, 3, and *Lo*PARP2 catalytic activities in presence of different DNA damage mimetics. PARPs (0.5 µM) were incubated with DNA (0.6 µM) and NAD^+ (500 µM) and formation of PAR detected using immunoblot. Shown is a representative result of two independent experiments. Uncropped images are available in the Source Data file.

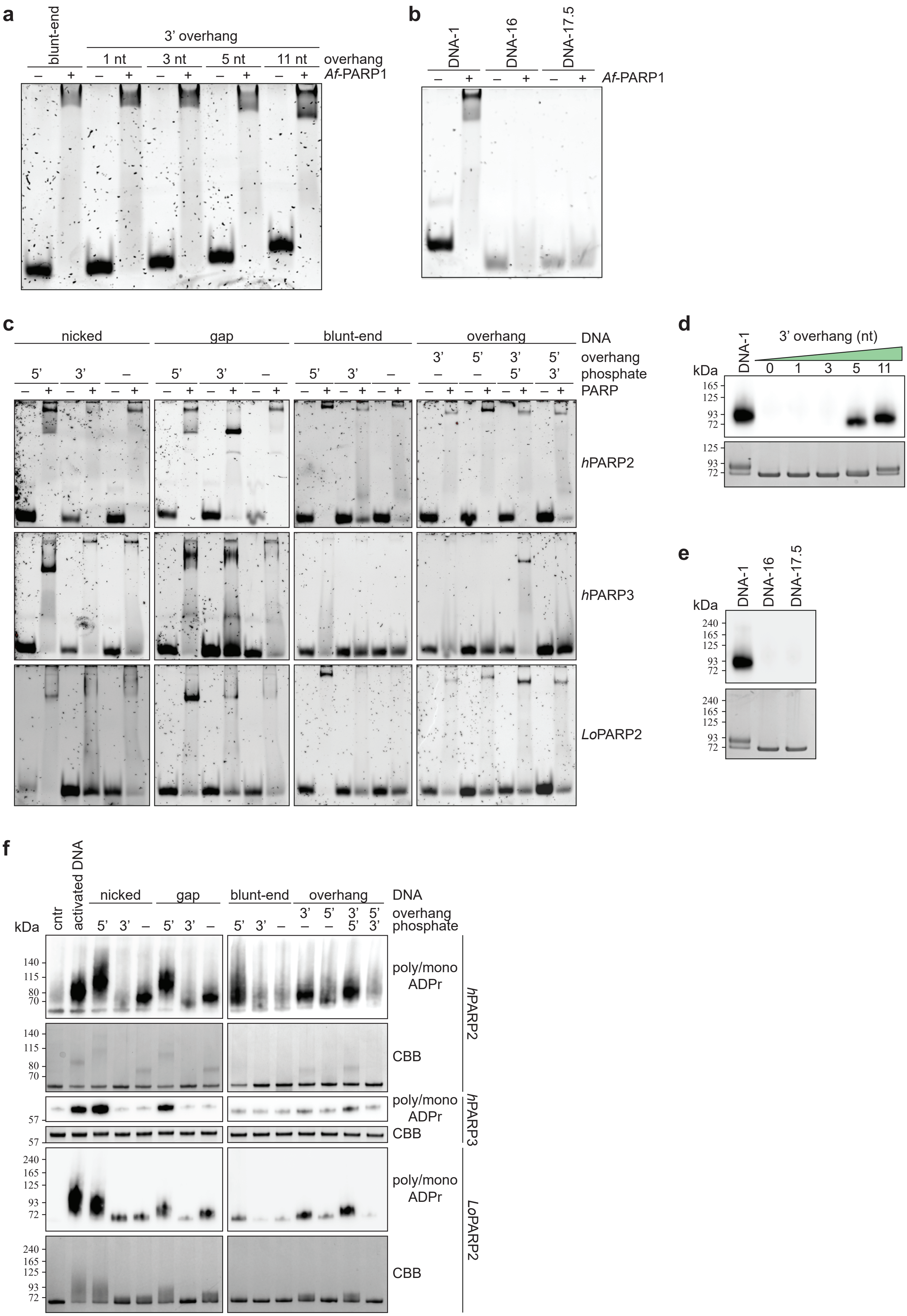

Supplementary Figure 4.

Supplementary Figure 5. DNA damage recognition by the *Af*-PARP1 WGR domain.

(a) Multiple sequence alignment of selected PARP-derived WGR domains from *Fungi*, *Animalia*, and *Plantae*. Key DNA interaction residues are indicated by koppa (ϟ) above the alignment and residue numbering corresponds to *Af*-PARP1. Secondary structures for *Af*-PARP1 (AlphaFold 3 model; green) and *h*PARP2 (PDB 6F5B[1]; red) are given below the alignment.
(b) Per-residue confidence estimate (pLDDT) mapped onto the predicted structure for *Af*-PARP1:NAD^+ are shown in the top panel. The expected position error (EPE) of the predicted complex is shown in the bottom panels.
(c) Ribbon representation of the structural alignment of *Af*-PARP1 WGR domain (AlphaFold 3 model) with the experimentally determined WGR domains from *h*PARP2 (PDB 6F5B[1]) and 3 (PDB 2EOC).
(d) EMSA assay assessing the influence of Ser241 on the ability of the WGR domain to bind double-strand breaks. *Af*-PARP1 WT or S241Q (5 µM) were incubated with DNA mimetic (1 µM) for 1 h, seprated via native PAGE and DNA visulised with SYBR safe staining. Shown is a representative result of two independent experiments. Uncropped images are available in the Source Data file.
(e) Assessment of the influence of Ser241 on the catalytic activity of *Af*-PARP1 in presence of 5'-phosphorylated DNA double-strand break mimetic. WT or S241Q *Af*-PARP1 (0.5 µM) were incubated with DNA (0.6 µM) and NAD^+ (500 µM) and formation of PAR detected using immunoblot. Uncropped images are available in the Source Data file.
(f) Ribbon-liquorice representation of the DNA coordination by *h*PARP2 (PDB 6F5B[1]; red/white) overlaid with the *Af*-PARP1 WGR domain derived from the apo AlphaFold 3 model (green). Residues indicated represent *Af*-PARP1 numbering.
(g) Ribbon-liquorice representation of the structural alignment of *Af*-PARP1:DNA AlphaFold 3 model (only WGR domain shown) with the experimentally determined WGR:DNA complex from *h*PARP2 (PDB 6F5B[1]). *Af*-PARP1 Tyr281 is indicated for orientation.

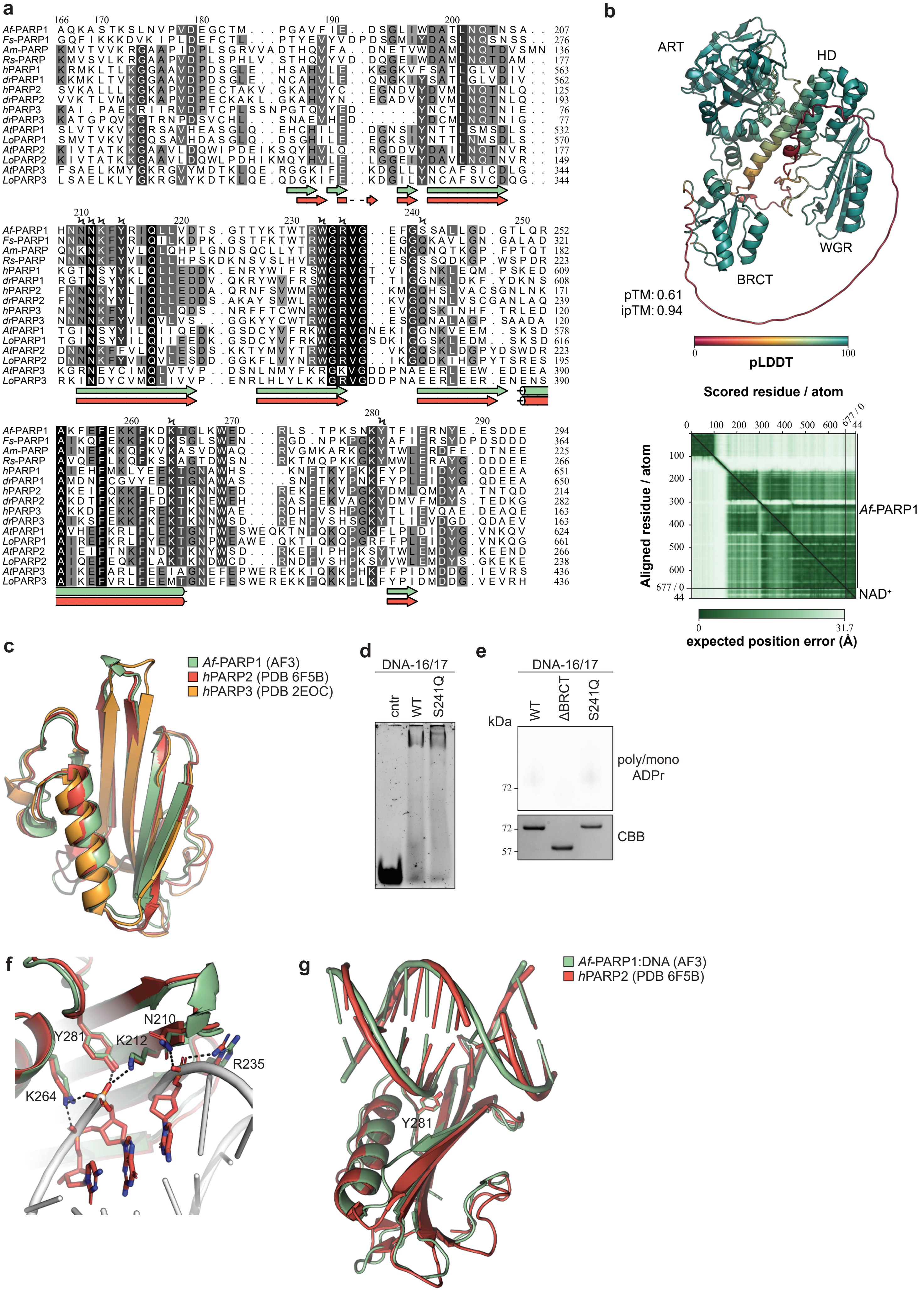

Supplementary Figure 5.

Supplementary Figure 6. The WGR-HD and HD-ART interfaces play a crucial role for *Af*-PARP1 activation.

(a) Ribbon-liquorice representation of the structural alignment of *Af*-PARP1 (lightred) with *h*PARP1 (white; PDB 4OPX[3]), and *h*PARP2 (coloured by domains: green, WGR; yellow, HD; red, ART; PDB 6X0L[4]). Approximate positions of the close ups are given as black boxes on the overview panel and indicated residues represent *Af*-PARP1.
(b+c) DSF experiment using full-length *Af*-PARP1 WT or mutants in the presence (b) or absence (c) of DNA-1 as well as with or without addition of BAD. Data represent three biological replicas and T_m values are given as mean values ± SD.

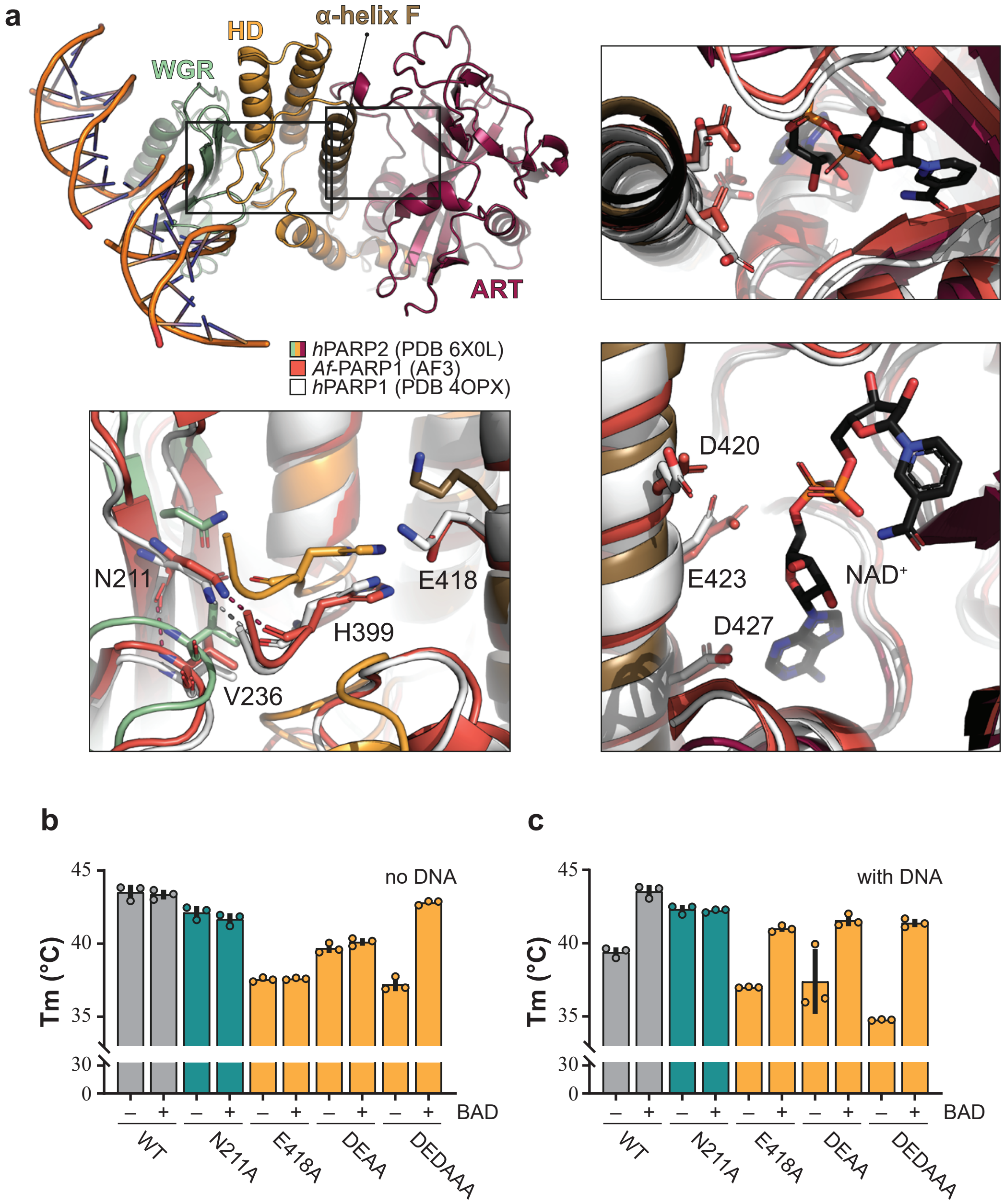

Supplementary Figure 6.

Supplementary Figure 7. The BRCT domain of *Af*-PARP1 is a DNA but not PAR binding domain.

(a) Per-residue confidence estimate (pLDDT) mapped onto the predicted ternary complex of *Af*-PARP1:DNA:NAD^+ are shown in the left panel. The expected position error (EPE) of the predicted complex is shown in the right panels.
(b) Model-to-model comparison for the BRCT:DNA-1 interaction. BRCT domains and DNA were extracted from their respective full-length *Af*-PARP1 models before alignment (r.m.d.s. scores ≤ 0.092 Å).
(c) Multiple sequence alignment of PARP-derived BRCT domains from *Fungi*, *Animalia*, and *Plantae*. Residues contributing to the predicted DNA interaction surface are indicated above the alignment by dei (†). Secondary structures of *Af*-PARP (green; AlphaFold 3-derived), *h*PARP1 (red; PDB 2COK), and *h*PARP4 (purple; PDB 9DEV[5]) are given below the alignment. Indexing indicates *Af*-PARP1 residue position.
(d) Pairwise identity matrix of isolated BRCT domains was calculated from multiple sequence alignments using Biostrings[6] and visualised using heatmapper[7].
(e) Ribbon overlay representation of the BRCT domains from *h*PARP1 (red; PDB 2COK), *h*PARP4 (purple; PDB 9DEV[5]) and *Af*-PARP1 (green; AlphaFold 3 model).
(f) The electrostatic potential surface of the isolated BRCT domain was prepared with PDB2PQR[8,9] and calculated using the APBS electrostatics plugins[10] in PyMOL (Schrödinger).
(g) PAR binding slot-blot assay. 20 pmole of indicated protein were immobilised on a nitrocellulose membrane and incubated using free chain-start biotinylated PAR (see Methods). BSA was used as a negative and C-terminally mScarlet-I3-tagged WWE domain from RNF146 (WWE-Red) as positive control. Binding of PAR to the immobilised protein was assessed by immunoblot utilising streptavidin-HRP. Shown is a representative result of three independent experiments. Uncropped images are available in the Source Data file.
(h) Schematic representation of the different *Af*-PARP1 constructs used in the microscopy experiments. For the domain deletion constructs (ΔBRCT and ΔBRCT2) the dotted boxes represent the deleted regions, whereas relative positions of the point mutations are indicated by lines.
(i) Representative images of sub-cellular localisation of *Af*-PARP1 WT and mutants indicated in (g) in murine 2H11 cells using transient transfection of N-terminally mScarlet-I3-tagged constructs. Cells were visualised by gradient contrast (GC) and nuclei by Hoechst 33342 staining. The scale bar represents 20 μM.

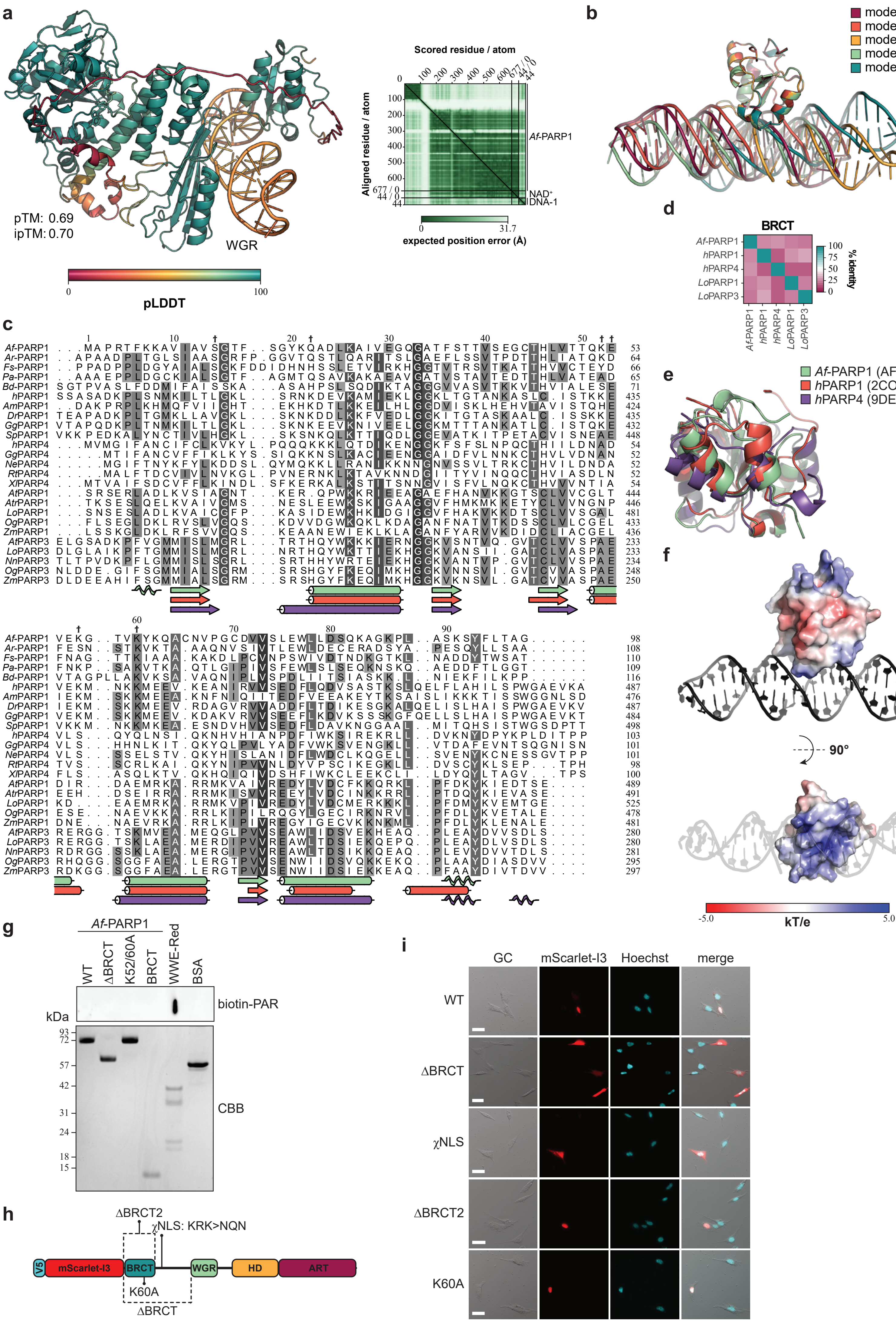

Supplementary Figure 7.

Supplementary Figure 8. SAXS quality parameters and CD near-UV spectra for *Af*-PARP1 and its DNA complexes.

(a) Log_{10} intensity plots for *Af*-PARP1 WT and ΔBRCT in their apo and DNA-1 bound forms.
(b) Guinier fits and normalised fit residuals for *Af*-PARP1 WT and ΔBRCT in their apo and DNA-1 bound forms.
(c) CD near-UV spectra of *Af*-PARP1 WT and ΔBRCT in presence and absence of DNA-1 or -2. Expected complex spectra (summation of protein and DNA apo spectra) are shown as dotted line. Indicative of B-DNA is a maximum near 280 nm, in contrast to A-DNA (near 265 nm) or Z-DNA (near 260 nm)[11].
(d) EMSA assay assessing the ability of *Af*-PARP1 WT or ΔBRCT to interact with nicked (DNA-1) or gap (DNA-2) 5'-phosphorylated DNA. Experiment was conducted by incubating constant concentration of the respective DNAs (0.5 μM) with indicated protein in a 2-fold dilution series starting at 16 μM protein. Shown is a representative result of two independent experiments. Uncropped images are available in the Source Data file.

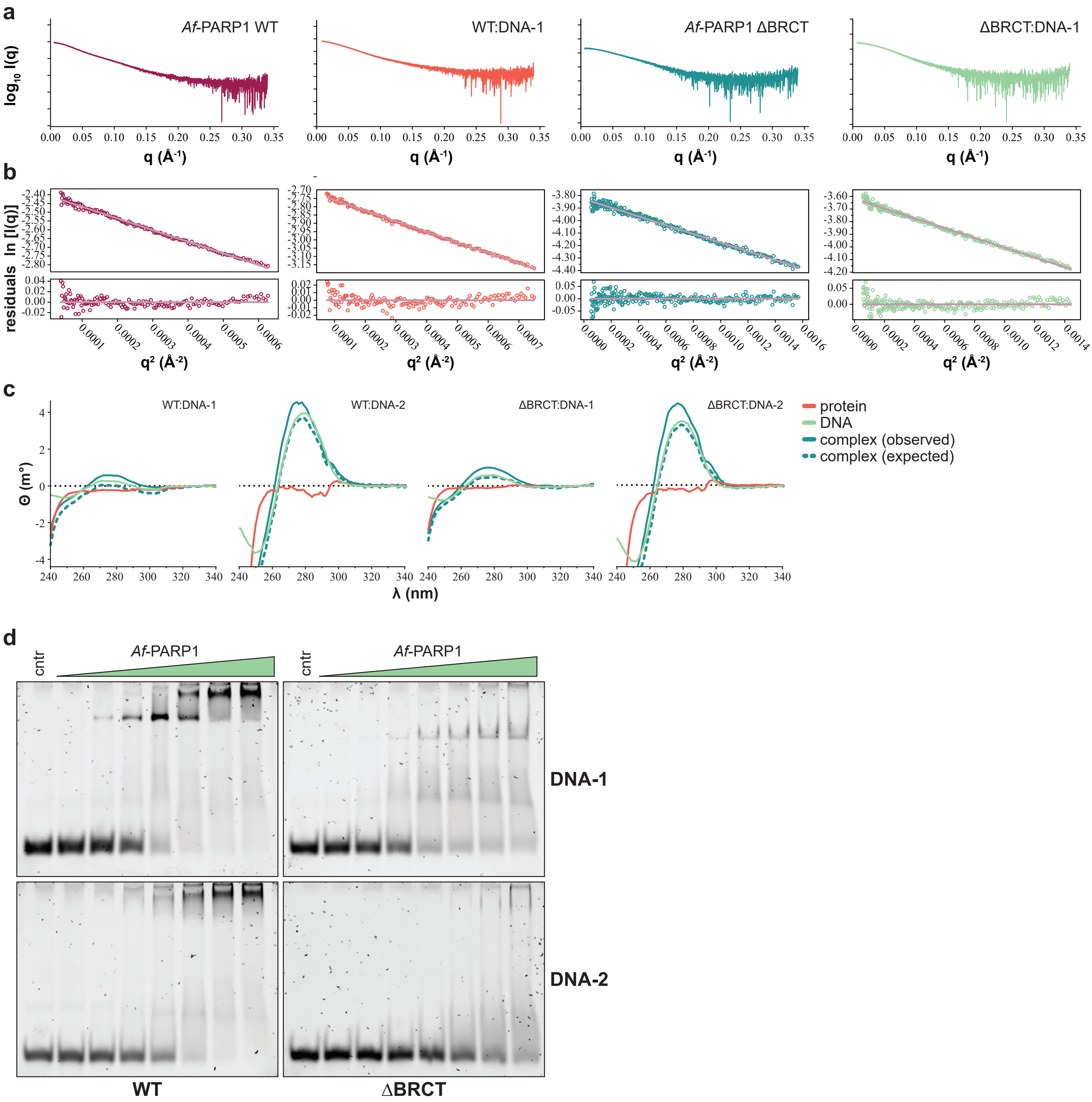

Supplementary Figure 8.

Supplementary Figure 9. Talazoparib is an efficient *Af*-PARP1 inhibitor.

The influence on talazoparib, a *h*PARP1 inhibitor, on the catalytic activity of *Af*-PARP1 was assessed. *Af*-PARP1 (0.5 µM) was incubated in presence of absence of talazoparib (1 µM) with activated DNA and NAD^+ (500 µM) and formation of PAR detected using immunoblot. Shown is a representative result of three independent experiments. Uncropped images are available in the Source Data file.

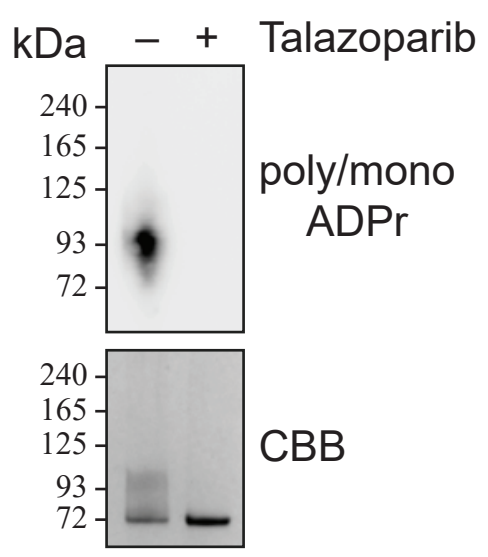

Supplementary Figure 9.

Supplemental Tables

Supplementary Table 1. Sequences used for comparison of fungal, BRCT domain-containing PARPs.

species	phylogenetic class	accession number[i]
Ascomycota		
Aaosphaeria arxii CBS 175.79	*Dothideomycetes*	XP 033387550
Alternaria rosae	*Dothideomycetes*	XP 046025914
Ascochyta lentis	*Dothideomycetes*	KAF9695564
Bipolaris sorokiniana ND90Pr	*Dothideomycetes*	XP 007705677
Corynespora cassiicola Philippines	*Dothideomycetes*	PSN74498
Decorospora gaudefroyi	*Dothideomycetes*	KAF1835861
Didymella sp. IMI 355093	*Dothideomycetes*	KAJ4376697
Didymosphaeria variabile	*Dothideomycetes*	XP 056068963
Diplodia corticola	*Dothideomycetes*	XP 020125224
Dothidotthia symphoricarpi CBS 119687	*Dothideomycetes*	XP 033519213
Lasiodiplodia hormozganensis	*Dothideomycetes*	KAK0663694
Lentithecium fluviatile CBS 122367	*Dothideomycetes*	KAF2675763
Lizonia empirigonia	*Dothideomycetes*	KAF1364362
Ophiobolus disseminans	*Dothideomycetes*	KAF2828069
Paraphoma chrysanthemicola	*Dothideomycetes*	KAH7081044
Periconia macrospinosa	*Dothideomycetes*	PVH96833
Phaeosphaeriaceae sp. PMI808	*Dothideomycetes*	KAH8731672
Phaeosphaeriaceae sp. SRC1lsM3a	*Dothideomycetes*	OAL05067
Plenodomus biglobosus	*Dothideomycetes*	KAH9869979
Stagonosporopsis vannaccii	*Dothideomycetes*	KAJ4987088
Aspergillus fumigatus	*Eurotiomycetes*	KAH1273305
Emergomyces africanus	*Eurotiomycetes*	OAX79141
Fonsecaea nubica	*Eurotiomycetes*	XP_022497109
Fonsecaea pedrosoi CBS 271.37	*Eurotiomycetes*	XP_013279927
Histoplasma capsulatum	*Eurotiomycetes*	QSS65792
Paracoccidioides brasiliensis	*Eurotiomycetes*	ODH29465
Penicillium antarcticum	*Eurotiomycetes*	XP 058320424
Phialophora macrospora	*Eurotiomycetes*	KIW70895
Talaromyces marneffei	*Eurotiomycetes*	XP_054119787
Geoglossum umbratile	*Geoglossomycetes*	KAI9780519
Xylographa bjoerkii	*Lecanoromycetes*	MCJ1389085
Xylographa parallela	*Lecanoromycetes*	MCJ1418709
Cadophora sp. MPI-SDFR-AT-0126	*Leotiomycetes*	KAH7393623
Hyaloscypha finlandica	*Leotiomycetes*	KAH8773067
Mollisiaceae sp. DMI_Dod_QoI	*Leotiomycetes*	KAE8441035
Pseudogymnoascus sp. VKM F-3775	*Leotiomycetes*	KFY12553
Pseudogymnoascus sp. VKM F-4513 (FW-928)	*Leotiomycetes*	KFY37269
Rhynchosporium agropyri	*Leotiomycetes*	CZS90872
Lichina confinis	*Lichinomycetes*	KAI9755981
Arthrobotrys entomopaga	*Orbiliomycetes*	KAF3919977
Drechslerella stenobrocha 248	*Orbiliomycetes*	EWC46572
Orbilia blumenaviensis	*Orbiliomycetes*	KAK6360592
Orbilia oligospora ATCC 24927	*Orbiliomycetes*	XP_011123834
Choiromyces venosus 120613-1	*Pezizomycetes*	RPA93621
Morchella sextelata	*Pezizomycetes*	XP_044696167
Tirmania nivea	*Pezizomycetes*	KAF8429243
Tricharina praecox	*Pezizomycetes*	XP_051340276
Colletotrichum chlorophyti	*Sordariomycetes*	OLN89070
Colletotrichum higginsianum IMI 349063	*Sordariomycetes*	XP_018159871
Fusarium graminearum	*Sordariomycetes*	KAI6751906
Fusarium solani	*Sordariomycetes*	KAH7264506
Lomentospora prolificans	*Sordariomycetes*	PKS11272
Madurella fahalii	*Sordariomycetes*	GAB1313793
Magnaporthiopsis poae ATCC 64411	*Sordariomycetes*	KLU91359
Neurospora crassa	*Sordariomycetes*	KAK3502080
Magnaporthe grisea[ii]	*Sordariomycetes*	QBZ63443
Podospora anserina	*Sordariomycetes*	CDP24926
Sordaria macrospora	*Sordariomycetes*	XP 065946313
Trichoderma guizhouense	*Sordariomycetes*	OPB37503

species	phylogenetic class	accession number[i]
Protomyces lactucae-debilis	*Taphrinomycetes*	XP_040722560
Taphrina deformans PYCC 5710	*Taphrinomycetes*	CCG83847
Chytridiomycota		
Batrachochytrium dendrobatidis		KAJ8326010
Batrachochytrium salamandrivorans		KAH6568607
Boothiomyces sp. JEL0866		KAJ3319355
Fimicolochytrium jonesii		XP_052926319
Geranomyces variabilis		KAJ3168278
Powellomyces hirtus		TPX54139
Rhizophlyctis rosea		KAJ3046051
Spizellomyces punctatus DAOM BR117		XP_016604037
Synchytrium endobioticum		TPX43361
Thoreauomyces humboldtii		KAJ3021815
Entomophthoromycota		
Basidiobolus ranarum		KAK9708347
Glomeromycota		
Acaulospora colombiana		CAG8530387
Diversispora epigaea		RHZ86579
Funneliformis mosseae		CAG8696709
Geosiphon pyriformis		KAG9290740
Paraglomus occultum		CAG8457874
Rhizophagus clarus		GES76673
Mucoromycota		
Actinomortierella ambigua		KAG0259843
Benniella sp.		KAK3812104
Dissophora ornata		KAF8929984
Entomortierella parvispora		GJJ72712
Linnemannia elongata		KAK3819163
Mortierella sp. AM989		KAF9110161
Neocallimastigomycota		
Anaeromyces robustus		ORX86892
Neocallimastix lanati (nom. inval.)		KAG4108431
Piromyces finnis		ORX55486

(i) GenBank accession number
(ii) GenBank entry gives species name as *Pyricularia oryzae*

Supplementary Table 2. PARP sequences across the *Animalia*, *Fungi*, and *Plantae*.

species		
binominal name	common name	accession number[i]
Fungi		
Ascomycota		
Alternaria rosae		XP_046025914
Aspergillus fumigatus		KAH1273305
Fusarium solani		KAH7264506
Magnaporthe grisea[ii]	rice blast fungus	QBZ63443
Podospora anserina	friendly mould	CDP24926
Basidiomycota[iii]		
Amanita muscaria Koide BX008	fly agaric	KIL70591
Pholiota conissans		KAF9485628
Psilocybe cf. *subviscida*		KAF5309111
Rhizoctonia solani		KAF8680135
Trametopsis cervina	deer-coloured trametes	KAI0344320
Chytridiomycota		
Batrachochytrium dendrobatidis	amphibian chytrid fungus	KAJ8326010
Thoreauomyces humboldtii		KAJ3021815
Glomeromycota		
Diversispora epigaea		RHZ86579
Geosiphon pyriformis		KAG9290740
Mucoromycota		
Actinomortierella ambigua		KAG0259843
Linnemannia elongata		KAK3819163
Neocallimastigomycota		
Neocallimastix lanati (nom. inval.)		KAG4108431
Animalia		
PARP1-like		
Apis mellifera	honeybee	XP_624477
Danio rerio	zebrafish	NP_001038407
Gallus gallus	chicken	NP_990594
Homo sapiens	human	NP_001609
Strongylocentrotus purpuratus	Pacific purple sea urchin	XP_030854208
PARP2-like		
Alligator mississippiensis	American alligator	XP_006266632
Danio rerio	zebrafish	NP_001191199
Homo sapiens	human	NP_005475
Narcine bancroftii	lesser electric ray	XP_069796901
Xenopus laevis	African clawed frog	NP_001086535
PARP3-like		
Danio rerio	zebrafish	NP_956795
Gallus gallus	chicken	XP_040537823
Homo sapiens	human	NP_001003931
Rhincodon typus	whale shark	XP_048462464
Xenopus tropicalis	western clawed frog	NP_001120635
Plantae		
PARP1-like		
Amborella trichopoda		XP_011624679
Arabidopsis thaliana	mouse-ear cress	NP_850165
Oryza glaberrima	African rice	XP_052161742
Lathyrus oleraceus[iv]	garden pea	XP_050910876
Zea mays	maize	NP_001288538
PARP2-like		
Amborella trichopoda		XP_006855550
Arabidopsis thaliana	mouse-ear cress	NP_001329308
Oryza glaberrima	African rice	XP_052161977
Lathyrus oleraceus[iv]	garden pea	PX314872
Sphagnum magellanicum	Magellanic bogmoss	KAH9533036
PARP3-like		
Arabidopsis thaliana	mouse-ear cress	NP_001318618
Nelumbo nucifera	sacred lotus	XP_010271692
Oryza glaberrima	African rice	XP_052145480
Lathyrus oleraceus[iv]	garden pea	PX437880

**species**		
binominal name	common name	accession number[i]
Zea mays	maize	XP_008645748

(i) GenBank accession number
(ii) GenBank entry gives species by its anamorph name as *Pyricularia oryzae*
(iii) Fungal PARPs without BRCT domain
(iv) formerly *Pisum sativum*

Supplementary Table 3. PARP sequences utilised to generate HD-ART alignment.

species		
binominal name	common name	accession number[i]
Fungi		
Aspergillus fumigatus		KAH1273305
Alternaria rosae		XP_046025914
Fusarium solani		KAH7264506
Magnaporthe grisea[ii]	rice blast fungus	QBZ63443
Podospora anserina	friendly mould	CDP24926
Batrachochytrium dendrobatidis	amphibian chytrid fungus	KAJ8326010
Animalia		
PARP1-like		
Apis mellifera	honeybee	XP_624477
Danio rerio	zebrafish	NP_001038407
Gallus gallus	chicken	NP_990594
Homo sapiens	human	NP_001609
Strongylocentrotus purpuratus	Pacific purple sea urchin	XP_030854208
PARP2-like		
Alligator mississippiensis	American alligator	XP_006266632
Danio rerio	zebrafish	NP_001191199
Homo sapiens	human	NP_005475
Narcine bancroftii	lesser electric ray	XP_069796901
Xenopus laevis	African clawed frog	NP_001086535
PARP3-like		
Danio rerio	zebrafish	NP_956795
Gallus gallus	chicken	XP_040537823
Homo sapiens	human	NP_001003931
Rhincodon typus	whale shark	XP_048462464
Xenopus tropicalis	western clawed frog	NP_001120635
PARP4-like		
Homo sapiens	human	NP_006428
Notamacropus eugenii	Tammar wallaby	XP_072467755
Gallus gallus	chicken	NP_001164635
Xenopus laevis	African clawed frog	XP_041440456
Rhincodon typus	Whale shark	XP_048454258
Plantae		
PARP1-like		
Amborella trichopoda		XP_011624679
Arabidopsis thaliana	mouse-ear cress	NP_850165
Oryza glaberrima	African rice	XP_052161742
Lathyrus oleraceus[iii]	garden pea	XP_050910876
Zea mays	maize	NP_001288538
PARP2-like		
Amborella trichopoda		XP_006855550
Arabidopsis thaliana	mouse-ear cress	NP_001329308
Oryza glaberrima	African rice	XP_052161977
Lathyrus oleraceus[iii]	garden pea	PX314872
Sphagnum magellanicum	Magellanic bogmoss	KAH9533036
PARP3-like		
Arabidopsis thaliana	mouse-ear cress	NP_001318618
Nelumbo nucifera	sacred lotus	XP_010271692
Oryza glaberrima	African rice	XP_052145480
Lathyrus oleraceus[iii]	garden pea	PX437880
Zea mays	maize	XP_008645748

(i) GenBank accession number
(ii) GenBank entry gives species by its anamorph name as *Pyricularia oryzae*
(iii) formerly *Pisum sativum*

Supplementary Table 4. DNA damage mimetics used in this study.

name	sequence	scheme[i]
DNA-1	[5'P]CGGTCGATCGTAAGATCGACCGGCGCTGGAGCTTGCTC<br>CAGCGC	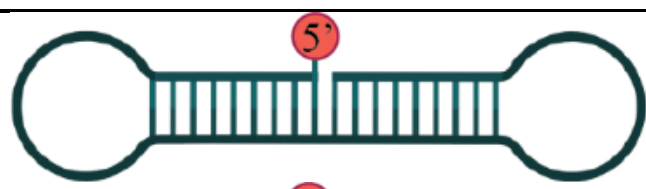
DNA-2	[5'P]CGGTCGATCGTAAGATCGACCGTGCGCTGGAGCTTGCT<br>CCAGCGC	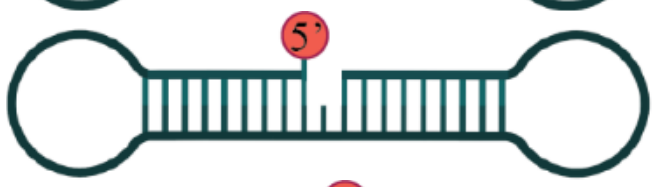
DNA-3	CGGTCGATCGTAAGATCGACCGTGCGCTGGAGCTTGCTCCA<br>GCGC[3'P]	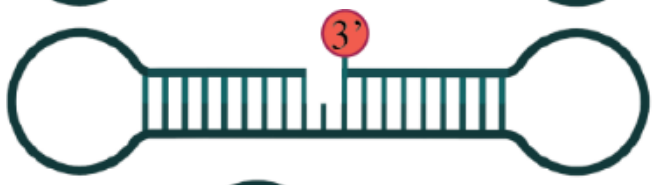
DNA-4	GGAAGTTCTTTTGAACTTCC	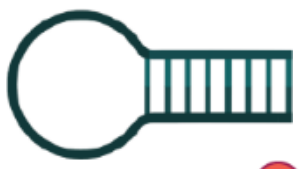
DNA-5	[5´P]GGAAGTTCTTTTGAACTTCC	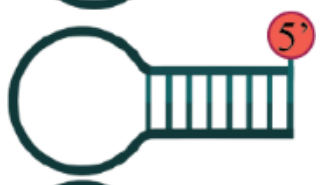
DNA-6	GGAAGTTCTTTTGAACTTCC[3´P]	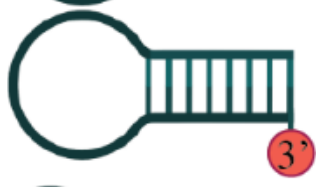
DNA-7	GGAAGTTCTTTTGAACTTCCTTGG	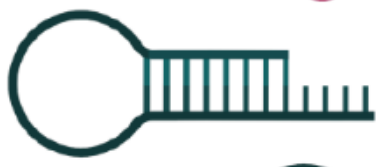
DNA-8	GCTAGGAAGTTCTTTTGAACTTCC	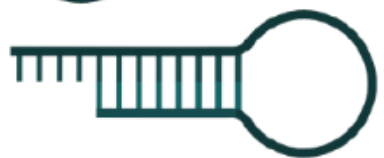
DNA-9	[5´P]GGAAGTTCTTTTGAACTTCCTTGG	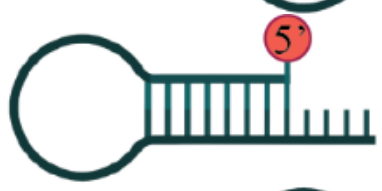
DNA-10	GCTAGGAAGTTCTTTTGAACTTCC[3´P]	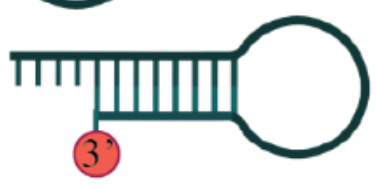
DNA-11	CGGTCGATCGTAAGATCGACCGGCGCTGGAGCTTGCTCCAG<br>CGC	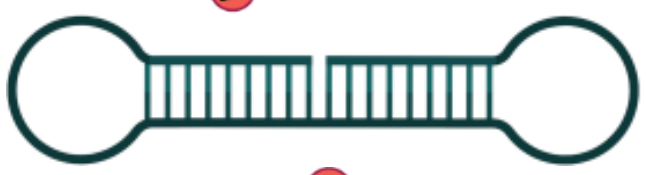
DNA-12	CGGTCGATCGTAAGATCGACCGGCGCTGGAGCTTGCTCCAG<br>CGC[3'P]	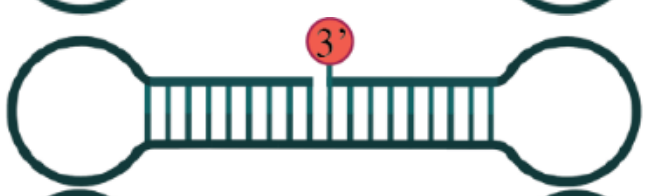
DNA-13	CGGTCGATCGTAAGATCGACCGTGCGCTGGAGCTTGCTCCA<br>GCGC	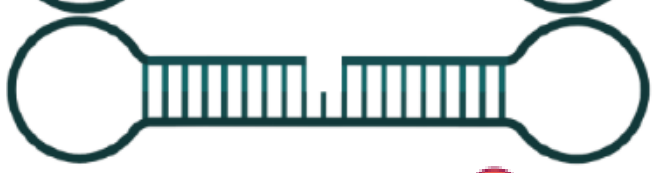
DNA16/17	DNA-16: [5'P]GACGACCCGGACACA<br>DNA-17: TGTGCTCCGGGTCGTC	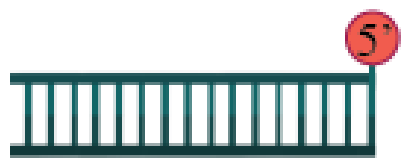
	DNA17.1: GACGACACGGAGCAGCC<br>DNA17.3: GACGACACGGAGCAGCCAA<br>DNA17.5: GACGACACGGAGCAGCCAACA<br>DNA17.11: GACGACACGGAGCAGCCAACATTATGG	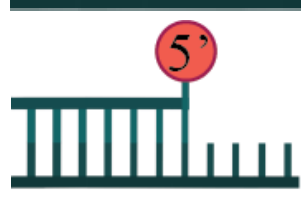 (ii)

(i) Schematic representations were created in BioRender. Rack, J. (2026) https://BioRender.com/n43w05j and modified in Adobe Illustrator 2026

(ii) DNA duplexes with varying overhang length are formed in combination with DNA-16. Overhang length in nt is indicate by number following the decimal point

Supplementary Table 5. PARP sequences utilised to generate BRCT alignment.

species		
binominal name	common name	accession number[i]
Fungi		
Aspergillus fumigatus		KAH1273305
Alternaria rosae		XP_046025914
Fusarium solani		KAH7264506
Podospora anserina	friendly mould	CDP24926
Batrachochytrium dendrobatidis	amphibian chytrid fungus	KAJ8326010
Animalia		
PARP1-like		
Apis mellifera	honeybee	XP_624477
Danio rerio	zebrafish	NP_001038407
Gallus gallus	chicken	NP_990594
Homo sapiens	human	NP_001609
Strongylocentrotus purpuratus	Pacific purple sea urchin	XP_030854208
PARP4-like		
Homo sapiens	human	NP_006428
Notamacropus eugenii	Tammar wallaby	XP_072467755
Gallus gallus	chicken	NP_001164635
Xenopus laevis	African clawed frog	XP_041440456
Rhincodon typus	Whale shark	XP_048454258
Plantae		
PARP1-like		
Amborella trichopoda		XP_011624679
Arabidopsis thaliana	mouse-ear cress	NP_850165
Oryza glaberrima	African rice	XP_052161742
Lathyrus oleraceus[ii]	garden pea	XP_050910876
Zea mays	maize	NP_001288538
PARP3-like		
Arabidopsis thaliana	mouse-ear cress	NP_001318618
Nelumbo nucifera	sacred lotus	XP_010271692
Oryza glaberrima	African rice	XP_052145480
Lathyrus oleraceus[ii]	garden pea	PX437880
Zea mays	maize	XP_008645748

(i) GenBank accession number

(ii) formerly *Pisum sativum*

Supplementary Table 6. *Af*-PARP1 NLS prediction results.

tool	motif[i,ii]	score	reference
LOCALIZER 1.0.4	133-KRKL-136	n.a.	[12]
NLSExplorer	131-TNKRKL-136	0.99284 (2.25163)[iii]	[13]
	159-ANKKQKA-165	0.99148 (1.84237)[iii]	
cNLS Mapper	129-PKTNKRKLAD-138	4[iv]	[14]
DeepLoc 2.1	129-PKTNKRKLA-137[v]	0.8184[vi]	[15]
	157-DEANKKQKA-165[v]		

(i) Numbers indicate first and last position in sequence
(ii) Identified common motif is underlined
(iii) recommendation score (entropy score)
(iv) Score of 3-5 indicates localisation to both nucleus and cytoplasm
(v) Derived from logo-like attention plot output
(vi) Overall localisation probability score for 'nucleus' (threshold 0.5014); no other localisation probability above threshold in prediction

Supplementary Notes

Supplementary Note 1. Edhen's Minimal Medium for *Aspergillus* (EMMA) composition and preparation.

Base medium

reagent	for 1 L amount (g)	c [mM]
MES (free acid)	4.88	25
D-Glucose	10	55.5
K_2HPO_4	1.74	10
NH_4Cl	1.34	25
K_2SO_4	0.174	1
NaCl	0.058	1
$MgCl_2 \cdot 6\ H_2O$	0.407	2
$CaCl_2 \cdot 2\ H_2O$	0.074	0.5

- Dissolve in 900 mL dH_2O
- Add the following solutions:

Stock solution	V (µL)	c(final)
biotin stock (100 µg/mL)	100	0.01 µg/mL
1,000× Trace Elements	1000	various (see below)
100 mM $(NH_4)_2Fe(SO_4)_2$ [i]	300	30 µM

(i) Keep aliquoted at -20 °C

- OPTIONAL: For solid growth medium add 18 g/L purified agar
- Bring to 1 L final volume
- Sterilise by autoclaving (121 °C for 15 min)

1,000× Trace Elements

reagent	for 100 mL amount (g)	c(stock) [mM]	c(final) [µM]
$Na_2EDTA \cdot 2\ H_2O$	4.9	131.6	131.6
$ZnSO_4 \cdot 7\ H_2O$	2.2	76.5	76.5
H_2BO_3	1.1	178	178
$MnCl_2 \cdot 4\ H_2O$	0.5	25.25	25.25
$CoCl_2 \cdot 6\ H_2O$	0.172	7.2	7.2
$CuSO_4 \cdot 5\ H_2O$	0.16	6.4	6.4
Na_2MoO_4	0.15	6.2	6.2

- Dissolve in dH_2O the above order ensuring that the added salt has completely dissolved before adding the next one

Variation for post-transformation regeneration medium

- Add 342.3 g/L sucrose (final 1 M) to base medium
- Add 18 g/L purified agar for growth plates
- Add 7.5 g/L purified agar for overlay medium
- Autoclave as above
- Add appropriate selection agent into autoclaved and cooled (≤ 50 °C)

Supplementary Note 2. Codon-optimised coding and translated protein sequence of *Af*-PARP1.

>Af-PARP1 ORF
ATGGCACCGCGTACCTTCAAAAAAGCCGTTATTGCAGTTAGCGGCACCTTTAGCGGTTATAAACAGGC
AGATCTGAAAGCAATTGTTGAAGGTCAGGGTGCAACCTTTAGCACCACCGTTAGCGAAGGTTGTACCC
ATCTGGTTACCACACAGAAAGAAGTTGAAAAAGGCACCGTGAAATACAAACAGGCATGTAATGTTCCG
GGTTGTGATGTTGTTAGCCTGGAATGGCTGCTGGATAGCCAGAAAGCAGGTAAACCGCTGGCAAGCAA
AAGCTATTTTCTGACCGCAGGCGCAGCAAGCGTTGGTCAGGGTCAGAATGTTGATGGCACCGGCACCG
GTACAGCCCCTGCACCGGCAAGTGGTGCAACCACCGCAACCGCACCGAAAACCAATAAACGTAAACTG
GCAGATGCAAACGATGATACCGCAGGTAGCGCAGTTGATGCAAATGGTGCAAGCAATGGTGATGAAGC
AAACAAAAAACAGAAAGCCGCACAGAAAGCAAGCACCAAAAGCCTGAATGTGCCGGTTGATGAAGGCT
GTACCATGCCTGGTGCAGTTTTTATTGAAGATAGCGGTCTGATTTGGGATGCAACCCTGAATCAGACC
AATAGCGCACATAATAACAACAAGTTTTATCGCATTCAGCTGCTGGTTGATACCAGCGGCACCACCTA
TAAAACCTGGACACGTTGGGGTCGTGTTGGTGAATTTGGTAGCAGCGCACTGTTAGGTGATGGTACAC
TGCAGCGTGCAAAATTTGAATTTGAGAAGAAGTTCAAGGACAAAACCGGTCTGAAATGGGAAGATCGT
CTGAGCACCCCGAAAAGCAACAAATATACCTTTATCGAGCGCAACTATGAAGAAAGCGACGATGAAGA
TGATAGCGAGGATAAAATGGATGTGGATAAACGCGAAAAAATCGAAAGCACCCTGCCGCAGGCAGTTC
AAGAACTGCTGACCTTTATCTTTAATCAGCAGCATTTTCTGAGCACAATGGCAAGCATGAGCTATGAT
GCACAGAAACTGCCGCTGGGTAAACTGAGCAAACGTACCCTGACTACCGGTTTTCAGATCCTGAAAGA
TCTGAGCGAACTGGTTGCAGATCCGAGCCTGGCCAGCAGCCGTTATAATACCAGCTTTCAGCAGGCAG
CACTGGATCTGAGCAATCGTTATTTTACCACCATTCCGCATGTGTTTGGTCGTAATACCCCTCCGATT
CTGACCAAAGATCATCAGATTAAAACCGAAGTTGATCTGCTGGAAGCACTGACCGATATGGGTGTTGC
CAATGGTATTATGAAAAATAGCCGTGATGCCGAGATGATGAATCAGCTGGATCGTCAGTATCAAGGTC
TGGGTATGCAAGAAATGACACCGCTGAATCGTAGCAGTGCAGAATTTCGTGAACTGGAAGCATATCTG
AATAATAGTCGTGGTAGCACCCATCATCTGCGTTTTAGCGTTATTAACATCTTTCGCATTGAACGCAA
AGGTGAAGAAGATCGTTTTAAGAGCAGCTGTTTTGCCAATCTGACCAATTCAAATCGTCGTCTGCTGT
GGCATGGTAGCCGTAGCACCAATTTTGGTGGTATTCTGAGCCAGGGTCTGCGTATTGCACCGCCTGAA
GCACCGGTTAGTGGTTATATGTTTGGTAAAGGTGTGTATTTCGCAGACATGAGCAGCAAAAGCGCAAA
TTATTGTGTTCCGTATAATAGCGCAAATATGGGTCTGCTGCTGCTGTGCGACGTTGAACTGGGTAATC
CGATGCTGGAACAGTTTCGTGCAAACTTTAATGCAGGCACCGATGCAAAAGCACAGGGTAAAATTGCA
ACCTTAGGTAAAGGTCGTACCATTCCGGCAGGTTGGAAAGATGCAGGTTGTCTGCATCCGGCACTGAG
CGGTGTTCAGATGCCAGATGTTAGCAAAAGTACCATGAGCGATAATGGTAGCCTGCTGTATAACGAGT
ATATCGTTTATGATGTTGCCCAGATTCGTCAGCGTTACCTGTTTCATGTTCACATGAGCTAA

>Af-PARP1 protein sequence
MAPRTFKKAVIAVSGTFSGYKQADLKAIVEGQGATFSTTVSEGCTHLVTTQKEVEKGTVKYKQACNVP
GCDVVSLEWLLDSQKAGKPLASKSYFLTAGAASVGQGQNVDGTGTGTAPAPASGATTATAPKTNKRKL
ADANDDTAGSAVDANGASNGDEANKKQKAAQKASTKSLNVPVDEGCTMPGAVFIEDSGLIWDATLNQT
NSAHNNNKFYRIQLLVDTSGTTYKTWTRWGRVGEFGSSALLGDGTLQRAKFEFEKKFKDKTGLKWEDR
LSTPKSNKYTFIERNYEESDDEDDSEDKMDVDKREKIESTLPQAVQELLTFIFNQQHFLSTMASMSYD
AQKLPLGKLSKRTLTTGFQILKDLSELVADPSLASSRYNTSFQQAALDLSNRYFTTIPHVFGRNTPPI
LTKDHQIKTEVDLLEALTDMGVANGIMKNSRDAEMMNQLDRQYQGLGMQEMTPLNRSSAEFRELEAYL
NNSRGSTHHLRFSVINIFRIERKGEEDRFKSSCFANLTNSNRRLLWHGSRSTNFGGILSQGLRIAPPE
APVSGYMFGKGVYFADMSSKSANYCVPYNSANMGLLLLCDVELGNPMLEQFRANFNAGTDAKAQGKIA
TLGKGRTIPAGWKDAGCLHPALSGVQMPDVSKSTMSDNGSLLYNEYIVYDVAQIRQRYLFHVHMS*

Supplementary Note 3. Codon-optimised coding and translated protein sequence of V5-mScarlet-I3.

>**V5**-mScarlet-I3 ORF
ATGGCC**GGTAAGCCCATCCCCAACCCCCTGCTGGGTCTGGACTCCACC**GGTTCCGCCGGTTCCGCCGG
TATGGACTCCACCGAGGCCGTCATCAAGGAGTTCATGCGCTTCAAGGTCCACATGGAGGGTTCCATGA
ACGGTCACGAGTTCGAGATTGAGGGTGAGGGTGAGGGTCGCCCCTACGAGGGTACCCAGACCGCCAAG
CTGAAGGTCACCAAGGGTGGTCCCCTGCCCTTCTCCTGGGACATCCTGTCCCCCCAGTTCATGTACGG
TTCCCGCGCCTTCATCAAGCACCCCGCCGACATCCCCGACTACTGGAAGCAATCCTTCCCGGAGGGCT
TCAAGTGGGAGCGTGTCATGATCTTCGAGGACGGTGGTACCGTCTCCGTCACCCAGGACACCTCCCTG
GAGGACGGTACCCTGATCTACAAGGTCAAGCTGCGCGGTGGTAACTTCCCCCCCGACGGTCCCGTCAT
GCAGAAGCGCACTATGGGTTGGGAGGCCTCCACCGAGCGCCTGTACCCCGAGGACGTCGTCCTGAAGG
GTGACATCAAGATGGCCCTGCGCCTGAAGGACGGTGGTCGCTACCTGGCCGACTTCAAGACCACCTAC
AAGGCCAAGAAGCCCGTCCAGATGCCCGGTGCCTTCAACATCGACCGCAAGCTGGACATCACCTCCCA
CAACGAGGACTACACCGTCGTCGAGCAGTACGAGCGCTCCGTCGCCCGCCACTCCACCGGTGGTTCCG
GTGGTTCC

>**V5**-mScarlet-I3 protein sequence
MA**GKPIPNPLLGLDST**GSAGSAGMDSTEAVIKEFMRFKVHMEGSMNGHEFEIEGEGEGRPYEGTQTAK
LKVTKGGPLPFSWDILSPQFMYGSRAFIKHPADIPDYWKQSFPEGFKWERVMIFEDGGTVSVTQDTSL
EDGTLIYKVKLRGGNFPPDGPVMQKRTMGWEASTERLYPEDVVLKGDIKMALRLKDGGRYLADFKTTY
KAKKPVQMPGAFNIDRKLDITSHNEDYTVVEQYERSVARHSTGGSGGS

Supplemental References

1 Obaji, E., Haikarainen, T. & Lehtio, L. Structural basis for DNA break recognition by ARTD2/PARP2. *Nucleic Acids Res* **46**, 12154-12165 (2018). https://doi.org:10.1093/nar/gky927

2 Frigon, L. & Pascal, J. M. Structural and biochemical analysis of the PARP1-homology region of PARP4/vault PARP. *Nucleic Acids Res* **51**, 12492-12507 (2023). https://doi.org:10.1093/nar/gkad1064

3 Patel, M. R. *et al.* Discovery and structure-activity relationship of novel 2,3-dihydrobenzofuran-7-carboxamide and 2,3-dihydrobenzofuran-3(2H)-one-7-carboxamide derivatives as poly(ADP-ribose)polymerase-1 inhibitors. *J Med Chem* **57**, 5579-5601 (2014). https://doi.org:10.1021/jm5002502

4 Bilokapic, S., Suskiewicz, M. J., Ahel, I. & Halic, M. Bridging of DNA breaks activates PARP2-HPF1 to modify chromatin. *Nature* **585**, 609-613 (2020). https://doi.org:10.1038/s41586-020-2725-7

5 Frigon, L. & Pascal, J. M. Crystal structure and mutagenesis of a nucleic acid-binding BRCT domain in human PARP4. *J Biol Chem* **301**, 110277 (2025). https://doi.org:10.1016/j.jbc.2025.110277

6 Biostrings: Efficient manipulation of biological strings. v. 2.76.0 (2025).

7 Babicki, S. *et al.* Heatmapper: web-enabled heat mapping for all. *Nucleic Acids Res* **44**, W147-153 (2016). https://doi.org:10.1093/nar/gkw419

8 Dolinsky, T. J. *et al.* PDB2PQR: expanding and upgrading automated preparation of biomolecular structures for molecular simulations. *Nucleic Acids Res* **35**, W522-525 (2007). https://doi.org:10.1093/nar/gkm276

9 Dolinsky, T. J., Nielsen, J. E., McCammon, J. A. & Baker, N. A. PDB2PQR: an automated pipeline for the setup of Poisson-Boltzmann electrostatics calculations. *Nucleic Acids Res* **32**, W665-667 (2004). https://doi.org:10.1093/nar/gkh381

10 Baker, N. A., Sept, D., Joseph, S., Holst, M. J. & McCammon, J. A. Electrostatics of nanosystems: application to microtubules and the ribosome. *Proc Natl Acad Sci U S A* **98**, 10037-10041 (2001). https://doi.org:10.1073/pnas.181342398

11 Baker, E. S. & Bowers, M. T. B-DNA helix stability in a solvent-free environment. *J Am Soc Mass Spectrom* **18**, 1188-1195 (2007). https://doi.org:10.1016/j.jasms.2007.03.001

12 Sperschneider, J. *et al.* LOCALIZER: subcellular localization prediction of both plant and effector proteins in the plant cell. *Sci Rep* **7**, 44598 (2017). https://doi.org:10.1038/srep44598

13 Li, Y. F., Pan, X. & Shen, H. B. Discovering the nuclear localization signal universe through a deep learning model with interpretable attention units. *Patterns (N Y)* **6**, 101262 (2025). https://doi.org:10.1016/j.patter.2025.101262

14 Kosugi, S., Hasebe, M., Tomita, M. & Yanagawa, H. Systematic identification of cell cycle-dependent yeast nucleocytoplasmic shuttling proteins by prediction of composite motifs. *Proc Natl Acad Sci U S A* **106**, 10171-10176 (2009). https://doi.org:10.1073/pnas.0900604106

15 Odum, M. T. *et al.* DeepLoc 2.1: multi-label membrane protein type prediction using protein language models. *Nucleic Acids Res* **52**, W215-W220 (2024). https://doi.org:10.1093/nar/gkae237
