# Supplementary material for: The BRCT domain enhances DNA binding and catalytic efficiency of fungal PARPs
Source: Nat Commun. 2026 Jul 24;17:9024. doi: 10.1038/s41467-026-75844-6 (PMC13507078; doi:10.1038/s41467-026-75844-6)
Supplement: Supplementary file 2 [file 41467_2026_75844_MOESM2_ESM.pdf]

nature portfolio

Corresponding author(s): Johannes G. M. Rack
Last updated by author(s): Jun 30, 2026

Reporting Summary

Nature Portfolio wishes to improve the reproducibility of the work that we publish. This form provides structure for consistency and transparency in reporting. For further information on Nature Portfolio policies, see our Editorial Policies and the Editorial Policy Checklist.

Statistics

For all statistical analyses, confirm that the following items are present in the figure legend, table legend, main text, or Methods section.

n/a	Confirmed	
☐	☒	The exact sample size (*n*) for each experimental group/condition, given as a discrete number and unit of measurement
☐	☒	A statement on whether measurements were taken from distinct samples or whether the same sample was measured repeatedly
☐	☒	The statistical test(s) used AND whether they are one- or two-sided<br>*Only common tests should be described solely by name; describe more complex techniques in the Methods section.*
☒	☐	A description of all covariates tested
☒	☐	A description of any assumptions or corrections, such as tests of normality and adjustment for multiple comparisons
☐	☒	A full description of the statistical parameters including central tendency (e.g. means) or other basic estimates (e.g. regression coefficient) AND variation (e.g. standard deviation) or associated estimates of uncertainty (e.g. confidence intervals)
☐	☒	For null hypothesis testing, the test statistic (e.g. *F*, *t*, *r*) with confidence intervals, effect sizes, degrees of freedom and *P* value noted<br>*Give P values as exact values whenever suitable.*
☒	☐	For Bayesian analysis, information on the choice of priors and Markov chain Monte Carlo settings
☒	☐	For hierarchical and complex designs, identification of the appropriate level for tests and full reporting of outcomes
☒	☐	Estimates of effect sizes (e.g. Cohen's *d*, Pearson's *r*), indicating how they were calculated

Our web collection on statistics for biologists contains articles on many of the points above.

Software and code

Policy information about availability of computer code

Data collection	Image Lab (OS of ChemiDoc System, Bio-RAD)<br>CDApps (CD data acquisition; PMID: 25723950)<br>General Data Acquisition software (GDA; DLS) and DAWN Science (DLS) for SAXS data acquisition<br>CellSense APX software (v.4.4)<br>Andor Fusion software (v.2.6)
Data analysis	Prism (v 10.6.1, GraphPad)<br>Fiji/ImageJ (v 1.54p) with BIOP JACoP plugin (accessed February 2026)<br>ScÅtter IV (Bioisis)<br>Cellpose (v. 4.0.8)<br>JalView v. 2.11<br>MEGA11<br>ALINE (v. 1.0.025)<br>R with Biostrings package<br>heatmapper

For manuscripts utilizing custom algorithms or software that are central to the research but not yet described in published literature, software must be made available to editors and reviewers. We strongly encourage code deposition in a community repository (e.g. GitHub). See the Nature Portfolio guidelines for submitting code & software for further information.

Data

Policy information about availability of data

All manuscripts must include a data availability statement. This statement should provide the following information, where applicable:

- Accession codes, unique identifiers, or web links for publicly available datasets
- A description of any restrictions on data availability
- For clinical datasets or third party data, please ensure that the statement adheres to our policy

The partial mRNA sequence of LoPARP2 and coding DNA sequence of LoPARP3 were deposited in GenBank under the accession number PX314872 and PX437880, respectively.
SAXS profiles and pair distribution functions were uploaded to the SASBDB and are available under the accession codes SASDXE9 (Af-PARP1 apo), SASDXF9 (Af-PARP1 ΔBRCT apo), SASDXG9 (Af-PARP1:DNA-1), and SASDXH9 (Af-PARP1 ΔBRCT:DNA-1).
AlphaFold 3 models were deposited to ModelArchive and are available under the model IDs ma-wejsd [https://modelarchive.org/doi/10.5452/ma-wejsd] (Af-PARP1:DNA-1:NAD+) and ma-8ypox [https://modelarchive.org/doi/10.5452/ma-8ypox](Af-PARP1:NAD+).
All other data generated in this study are provided in the Supplementary Information and Source Data files.

Research involving human participants, their data, or biological material

Policy information about studies with human participants or human data. See also policy information about sex, gender (identity/presentation), and sexual orientation and race, ethnicity and racism.

Reporting on sex and gender	n/a
Reporting on race, ethnicity, or other socially relevant groupings	n/a
Population characteristics	n/a
Recruitment	n/a
Ethics oversight	n/a

Note that full information on the approval of the study protocol must also be provided in the manuscript.

Field-specific reporting

Please select the one below that is the best fit for your research. If you are not sure, read the appropriate sections before making your selection.

☒ Life sciences ☐ Behavioural & social sciences ☐ Ecological, evolutionary & environmental sciences

For a reference copy of the document with all sections, see nature.com/documents/nr-reporting-summary-flat.pdf

Life sciences study design

All studies must disclose on these points even when the disclosure is negative.

Sample size	Sample size calculation was not performed. For Galleria mellonella infection study experiments were performed in quadruplicate with n=10 individuals per sample group. The number of individuals per group was based on previous reports and observations.<br>For co-localisation analysis, z-stacks of n=18 (WT and K60A mutant) or n=17 (dBRCT2) nuclei were collected. For CD data, data were collected from single dilution series. For all other experiments involving statistical analysis experiments were performed as three biological replicas and where possible in triplicates to ensure consistence within experiments.<br><br>For experiments not involving statistical analysis, experiments were performed in biological triplicates to ensure consistence within the experiments.
Data exclusions	No data were excluded.
Replication	Experiments were performed as biological duplicates or triplicates as indicated.
Randomization	Samples were not divided into experimental groups, all replicates for individual experiments were simultaneously prepared, handled, and statistically processed while taking multiple-hypotheses testing into account.
Blinding	All data analysis was performed with unbiased software, in an unsupervised manner, and therefore blinding is not applicable in this context.

Reporting for specific materials, systems and methods

We require information from authors about some types of materials, experimental systems and methods used in many studies. Here, indicate whether each material, system or method listed is relevant to your study. If you are not sure if a list item applies to your research, read the appropriate section before selecting a response.

Materials & experimental systems

n/a	Involved in the study
☐	☒ Antibodies
☐	☒ Eukaryotic cell lines
☒	☐ Palaeontology and archaeology
☐	☒ Animals and other organisms
☒	☐ Clinical data
☒	☐ Dual use research of concern
☐	☒ Plants

Methods

n/a	Involved in the study
☒	☐ ChIP-seq
☒	☐ Flow cytometry
☒	☐ MRI-based neuroimaging

Antibodies

Antibodies used

poly/mono-ADP-ribose (clone E6F6A [RRID:AB_2749858] or D9P7Z [RRID:AB_3716623], Cell Signalling Technology)
PAR/pADPr (R and D Systems Cat# 4335-MC-100, RRID:AB_3644339)
mono-ADP-Ribose antibody (Bio-Rad Cat# HCA354, RRID:AB_3720089)
anti-pan-ADP-ribose (Millipore Cat# MABE1016, RRID:AB_2665466)
anti-poly-ADP-ribose (Millipore Cat# MABE1031, RRID:AB_2665467)
anti-mono-ADP-ribose (Millipore Cat# MABE1076, RRID:AB_2665469)
horseradish peroxidase-labelled anti-rabbit IgG (Agilent Cat# P0399, RRID:AB_2617141)
horseradish peroxidase-labelled anti-mouse IgG (Agilent Cat# P0447, RRID:AB_2617137)
HRP-conjugated streptavidin (Millipore Cat. #18-152)

Validation

Commercial antibodies were validated by the Manufacturer for their application in immunoblotting:
poly/mono-ADP-ribose (clone E6F6A [RRID:AB_2749858] or D9P7Z [RRID:AB_3716623], Cell Signalling Technology)
https://www.cellsignal.com/products/primary-antibodies/poly-mono-adp-ribose-e6f6a-rabbit-monoclonal-antibody/83732
https://www.cellsignal.com/products/primary-antibodies/poly-mono-adp-ribose-d9p7z-rabbit-monoclonal-antibody/89190

PAR/pADPr (R and D Systems Cat# 4335-MC-100, RRID:AB_3644339)
https://www.rndsystems.com/products/par-padpr-antibody-10ha_4335-mc-100

mono-ADP-Ribose antibody (Bio-Rad Cat# HCA354, RRID:AB_3720089)
https://www.bio-rad-antibodies.com/monoclonal/human-mono-adp-ribose-antibody-abd33204-hca354.html

anti-pan-ADP-ribose (Millipore Cat# MABE1016, RRID:AB_2665466)
https://www.sigmaaldrich.com/GB/en/product/mm/mabe1016

anti-poly-ADP-ribose (Millipore Cat# MABE1031, RRID:AB_2665467)
https://www.sigmaaldrich.com/GB/en/product/mm/mabe1031

anti-mono-ADP-ribose (Millipore Cat# MABE1076, RRID:AB_2665469)
https://www.sigmaaldrich.com/GB/en/product/mm/mabe1076

horseradish peroxidase-labelled anti-rabbit IgG (Agilent Cat# P0399, RRID:AB_2617141)
https://www.agilent.com/en/product/specific-proteins/elisa-kits-accessories/swine-anti-rabbit-immunoglobulins-hrp-affinity-isolated-2717118

horseradish peroxidase-labelled anti-mouse IgG (Agilent Cat# P0447, RRID:AB_2617137)
https://www.agilent.com/en/product/specific-proteins/elisa-kits-accessories/goat-anti-mouse-immunoglobulins-hrp-affinity-isolated-2717109

HRP-conjugated streptavidin (Millipore Cat. #18-152)
https://www.sigmaaldrich.com/GB/en/product/mm/18152

Eukaryotic cell lines

Policy information about cell lines and Sex and Gender in Research

Cell line source(s)	murine 2H11 (CRL-2163, ATCC)
Authentication	Cell line showed normal growth behavior, but was not individually authenticated

Mycoplasma contamination	not tested mycoplasma
Commonly misidentified lines (See ICLAC register)	*Name any commonly misidentified cell lines used in the study and provide a rationale for their use.*

Animals and other research organisms

Policy information about studies involving animals; ARRIVE guidelines recommended for reporting animal research, and Sex and Gender in Research

Laboratory animals	n/a
Wild animals	Galleria mellonella was purchased from Livefood UK Ltd, Somerset, UK
Reporting on sex	no sex data were collected
Field-collected samples	n/a
Ethics oversight	no ethical approval needed

Note that full information on the approval of the study protocol must also be provided in the manuscript.

Dual use research of concern

Policy information about dual use research of concern

Hazards

Could the accidental, deliberate or reckless misuse of agents or technologies generated in the work, or the application of information presented in the manuscript, pose a threat to:

No	Yes	
☒	☐	Public health
☒	☐	National security
☒	☐	Crops and/or livestock
☒	☐	Ecosystems
☒	☐	Any other significant area

Experiments of concern

Does the work involve any of these experiments of concern:

No	Yes	
☒	☐	Demonstrate how to render a vaccine ineffective
☒	☐	Confer resistance to therapeutically useful antibiotics or antiviral agents
☒	☐	Enhance the virulence of a pathogen or render a nonpathogen virulent
☒	☐	Increase transmissibility of a pathogen
☒	☐	Alter the host range of a pathogen
☒	☐	Enable evasion of diagnostic/detection modalities
☒	☐	Enable the weaponization of a biological agent or toxin
☒	☐	Any other potentially harmful combination of experiments and agents

Plants

Seed stocks	Seeds of Lathyrus oleraceus (formerly Pisum sativum) cultivar 'Blauwschokker' were purchased from Premier Seeds Direct Ltd, Wilton, UK (batch PL530/12/210/L68/A)
Novel plant genotypes	n/a
Authentication	n/a
