# Supplementary material for: The BRCT domain enhances DNA binding and catalytic efficiency of fungal PARPs
Source: Nat Commun. 2026 Jul 24;17:9024. doi: 10.1038/s41467-026-75844-6 (PMC13507078; doi:10.1038/s41467-026-75844-6)
Supplement: Supplementary file 3 [file 41467_2026_75844_MOESM3_ESM.pdf]

nature portfolio

Peer Review File

The BRCT domain enhances DNA binding and catalytic efficiency of fungal PARPs

Corresponding Author: Dr Johannes Rack

This file contains all reviewer reports in order by version, followed by all author rebuttals in order by version.

Version 0:

Reviewer comments:

Reviewer #1

(Remarks to the Author)
In this work submitted to Nature Communications, Bainbridge and colleagues investigate fungal PARP1 homologs. They focus in particular on the BRCT domain of this protein and determine how this domain contributes to regulation of PARP activity. The authors purify PARP1 wildtype and mutant proteins and test DNA binding as well as PARP catalytic activity. The work is not highly novel or original, as it is known that PARP1 (of many species) is activated by DNA damage, mediated by DNA binding of several domains. Considering the considerable similarity between AfPARP1 (BRCT, WGR, PARP domain even if lacking ZnFs), it is not very surprising that also AfPARP1 is activated by DNA damage. Like the human PARP1 BRCT domain, the Af BRCT also binds to undamaged DNA and similar to human PARP1, may assist in recruiting the protein to sites of damage. In contrast to human PARP1, the BRCT domain may be required for full PARP1 activity, although this claim is not fully supported by the data. This would be a novel aspect of this work, which will have to be strengthened.

Major comments
Figure 2a. Can the authors comment on the clear mobility shift in the CB? Would this imply that the enzyme functions mostly as monoPARP, as I would expect PAR-forming PARPs to lead to a smear? The authors should probe these blots with antibodies specific for mono or polyADPribosylation (such as available from BioRad for MAR and BioTechne for PAR) to distinguish between these two possibilities. In fact, I would recommend to do this for all blots to determine whether the wildtype enzyme and mutants generate MAR, PAR or a mix of both. As the authors mention no HPF is present in fungi; perhaps the BRCT1 domain can shift activity between short/long polymers?

I am not convinced with the difference in PARP1 activation in Figure 6, which could perhaps be attributed to slight differences in protein amounts used – to my eye it seems like more PARP1 wt was used (intensity CBB stainings). Furthermore, even if we agree that in very early timepoints the mutants do not show activity like the wildtype, the question becomes whether this slightly slower kinetic has any biological impact? The PAR is still formed, albeit slightly slower. To increase impact of this work, authors should consider some simple biological tests such as recruitment to DNA damage. Is this impaired?
Furthermore, the experiment A seems to be add odds with the NAD+ conversion assay in C: in A, the signal still increases after 10 minutes whereas in C the NAD+ is almost immediately used up. The authors should investigate the cause of this discrepancy.

It appears that AfPARP1 may behave more like human PARP3 than human PARP1, if it truly does not generate the “smeared” PAR chains that PARP1 makes. It might be worth to investigate this in more detail. Here it would also be of interest to determine in more detail what is modified by AfPARP1 and whether the BRCT domain changes this? The neutral hydroxylamine treatment would at 4 hours remove from more residues than just glutamate. This should be easy to test using specific hydrolyzing enzymes and the advantage here would be that the authors can also probe for MAR vs PAR using the right enzymes.

Lastly, I would recommend to downtune some statements, as an example: “Given the molecular divergence from the mammalian pathway observed in our data, this signalling pathway emerges as a promising target for antifungal therapy.” Presumably, the PARP catalytic domains are highly similar – assumed from usage of an inhibitor developed for human PARPs (talazoparib). Also in human PARPs, the BRCT domain is important for DNA binding (Rudolph et al, Mol Cell 2021).

This statement should be reconsidered, as (a) the current study does not address at all whether PARP1 is essential for fungi and (b) it is not that obvious that specifically targeting fungal PARP activity will be that easily achievable.

Minor comments

Line 206: the cited paper from the Luger lab showed that the BRCT domain binds intact DNA and proposed an intriguing model for its functon(contributing to monkeybar mechanism). Considering the high relevance of this publication to the work submitted, the authors should expand more on what has been done in this prior work on the human BRCT domain. This is done in the discussion, but would fit earlier in the manuscript.

In tests of catalytic activity, 500µM NAD is used. Is NAD available in the model organism at such high levels? How would catalytic activity look at lower, presumably more physiological, concentrations?

Figure 2a: it is not clear what “activated DNA” is and how this compares to the DNA oligonucleotides used in later figures.

Figure 2: it would be interesting to have a direct comparison with human PARP1 (which is commercially available). The “PAR” formed here appears to be rather shorter than PAR chains typically observed after hsPARP1 reactions.

Figure 6a: what are the distinct bands that are appearing, especially for example in PARP1delBRCT t=2 minutes, an interesting pattern appears in CBB which is not reflected in immunoblotting?

Model: would be good to also draw the human PARP1 to show the proposed difference between both systems

Sequence alignment extended figure 6: lack of conservation on sequence level doesn't mean there is no structural conservation.

The methods section is lacking details in the plasmid construction paragraph. It is not clear exactly how constructs were generated, and how for example the codon optimization looks. This would be essential to be able to reproduce this work.

There are many typos, especially in the figure legends.

Reviewer #2

(Remarks to the Author)

Bainbridge and colleagues report their analysis of fungal PARP enzymes and the contribution of the BRCT domain to DNA binding and catalytic activation of the PARP enzyme from A. fumigatus, a fungus considered a health concern. The study presents a phylogenetic analysis of PARP enzymes in the fungus kingdom, and then focus biochemical and structural work primarily on an A. fumigatus PARP that is expected to respond to DNA damage. They show that Af-PARP1 is activated by DNA damage of certain types, in particular DNA breaks bearing 5 prime phosphates, and also 3 prime DNA overhangs. The study identifies the BRCT domain of Af-PARP1 as contributing to the DNA interaction, and also the activation mechanism that leads to ADP-ribose modifications. The biochemical analysis is supported by predicted structures and experimental structures of homologous human PARPs. The study compares and contrasts Af-PARP with human homologs for which there is similar biochemical data. The study is clearly presented and the data is well done for the most part. There are a few areas that could be further developed to broaden the investigation and perhaps give deeper insights.

Ext. Data Figure 4e. The CD titration to quantitatively determine DNA binding affinity values needs more description in the methods, and it is not clear that the data is providing an accurate assessment of affinity. What are the units of the values in Figure 4e? The methods used uM and mM units, so it needs to be specified in the table. It is stated that DNAs were diluted to 14.5 uM and ligand (PARP protein) concentration was 0.269, 0.346, 0.336, or 0.433 mM depending on the ligand. It is thus not clear what was being titrated, since fixed values are given for both DNA and ligand. Ratios are given that indicate that protein was titrated, so it would seem that the mM concentrations given are simply the stock that was used, but this needs to be clarified. If the values in panel 4e are uM, then it does not seem that the titration is well-suited to accurately measure a KD, and it is clear that most data points are above the determined KD. The legend states that a Hill equation was used, but it seems that a more simple 1:1 binding model should be used base on the other analysis. What were the n values from the fit? A quantitative analysis of the binding affinities is important, in particular for comparing the contribution of the BRCT domain relative to the rest of Af-PARP2, but it is not clear that this assay is doing a good job. Is there a second assessment that can be used for quantification, like the EMSA assays? If the CD assay is used, it needs to be better developed and explained.

Is it expected that Af-PARP1 will employ the same allosteric activation mechanism defined for human PARPs 1, 2, and 3? And if so, are there mutants or assays that could support this prediction? It is not clear what to expect from the AlphaFold predictions that are provided.

Does Af-PARP1 inactivate itself through self-modification (diminished DNA binding), similar to other DNA activated PARPs? And does the BRCT domain interact with ADP-ribose or poly-ADP-ribose?

Does Af-PARP1 produce mono or poly-ADP-ribose modifications? I don't think this was addressed, but the final model lists PAR and not MAR as the signal produced. Addressing this experimentally would be useful for understanding this system.

The sequence analysis of the BRCT and WGR domains should be extended to the catalytic region (HD and ART).

What is the significance of the binding to overhang DNA? Is there a particular length that is ideal for the overhang? Was binding to single-strand DNA tested?

CBB acronym is not defined anywhere that I could find.

Reviewer #3

(Remarks to the Author)
The paper provides an evolutionary analysis of the fungal PARPs and their domain architecture and then focuses on Af-PARP1, a homolog of human PARP1/2. They use EMSA and activity assays to show that Af-PARP1 is catalytically responsive to DNA damage but despite its closer relation with animal and plant PARP2, it binds DNA and is activated like animal PARP3. They investigate the Af-PARP1 WGR domain (present in human PARP1, 2, and 3) and show that Af-PARP1 (unlike human PARP2) is not activated by blunt ends despite high conservation of their WGR domains. Conversion of the single non-conserved residue in Af-PARP1 WGR to that of human PARP2 did not affect its binding to blunt ends. Additional mutational analysis showed that Af-PARP1 has a 5'-phosphate sensing mechanism conserved across zinc finger-independent PARPs like human PARP2 and 3. They next investigate the Af-BRCT domain (present in human PARP1 not PARP2 or 3) and show that deletion severely restricts the DNA-binding profile. SAXS analysis shows that upon DNA binding, the BRCT domain transitions from behaving as an independent domain to forming an integral part of the multidomain WGR-HD-ART assembly, a behavior that contrasts with that of human PARP1. Time course analysis shows Af-PARP WT is activated by both nicked and gapped DNA, whereas ΔBRCT is activated by nicked DNA but reduced by gap DNA. Lysine mutations in BRCT retained the ability to bind nicked DNA but showed reduced binding and activation by gap DNA. Further analysis of one mutant, K52/60A, showed it behaved like WT in response to nicked DNA but was reduced in binding, activation, and NAD+ consumption in response to gapped DNA. Altogether showing that the BRCT domain can broaden the range of DNA damage structures recognized by Af-PARP1. They propose a model where the BRCT and WGR domains act cooperatively to scan and bind DNA and activate PARylation.

This is a clear study characterizing fungal PARPs. The comprehensive phylogenetic analysis should provide a roadmap for future studies of fungal PARPs. The in vitro data are of high quality and support the conclusions drawn. I have no concerns with the data other than a couple of minor technical points below. What is less clear is how this work will impact the field. The architecture of Af-PARP1, is not so much "unique" as it is just a different configuration of known motifs. The details of how the motifs influence recognition and binding to DNA damage differ from previous examples but not so dramatically as to have a great impact. For example, it is shown that the Af-BRCT domain contributes to the WGR-HD-ART assembly, whereas for human PARP1, the BRCT may not be involved. Since the concept of assembly has already been well-documented with human PARP1, this result is interesting but does not provide new insights. Insight into fungal PARPs in general could have important implications for antifungal therapy. However, it is not yet known if PARPs are important for fungal virulence. Thus overall, while this is a well-done interesting study, its descriptive nature and narrow focus may not appeal to a broad audience.

1. There is a discrepancy between the text in the Results section and the Figure (2):
Line 142 describes DNA binding "Fig. 2b" however the DNA binding is shown in Fig. 2c.
Line 155 describes catalytic activity "Fig. 2c" however catalytic activity is shown in Fig. 2b.
2. I cannot find a description in the text of the Bd-PARP K80A mutant shown in Fig. 5C.

Version 1:

Reviewer comments:

Reviewer #1

(Remarks to the Author)
The authors have responded well to the reviewers comments and suggestions. I do not have further remarks.

Reviewer #2

(Remarks to the Author)
In this resubmission, the authors have addressed the concerns that I raised during the initial review. They have added several experiments that make for a more complete description of the biochemistry of Af-PARP1.

minor comments:
I could not find the source of the compound BAD in the methods section.

Supplemental legends: please include primary publication references (if available) for PDB structures used in figures (supplementary figures 5c,g and 6a and 7e.)

Reviewer #3

(Remarks to the Author)

Open Access This Peer Review File is licensed under a Creative Commons Attribution 4.0 International License, which permits use, sharing, adaptation, distribution and reproduction in any medium or format, as long as you give appropriate credit to the original author(s) and the source, provide a link to the Creative Commons license, and indicate if changes were made.
In cases where reviewers are anonymous, credit should be given to 'Anonymous Referee' and the source.
The images or other third party material in this Peer Review File are included in the article’s Creative Commons license, unless indicated otherwise in a credit line to the material. If material is not included in the article’s Creative Commons license and your intended use is not permitted by statutory regulation or exceeds the permitted use, you will need to obtain permission directly from the copyright holder.
To view a copy of this license, visit https://creativecommons.org/licenses/by/4.0/

RESPONSE TO REVIEWER COMMENTS

Reviewer #1

In this work submitted to Nature Communications, Bainbridge and colleagues investigate fungal PARP1 homologs. They focus in particular on the BRCT domain of this protein and determine how this domain contributes to regulation of PARP activity. The authors purify PARP1 wildtype and mutant proteins and test DNA binding as well as PARP catalytic activity. The work is not highly novel or original, as it is known that PARP1 (of many species) is activated by DNA damage, mediated by DNA binding of several domains. Considering the considerable similarity between AfPARP1 (BRCT, WGR, PARP domain even if lacking ZnFs), it is not very surprising that also AfPARP1 is activated by DNA damage. Like the human PARP1 BRCT domain, the Af BRCT also binds to undamaged DNA and similar to human PARP1, may assist in recruiting the protein to sites of damage. In contrast to human PARP1, the BRCT domain may be required for full PARP1 activity, although this claim is not fully supported by the data. This would be a novel aspect of this work, which will have to be strengthened.

Major comments

Figure 2a. Can the authors comment on the clear mobility shift in the CB? Would this imply that the enzyme functions mostly as monoPARP, as I would expect PAR-forming PARPs to lead to a smear? The authors should probe these blots with antibodies specific for mono or polyADPribosylation (such as available from BioRad for MAR and BioTechne for PAR) to distinguish between these two possibilities. In fact, I would recommend to do this for all blots to determine whether the wildtype enzyme and mutants generate MAR, PAR or a mix of both. As the authors mention no HPF is present in fungi; perhaps the BRCT1 domain can shift activity between short/long polymers?

We thank the reviewer for their suggestion. To address this point, we expanded the characterisation of the *Af*-PARP1-derived signal using multiple MAR- and PAR-specific antibodies, a polymer-extension deficient mutant (E657Q), and a panel of (ADP-ribosyl)hydrolases (Revised Supplementary Fig.3).

We can confirm that the primary form of the modification is PARylation, most likely in the form of short polymers, although some MARylation may contribute to the observed signal. The short polymer length may explain the apparent upwards shift into a fuzzy band. Using the suggested antibodies, we observed a reduced signal when utilising MARylation specific antibody/detection reagent (Millipore MABE1076 and Bio-RAD HCA354), while clear staining was obtained for PARylation (utilising Millipore MABE1031 and R&D 4335-MC-100; Revised Supplementary Fig. 3a). We would like to note that some of the observed MARylation signal may be due to cross-reaction with the PAR signal, a problem known for the MABE1076 detection reagents (see e.g. Weixler, L., et al., Life Sci Alliance, 2022).
To further investigate polymer formation, we generated an E657Q mutation, which is analogues to the polymer extension deficient E998Q mutation of *h*PARP1. Modification of the mutant is only detected using the MARylation preferring antibodies, whereas the PARylation specific antibodies gave no signal. Moreover, analysis of ΔBRCT mutant revealed a

modification pattern indistinguishable from WT, indicating that the BRCT domain does not influence the balance between MARylation and PARylation.
In addition to the experimental analysis, we also include here a sequence analysis of the HD-ART domain. The ART domain D-loop lacks the characteristic features associated with MARyation in *h*PARP3 and instead more closely resembles the corresponding region of *h*PARP1/2 (see Supplementary Fig. 2).

While a detailed investigation of chain length regulation will be of considerable interest, we feel that repeating all catalytic assays using multiple antibody detection systems would extend beyond the scope of the current study. Nevertheless, our newly identified allosteric mutants revealed the formation of longer PAR chains upon relief of autoinhibition (see reviewer #2 comments), suggesting that chain length may itself be influenced by the allosteric activation mechanism. We agree that a more detailed investigation of the exact nature of the signal, and whether it differs or can be influenced *in vivo* represent an exciting direction for future work.

I am not convinced with the difference in PARP1 activation in Figure 6, which could perhaps be attributed to slight differences in protein amounts used – to my eye it seems like more PARP1 wt was used (intensity CBB stainings). Furthermore, even if we agree that in very early timepoints the mutants do not show activity like the wildtype, the question becomes whether this slightly slower kinetic has any biological impact? The PAR is still formed, albeit slightly slower. To increase impact of this work, authors should consider some simple biological tests such as recruitment to DNA damage. Is this impaired?

We would politely disagree with the reviewer as to the reliability of our kinetic data. We are confident that the observed differences are reproducible as the presented data are based on 3 independent measurements (see Source Data) showing the same trend. In addition, all quantitative analyses were normalised to account for differences in protein amounts as well as potential loss of auto-ADP-ribosylation sites in the ΔBRCT mutant. We employed also two independent approaches: signal establishment as well as NAD^+-degradation experiments, which consistently revealed the same trends.
We agree that the biological importance of these difference remains unresolved. We know that *h*PARP1 is recruited as one of the first factors (if not the first) to the DNA damage site and coordinates downstream events. Similarly, experiments in *A. nidulans* showed that fungal PARP1 plays a role in early DNA damage response. Impairment of this process could either trigger alternative repair pathway activation or reduce the efficiency of the response. Unfortunately, direct investigating these possibilities in *A. fumigatus* remains technically challenging. For example, laser microirradiation experiments, which help to investigate recruitment dynamics in mammalian cell culture, are not feasible in Aspergillus as (i) neither DAPI nor Hoechst are incorporated into *Af* at concentrations suitable for the experiment and (ii) the small size of the nucleus limits spatial resolution of recruitment events. To overcome these limitations, we performed some preliminary laser microirradiation experiments in mammalian cells but could not detect reliable recruitment most likely due to the presence of endogenous PARP1/2 or missing fungal co-factors. However, in our experiments we realised that *Af*-PARP1 associated with DNA/chromatin in mammalian cells and included experiments

demonstrating that this association is dependent on the BRCT domain (Fig. 4g, h, and Supplementary Fig. 7h, i).

Furthermore, the experiment A seems to be add odds with the NAD+ conversion assay in C: in A, the signal still increases after 10 minutes whereas in C the NAD+ is almost immediately used up. The authors should investigate the cause of this discrepancy.

The differences observed in the kinetics are due to the differences in experimental setup. The PARylation assay (Fig. 6a and b) for immunoblot utilises 500 μM NAD^+, whereas the NAD^+-depletion assay shown in Fig. 6c is limited to 10 μM NAD^+. We revised the text in the results section to make this distinction clearer to the reader.

It appears that AfPARP1 may behave more like human PARP3 than human PARP1, if it truly does not generate the "smeared" PAR chains that PARP1 makes. It might be worth to investigate this in more detail. Here it would also be of interest to determine in more detail what is modified by AfPARP1 and whether the BRCT domain changes this? The neutral hydroxylamine treatment would at 4 hours remove from more residues than just glutamate. This should be easy to test using specific hydrolyzing enzymes and the advantage here would be that the authors can also probe for MAR vs PAR using the right enzymes.

We thank the reviewer for this suggestion. To address this, we substantially expanded the biochemical characterisation of the *Af*-PARP1-derived modification and could show that *Af*-PARP1 is a true polyART. This conclusion is based on experiments using a panel of well characterised antibodies and detection reagents, mutation analysis as well as modification reversal by a panel of well-characterised (ADP-ribosyl)hydrolases (PARG, ARH1, ARH3, MacroD1, TARG1 and *Sau*Macro) with distinct linkage specificities (Supplementary Fig. 3).
Our results show that PARG converts the PAR into a MAR modification and that only MacroD1, TARG1 and *Sau*Macro have activity against the resulting mono-modification (Supplementary Fig. 3c). In addition, we also utilised our new MARylation producing *Af*-PARP1 mutant E657Q and observed a similar demodification pattern (Supplementary Fig. 3b) to modified WT *Af*-PARP1 co-treated with the different hydrolases and PARG. MacroD1 and TARG1 have well establish specificity against glutamate and aspartate linkages, while *Sau*Macro can in addition attack linkages to cysteine. However, the latter can be ruled out due to the MacroD1/TARG1 activity against the modification. As ARH1 and ARH3 also show no discernible activity linkages through arginine, serine, and tyrosine appear unlikely. Similarly, as the modification is removable by this set of enzymes attachment through histidine or lysine are also not likely. These finding are in agreement with our observations for the chemical de-ADP-ribosylation, which show modification removal under alkaline conditions as well as in presence of neutral hydroxylamine, which is indicative of an acetal ester linkage, that a modification of glutamate or aspartate residu(s).

Lastly, I would recommend to downtune some statements, as an example: "Given the molecular divergence from the mammalian pathway observed in our data, this signalling pathway emerges as a promising target for antifungal therapy." Presumably, the PARP

catalytic domains are highly similar – assumed from usage of an inhibitor developed for human PARPs (talazoparib). Also in human PARPs, the BRCT domain is important for DNA binding (Rudolph et al, Mol Cell 2021). This statement should be reconsidered, as (a) the current study does not address at all whether PARP1 is essential for fungi and (b) it is not that obvious that specifically targeting fungal PARP activity will be that easily achievable.

We agree with the reviewer that investigation into fungal PARP1s is still in the very early stages. At the same time, we have strengthened the biological component of the manuscript by generating a deletion mutant of *Af*-PARP1 and demonstrating attenuated virulence in the *Galleria mellonella* infection model (Fig. 1b). Together with earlier observation in *Magnaporthe oryzae* and *Fusarium oxysporum* f. sp. *niveum*, these findings suggest that fungal PARPs contribute to pathogenicity, although the underlying mechanism remain unclear. The divergence of the fungal BRCT from *h*PARP1 is also illustrated by the fact that while it may contribute to the monkeybar mechanism of genome scanning, the BRCT domain of *h*PARP1 is excluded from the multidomain assembly that constitutes the active PARP1 conformation. In contrast, our data support a model in which the BRCT domain of *Af*-PARP1 is an integral part of the active multidomain assembly. More broadly, fungal ADP-ribosylation signalling appears to differ from its mammalian counterpart at multiple levels. For example, fungi lack HPF1, a key regulator of mammalian PARP1/2 signalling, suggesting that both the nature of the ADP-ribosylation signal and its downstream processing may differ substantially from the human system.

With regard to inhibitor development, recent advances have demonstrated that selectivity can be achieved even between closely related *h*PARP1 and 2 enzymes. As *Af*-PARP1 is further diverged from either of human enzymes than they are from one other the development of fungal-selective inhibitors appears, at least in principle, feasible.
In summary, although further work is required, our findings support the view that fungal ADP-ribosylation pathway (either via *Af*-PARP1 or another component) warrants further investigation and holds potential to assist in antifungal therapy.

Minor comments
Line 206: the cited paper from the Luger lab showed that the BRCT domain binds intact DNA and proposed an intriguing model for its function (contributing to monkeybar mechanism). Considering the high relevance of this publication to the work submitted, the authors should expand more on what has been done in this prior work on the human BRCT domain. This is done in the discussion, but would fit earlier in the manuscript.

We agree that important and elegant work has been done by the Luger lab as well as others such as the Pascal and Ahel labs that opened the door to our understanding of the role of the BRCT domain in *h*PARP1. We have highlighted some of their recent work by extending the text in the "The BRCT domain of *Af*-PARP1 supports DNA binding" results section. We hope this addition improve the framing of our results and provide a clearer transition to the discussion section.

In tests of catalytic activity, 500µM NAD is used. Is NAD available in the model organism at such high levels? How would catalytic activity look at lower, presumably more physiological, concentrations?

We performed our experiment at a high NAD^+ concentration as we wanted to maximise reaction efficiency and facilitate detection of differences between mutants. However, we also performed experiments at lower concentrations and can confirm that the reaction occurs as expected. For example, the NAD^+ consumption assay (Fig. 8c) was performed at a concentration of 10 µM NAD^+. Both 500 µM and 10 µM are within the range of concentrations used routinely for *in vitro* experiments. Translating *in vitro* concentration to the *in vivo* NAD^+ availability, while interesting, is very challenging because local NAD^+ synthesis, consumption as well as local PARP concentrations can influence enzymatic activity in certain microenvironments, e.g. some reports suggest that the NAD^+ producer NMNAT1 supports *h*PARP1 activity in human cells through localised NAD^+ synthesis (Zhang, T. et al, JBC, 2012).

Figure 2a: it is not clear what “activated DNA” is and how this compares to the DNA oligonucleotides used in later figures.

The ‘activated DNA’ is a generic DNA activator sold by BPS Bioscience as part of their assay kits for *h*PARP1, 2, and 3 (cat. no. #80605). According to the manufacturer this product consists of DNAse-treated genomic DNA, hence contains a heterogenous mixture of DNA damage structures. To clarify this point, we now refer to it as “damaged genomic DNA” and have added the manufacturer information to both figure legend and methods section.

Figure 2: it would be interesting to have a direct comparison with human PARP1 (which is commercially available). The “PAR” formed here appears to be rather shorter than PAR chains typically observed after hsPARP1 reactions.

We agree that the PAR chains produced by *Af*-PARP1 appear to be generally shorter (as judged by smearing) than the ones of other polyARTs. This is one of the reasons we incorporated the comparison with *h*PARP2, *h*PARP3 and *Lo*PARP2 as the evolutionary closest and therefore most relevant homologues (see Fig. 1d). DNA binding and automodification assays of these proteins can be found in Supplementary Fig. 4c (EMSA) and 4e (automodification). In addition, we included *h*PARP2 in our antibody test panel, (Supplementary Fig. 3a) providing further support for this observation These comparisons highlight that *Af*-PARP1 produces substantially shorter ADP-ribose polymers than either *h*PARP2 or *Lo*PARP2, but a more smear like appearance than *h*PARP3, suggesting formation of short polymers. Together with the new experiments involving the aF helix double and triple mutants (Fig. 3), our results suggest that the chain length of *Af*-PARP1 is regulated by loss of DNA binding and/or tight regulation via autoinhibition and is not an inherent limitation of the catalytic domain.

Figure 6a: what are the distinct bands that are appearing, especially for example in PARP1delBRCT t=2 minutes, an interesting pattern appears in CBB which is not reflected in immunoblotting?

The band pattern was not reproducibly observed in independent experiments (see Source Data) and therefore most likely reflects a PAGE artifact. Since all samples originated from a single reaction mixture and were collected at different time points, a genuine reaction-dependent source would be expected to persist or accumulated over time and not be limited to a mid-experiment timepoint. Moreover, the pattern was only observed on the Coomassie-stained gel and not on the corresponding immunoblot, which was performed on a separate gel. As the quantitative analysis was derived from the IB data, we do not believe this artefact influences the interpretation of the experiment.

Model: would be good to also draw the human PARP1 to show the proposed difference between both systems

We agree with the reviewer and have now included a schematic of the monkeybar mechanism proposed for *h*PARP1 as Fig. 8a.

Sequence alignment extended figure 6: lack of conservation on sequence level doesn't mean there is no structural conservation.

We agree with the reviewer that there is no 1-to-1 equality between sequence and structural conservation. In contrast, one would expect on average higher structural than sequence conservation. We added a structural comparison figure (Supplementary Figure 7e) to show structural conservation between the BRCT domains of *Af*-PARP1, *h*PARP1 and *h*PARP4. We find that the overall fold is conserved. However, the r.m.s.d. scores are higher than for the other PARP domains analysed in this study and include alteration in secondary structure elements (Supplementary Fig. 7c and e) as well as low conservation of residues forming the DNA interaction surface. Together these observations suggest that, while the overall fold is maintained, the mode of DNA interaction has diverged between fungal and mammalian PARP BRCT domain homologues.

The methods section is lacking details in the plasmid construction paragraph. It is not clear exactly how constructs were generated, and how for example the codon optimization looks. This would be essential to be able to reproduce this work.

We have now included the synthetic codon optimised sequences for *Af*-PARP1 as well as the V5-mScarlet-I3 construct we utilised for the live cell microscopy (Supplementary Notes 2 and 3).

There are many typos, especially in the figure legends.

We thank the reviewer for noting this issue. The manuscript, figure legends, and supplementary materials have been carefully revised and typographical errors corrected throughout.

Reviewer #2

Bainbridge and colleagues report their analysis of fungal PARP enzymes and the contribution of the BRCT domain to DNA binding and catalytic activation of the PARP enzyme from A. fumigatus, a fungus considered a health concern. The study presents a phylogenetic analysis of PARP enzymes in the fungus kingdom, and then focus biochemical and structural work primarily on an A. fumigatus PARP that is expected to respond to DNA damage. They show that Af-PARP1 is activated by DNA damage of certain types, in particular DNA breaks bearing 5 prime phosphates, and also 3 prime DNA overhangs. The study identifies the BRCT domain of Af-PARP1 as contributing to the DNA interaction, and also the activation mechanism that leads to ADP-ribose modifications. The biochemical analysis is supported by predicted structures and experimental structures of homologous human PARPs. The study compares and contrasts Af-PARP with human homologs for which there is similar biochemical data. The study is clearly presented and the data is well done for the most part. There are a few areas that could be further developed to broaden the investigation and perhaps give deeper insights.

Ext. Data Figure 4e. The CD titration to quantitatively determine DNA binding affinity values needs more description in the methods, and it is not clear that the data is providing an accurate assessment of affinity. What are the units of the values in Figure 4e? The methods used uM and mM units, so it needs to be specified in the table. It is stated that DNAs were diluted to 14.5 uM and ligand (PARP protein) concentration was 0.269, 0.346, 0.336, or 0.433 mM depending on the ligand. It is thus not clear what was being titrated, since fixed values are given for both DNA and ligand. Ratios are given that indicate that protein was titrated, so it would seem that the mM concentrations given are simply the stock that was used, but this needs to be clarified. If the values in panel 4e are uM, then it does not seem that the titration is well-suited to accurately measure a KD, and it is clear that most data points are above the determined KD. The legend states that a Hill equation was used, but it seems that a more simple 1:1 binding model should be used base on the other analysis. What were the n values from the fit? A quantitative analysis of the binding affinities is important, in particular for comparing the contribution of the BRCT domain relative to the rest of Af-PARP2, but it is not clear that this assay is doing a good job. Is there a second assessment that can be used for quantification, like the EMSA assays? If the CD assay is used, it needs to be better developed and explained.

We thank the reviewer for the comment. During preparation of the revision, we identified a labelling error in Ext. Data Fig. 4e (which was supposed to show μM concentration with tick marks corresponding to 20, 40 and 60 μM). As suggested, we explored alternative fitting approaches as well as support the CD titration data with EMSA titration and quantification data.

The EMSA data broadly support our CD measurements. However, the in-gel behaviour of the complexes (see Supplemental Fig. 8d) did not allow reliable quantification. The increased smearing in the ΔBRCT samples indicate formation of an instable complex under the assay conditions thus giving mixed species (free/complexed DNA) results. In addition, we found that using different binding models for our CD titration data gave results that, while broadly confirmed our observations with the Hill equation, showed some unexplainable variability between fits. This could potentially reflect the contribution of multiple DNA interacting regions (e.g WGR and BRCT domain) and/or binding-induced conformational changes that influence binding behaviour (as suggested by the CD data).
Given these observations, we decided to take a conservative approach and excluded the quantitative CD analyses from the manuscript. The revised version now presents the EMSA data together with the qualitative CD analysis, which support the conclusions regarding the contribution of the BRCT domain to DNA binding while avoiding overinterpretation of the data.

Is it expected that Af-PARP1 will employ the same allosteric activation mechanism defined for human PARPs 1, 2, and 3? And if so, are there mutants or assays that could support this prediction? It is not clear what to expect from the AlphaFold predictions that are provided.

We thank the reviewer for this suggestion. To investigate whether *Af*-PARP1 employs an allosteric mechanism similar to human PARPs, we performed both a sequence and structural comparison with *h*PARP1 and 2 and subsequently constructed several mutants based on our finding. In particular we made (i) the N211A mutation, which is isostructural to the Asn residue in *h*PARP1/2 that transmits DNA binding into the HD domain, (ii) mutations of 3 acidic residues (D420, E423, and D427 in *Af*-PARP1; either as D420A/E423A double or D420A/E423A/D427A triple mutation) which reside in the αF helix, and are predicted to contribute to autoinhibition and (iii) E418A, a conserved residue found only in the fungi linage facing from the αF helix into the HD domain. These mutants were characterised using EMSA, PARP activity and DSF assays (Fig. 3 and Supplementary Fig. 3 and 6). Our results demonstrate that Asn211 is required for DNA-dependent activation as well as NAD^+ binding. The DEAA mutation show no or only mildly increased automodification in absence of DNA, but increased smearing in presence of DNA. In contrast, the triple mutant showed binding of BAD, a NAD+ analogue, even in absence of DNA as well as DNA-independent automodification thus suggesting a crucial contribution of Asp427 for allosteric autoinhibition of *Af*-PARP1. In contrast, E418A showed near WT behaviour in all our test except for a generally lowered T_m in our DSF assay indicating that the residue might be important for HD domain stability independently of the allosteric regulation.
Together, these findings support the existence of an allosteric activation mechanism in *Af*-PARP1 that shares key features with mammalian PARPs while retaining fungal-specific characteristics.

Does Af-PARP1 inactivate itself through self-modification (diminished DNA binding), similar to other DNA activated PARPs? And does the BRCT domain interact with ADP-ribose or poly-ADP-ribose?

While we could not directly test the DNA binding of automodified PARP, we observed that *Af*-PARP1 WT produces only short ADP-ribose polymers and release of the autoinhibition through our DEAA or DEDAAA mutants (see above) leads to formation of substantially longer PAR chains. This indicates that the autoinhibitory mechanism is responsible for limiting chain length. In addition, newly performed PAR-binding assays demonstrated that the *Af*-PARP1 is unable to bind PAR (Supplementary Fig. 7g). These observations suggest that the autoinhibition is triggered by DNA release from *Af*-PARP1.

Does Af-PARP1 produce mono or poly-ADP-ribose modifications? I don't think this was addressed, but the final model lists PAR and not MAR as the signal produced. Addressing this experimentally would be useful for understanding this system. The sequence analysis of the BRCT and WGR domains should be extended to the catalytic region (HD and ART).

We performed new experiment to test whether *Af*-PARP1 produces PAR or MAR or a mixture of both. This is discussed extensively in our answer to reviewer #1. In brief, *Af*-PARP1 is a PAR generating enzyme with sequential/structural features comparable to *h*PARP1/2 and none of the features that limit *h*PARP3 to MAR (Supplementary Fig. 2, 3a-c, 6). In addition, we performed immunoblot analysis using a MAR and PAR specific antibodies and while we cannot fully rule out that both are present (as the MAR antibodies had some cross-reaction also wit *h*PARP2), we could determine that PAR was definitely present. This is further support by our E657Q mutation, that mimics the *h*PARP1 E988Q, which is limited to MAR productions as well as our (ADP-ribosyl)hydrolase assays that show that PARG removed the bulk of the signal, but left a MAR modification on *Af*-PARP1, which could be removed by human MacroD1 and TARG.

What is the significance of the binding to overhang DNA? Is there a particular length that is ideal for the overhang? Was binding to single-strand DNA tested?

We performed additional binding and activation experiments using DNA molecules containing overhangs of varying length as well as single-stranded DNA. Our results revealed that a minimal overhang length of 4 nt was required for catalytic activation, while binding was observed independently of overhang length (Supplementary Fig. 4a and d). Single-strand DNA shows only limited binding to *Af*-PARP1 and fails to activate the enzyme (Supplementary Fig. 4 c-d).

CBB acronym is not defined anywhere that I could find.

We added the abbreviation to the text as well as in the figure legend showing a Comassie Brilliant Blue (CBB) stained PAGE.

Reviewer #3

The paper provides an evolutionary analysis of the fungal PARPs and their domain architecture and then focuses on Af-PARP1, a homolog of human PARP1/2. They use EMSA

and activity assays to show that Af-PARP1 is catalytically responsive to DNA damage but despite its closer relation with animal and plant PARP2, it binds DNA and is activated like animal PARP3. They investigate the Af-PARP1 WGR domain (present in human PARP1, 2, and 3) and show that Af-PARP1 (unlike human PARP2) is not activated by blunt ends despite high conservation of their WGR domains. Conversion of the single non-conserved residue in Af-PARP1 WGR to that of human PARP2 did not affect its binding to blunt ends. Additional mutational analysis showed that Af-PARP1 has a 5'-phosphate sensing mechanism conserved across zinc finger-independent PARPs like human PARP2 and 3. They next investigate the Af-BRCT domain (present in human PARP1 not PARP2 or 3) and show that deletion severely restricts the DNA-binding profile. SAXS analysis shows that upon DNA binding, the BRCT domain transitions from behaving as an independent domain to forming an integral part of the multidomain WGR-HD-ART assembly, a behaviour that contrasts with that of human PARP1. Time course analysis shows Af-PARP WT is activated by both nicked and gapped DNA, whereas ΔBRCT is activated by nicked DNA but reduced by gap DNA. Lysine mutations in BRCT retained the ability to bind nicked DNA but showed reduced binding and activation by gap DNA. Further analysis of one mutant, K52/60A, showed it behaved like WT in response to nicked DNA but was reduced in binding, activation, and NAD+ consumption in response to gapped DNA. Altogether showing that the BRCT domain can broaden the range of DNA damage structures recognized by Af-PARP1. They propose a model where the BRCT and WGR domains act cooperatively to scan and bind DNA and activate PARylation.

This is a clear study characterizing fungal PARPs. The comprehensive phylogenetic analysis should provide a roadmap for future studies of fungal PARPs. The in vitro data are of high quality and support the conclusions drawn. I have no concerns with the data other than a couple of minor technical points below. What is less clear is how this work will impact the field. The architecture of Af-PARP1, is not so much "unique" as it is just a different configuration of known motifs. The details of how the motifs influence recognition and binding to DNA damage differ from previous examples but not so dramatically as to have a great impact. For example, it is shown that the Af-BRCT domain contributes to the WGR-HD-ART assembly, whereas for human PARP1, the BRCT may not be involved. Since the concept of assembly has already been well-documented with human PARP1, this result is interesting but does not provide new insights. Insight into fungal PARPs in general could have important implications for antifungal therapy. However, it is not yet known if PARPs are important for fungal virulence. Thus overall, while this is a well-done interesting study, its descriptive nature and narrow focus may not appeal to a broad audience.

We thank the reviewer for the positive assessment of the experimental quality of our study and raising the important question of its broader impact. While an in-depth characterisation of the physiological role of *Af*-PARP1 is beyond this study, we constructed a *Af* knockout strain in the AfS77 background and assessed its virulence in the *Galleria mellonella* infection model (Fig. 1b). These experiments revealed attenuated virulence of the knockout strain (Fig. 1b), providing direct evidence that *Af*-PARP1 contributes to fungal pathogenicity. In addition, while DNA recruitment and occupancy experiments are currently technically not feasible in *Af*, the revised manuscript includes new mechanistic analysis of *Af*-PARP1 regulation, including

investigation of allosteric activation, characterisation of the ADP-ribosylation product, analysis of chromatin association, and examination of BRCT-dependent DNA recognition. In addition, we reported that in murine 2H11 cells the BRCT domain influence chromatin association, thus supporting our model of the BRCT domain as both a genome scanning as well as activation domain, which is a clear difference to the human system.
Together, these additions extend the study beyond a descriptive characterisation of fungal PARPs and provide new insight into how fungal PARP signalling differs from mammalian systems.

1. There is a discrepancy between the text in the Results section and the Figure (2):
Line 142 describes DNA binding "Fig. 2b" however the DNA binding is shown in Fig. 2c.
Line 155 describes catalytic activity "Fig. 2c" however catalytic activity is shown in Fig. 2b.

We apologies for the mistake and can confirm that after re-ordering the figures for the revision the EMSA assay showing *Af*-PARP1-DNA binding is now Fig. 2b and the activation assay Fig. 2c. The text reflects these changes.

2. I cannot find a description in the text of the Bd-PARP K80A mutant shown in Fig. 5C.

We edited the text to highlight that K80A has a similar effect to the BRCT domain deletion.

RESPONSE TO REVIEWERS' COMMENTS

Reviewer #1 (Remarks to the Author):

The authors have responded well to the reviewers comments and suggestions. I do not have further remarks.

Reviewer #2 (Remarks to the Author):

In this resubmission, the authors have addressed the concerns that I raised during the initial review. They have added several experiments that make for a more complete description of the biochemistry of Af-PARP1.

minor comments:

I could not find the source of the compound BAD in the methods section.

We added the source of the compound BAD to the "Differential Scanning Fluorimetry (DSF)" section within the Methods. It reads now "[…] and/or 1 mM BAD (Sigma-Aldrich, cat no. SML3494) […]"

Supplemental legends: please include primary publication references (if available) for PDB structures used in figures (supplementary figures 5c,g and 6a and 7e.)

We apologise for the unintentional omission and have added PDB codes for the used structures as well as citation references to the corresponding papers where available. Please note that PDB 2COK (*h*PARP1 BRCT domain) and PDB 2EOC (*h*PARP3 WGR domain) have only be deposited and have no associated manuscripts.
