# Supplementary material for: The BRCT domain enhances DNA binding and catalytic efficiency of fungal PARPs
Source: Nat Commun. 2026 Jul 24;17:9024. doi: 10.1038/s41467-026-75844-6 (PMC13507078; doi:10.1038/s41467-026-75844-6)

Figure 2 Unprocessed gels and blots

Figure 2a

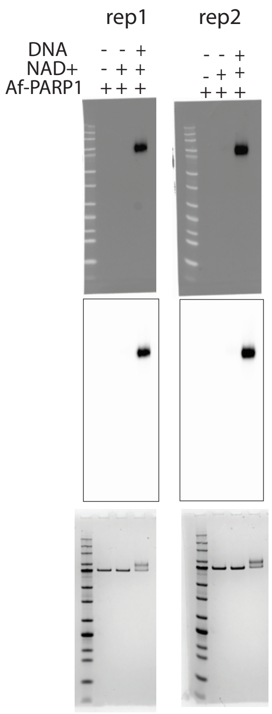

Figure 2c *red box = 2a (rep 3)

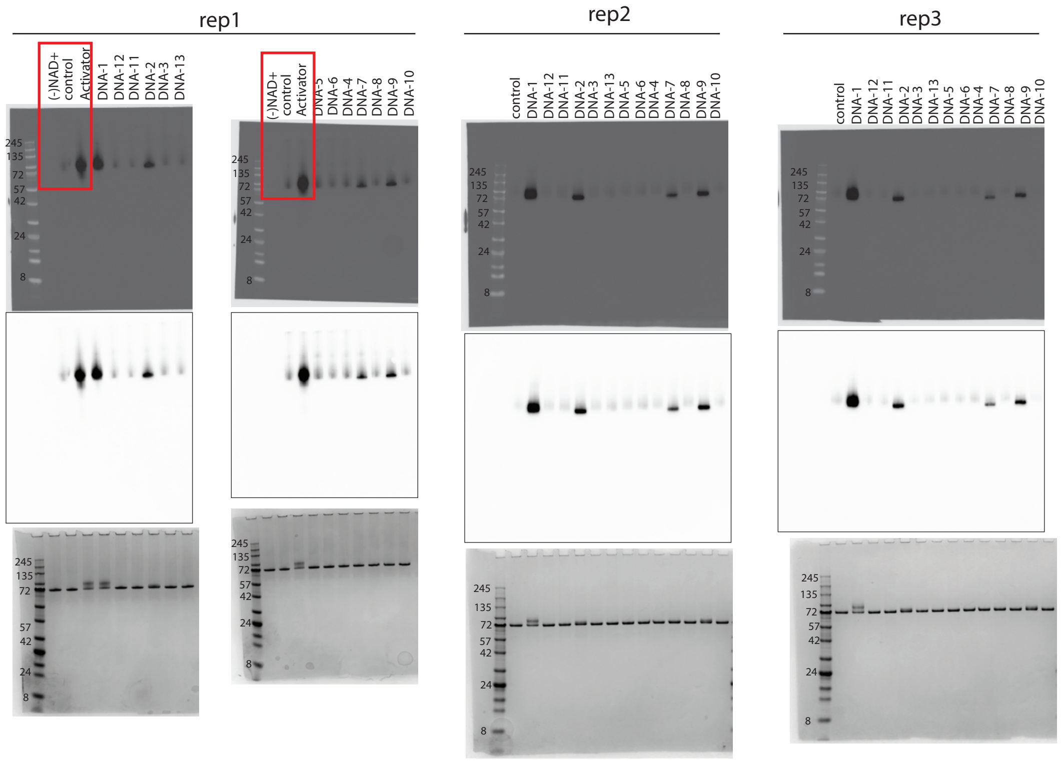

Figure 2b

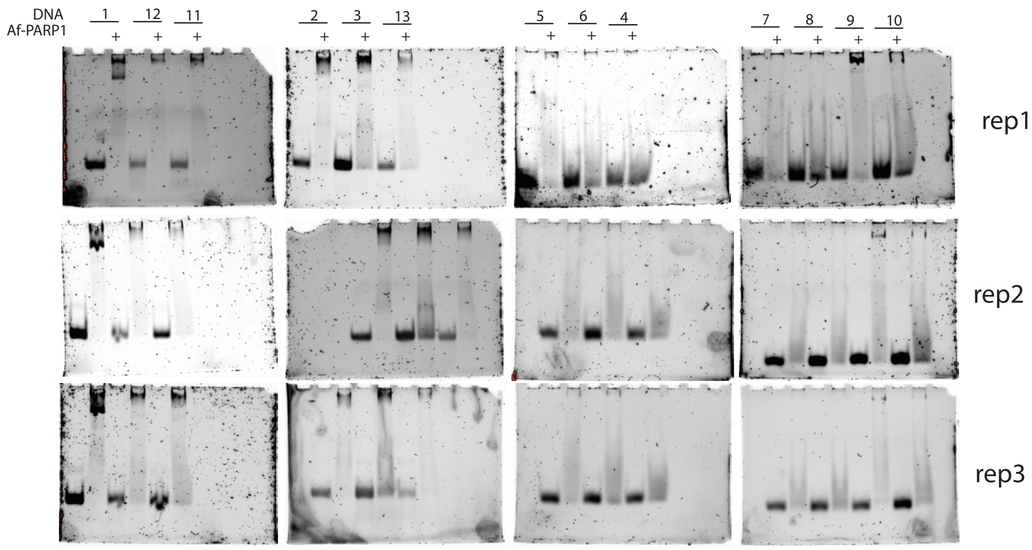

Figure 2d

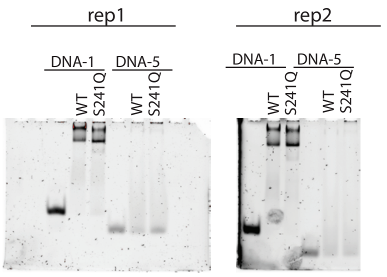

Figure 2e

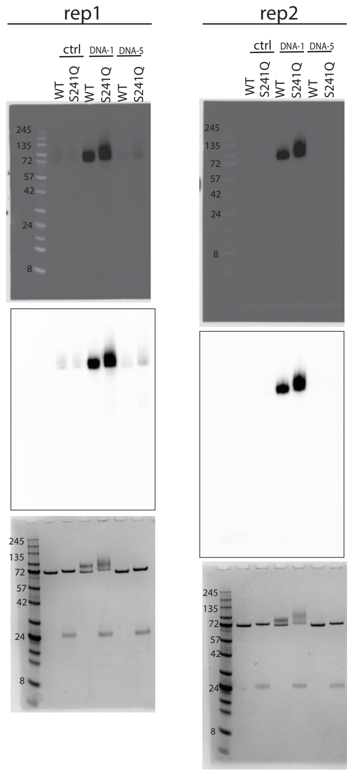

Figure 2g

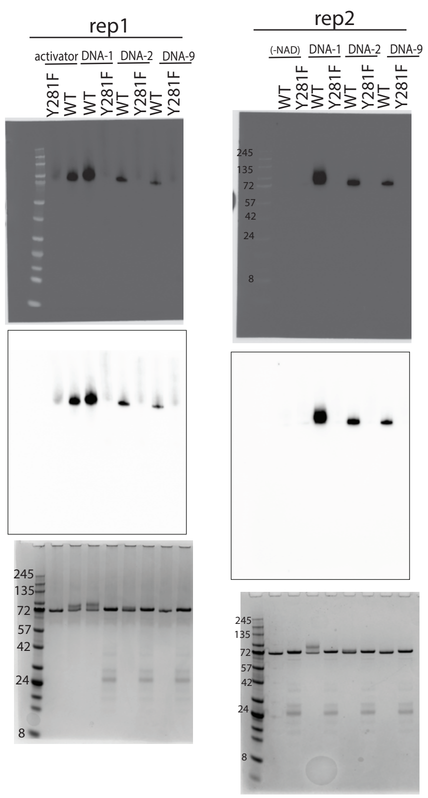

Figure 2f

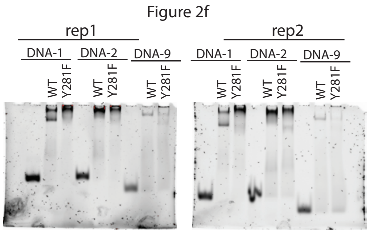

Supplement: Supplementary file 4 [file 41467_2026_75844_MOESM4_ESM.zip › Source Data/Source Data - Main Figures/Figure 2 Unprocessed Gels and Blots.pdf]
