# Supplementary material for: The BRCT domain enhances DNA binding and catalytic efficiency of fungal PARPs
Source: Nat Commun. 2026 Jul 24;17:9024. doi: 10.1038/s41467-026-75844-6 (PMC13507078; doi:10.1038/s41467-026-75844-6)

Supplementary figure 9 unprocessed gels and blots

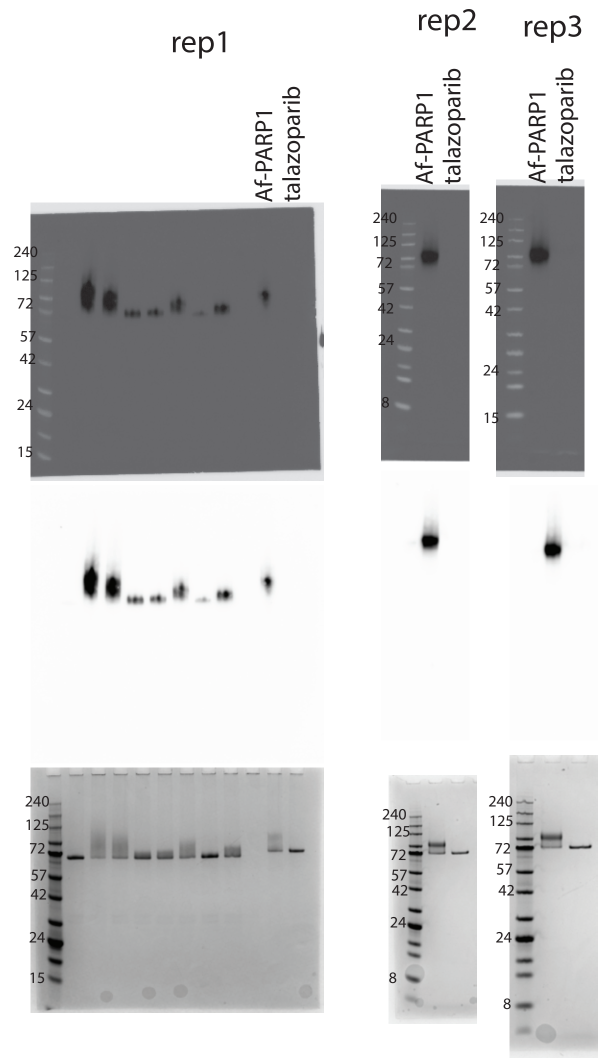

Supplement: Supplementary file 4 [file 41467_2026_75844_MOESM4_ESM.zip › Source Data/Source Data - Supplementary Figures/Supplementary Figure 9 unprocessed gels and blots.pdf]
